# Supplementary material for: Emergence of oncofetal plasticity is ubiquitous in early colorectal cancers
Source: Nature. 2026 Apr 15;654(8117):229–39. doi: 10.1038/s41586-026-10344-7 (PMC13233332; doi:10.1038/s41586-026-10344-7)
Supplement: Supplementary file 1 — This file contains Supplementary Figs. 1–3. [file 41586_2026_10344_MOESM1_ESM.pdf]

nature portfolio

https://doi.org/10.1038/s41586-026-10344-7

Supplementary information

Emergence of oncofetal plasticity is ubiquitous in early colorectal cancers

In the format provided by the
authors and unedited

Emergence of oncofetal plasticity is ubiquitous in early colorectal cancers

Julian R. Buissant des Amorie[1,2,3#], Joris H. Hageman[1,2,3#], Sascha R. Brunner[1,2,3#], Suzanne E.M. van der Horst[1,2,3#], Maria C. Puschhof[1,2], Arne van Hoeck[1,2], Inge van Lierop[1,2,3], Sjors Middelkamp[1,2,3], Lisa van der Schee[4], Sven van Kempen[4], Folkert Morsink[4], Robin Geene[1,5], Sander Mertens[1,2,3], David S. Cavigelli[1,2,3], Ingrid Verlaan-Klink[1,2,3], Lianne J. Kraaier[6], Jorieke Salij[7], Renate Bezemer[7], Onno Kranenburg[7], Miangela M. Laclé[4], Leon M.G. Moons[8], Hugo J.G. Snippert[1,2,3*]

[1] Center for Molecular Medicine, University Medical Center Utrecht, Utrecht, the Netherlands
[2] Oncode Institute, Utrecht, the Netherlands
[3] Present address: Princess Máxima Center for Pediatric Oncology, Utrecht, the Netherlands
[4] Department of Pathology, University Medical Center Utrecht, Utrecht, the Netherlands
[5] Utrecht Sequencing Facility, University Medical Center Utrecht, Utrecht, the Netherlands
[6] Independent researcher, Utrecht, the Netherlands
[7] Utrecht Platform for Organoid Technology, Utrecht, the Netherlands
[8] Department of Gastroenterology and Hepatology, University Medical Center Utrecht, Utrecht, the Netherlands
[#] These authors contributed equally to this work
* Correspondence: h.j.g.snippert-2@prinsesmaximacentrum.nl

Supplementary Information

This file contains:

Supplementary Figure 1 page 2
Supplementary Figure 2 page 3
Supplementary Figure 3 page 4
Description of additional Supplementary Files page 5

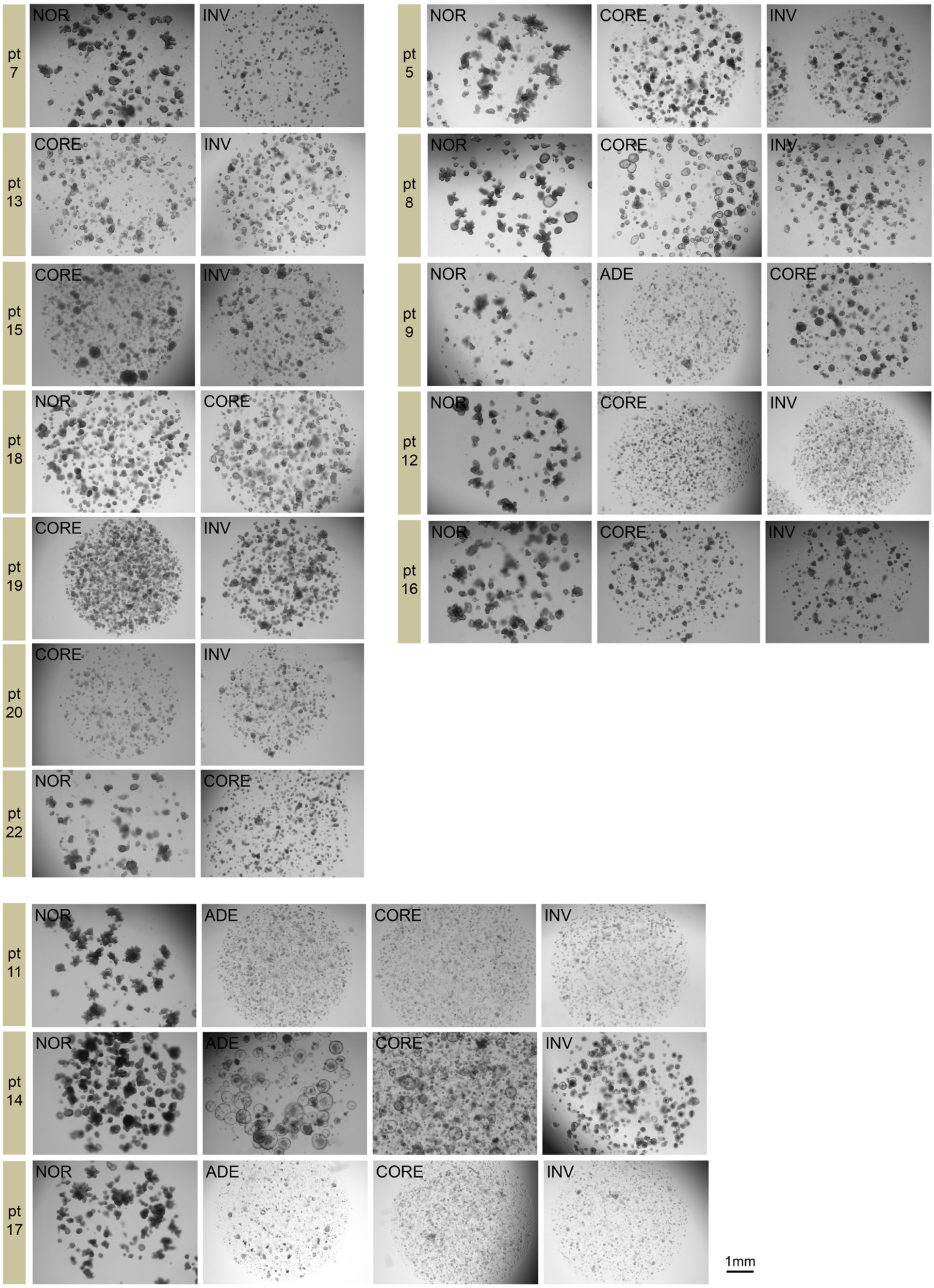

Supplementary Figure 1 | Morphology of organoids in multiregional biobank of early-stage CRC
Brightfield images of early passage organoid cultures showing distinct morphological characteristics of cancer organoids compared to normal. Images were generated with EVOS imaging system. Related to Figure 2.

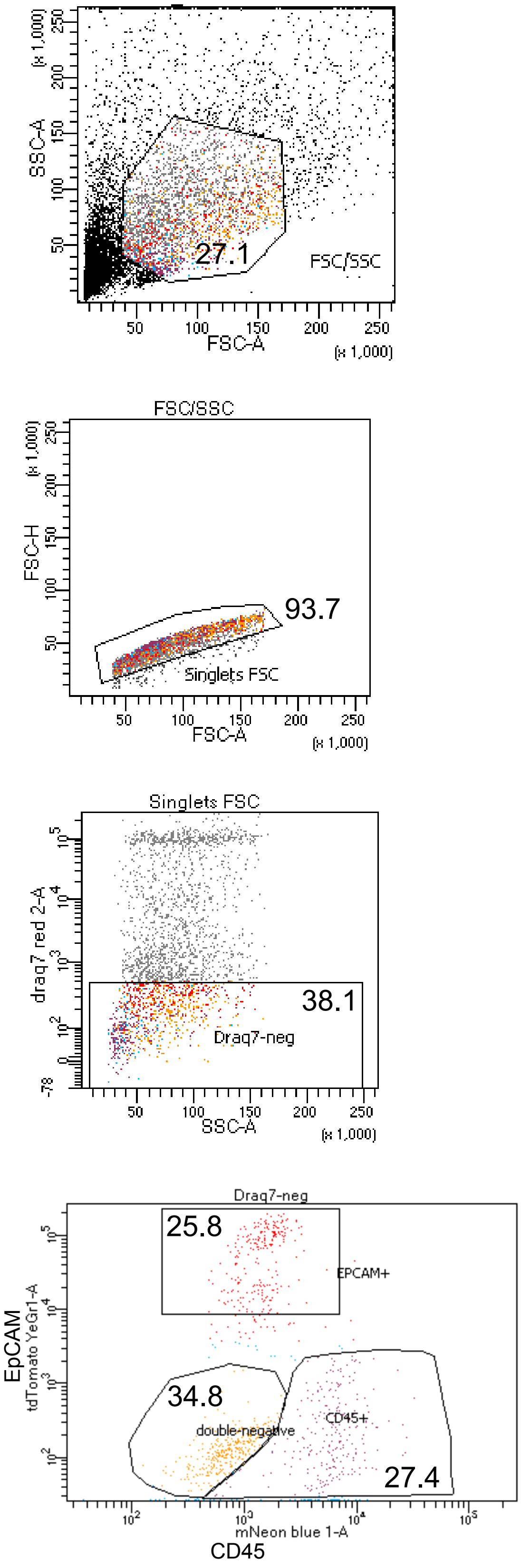

Supplementary Figure 2 | Flow cytometry gating strategy for sorting of single cells from primary CRC biopsies. Example of gating strategy used during sorting for plate-based scRNA-seq. Related to Figure 3.

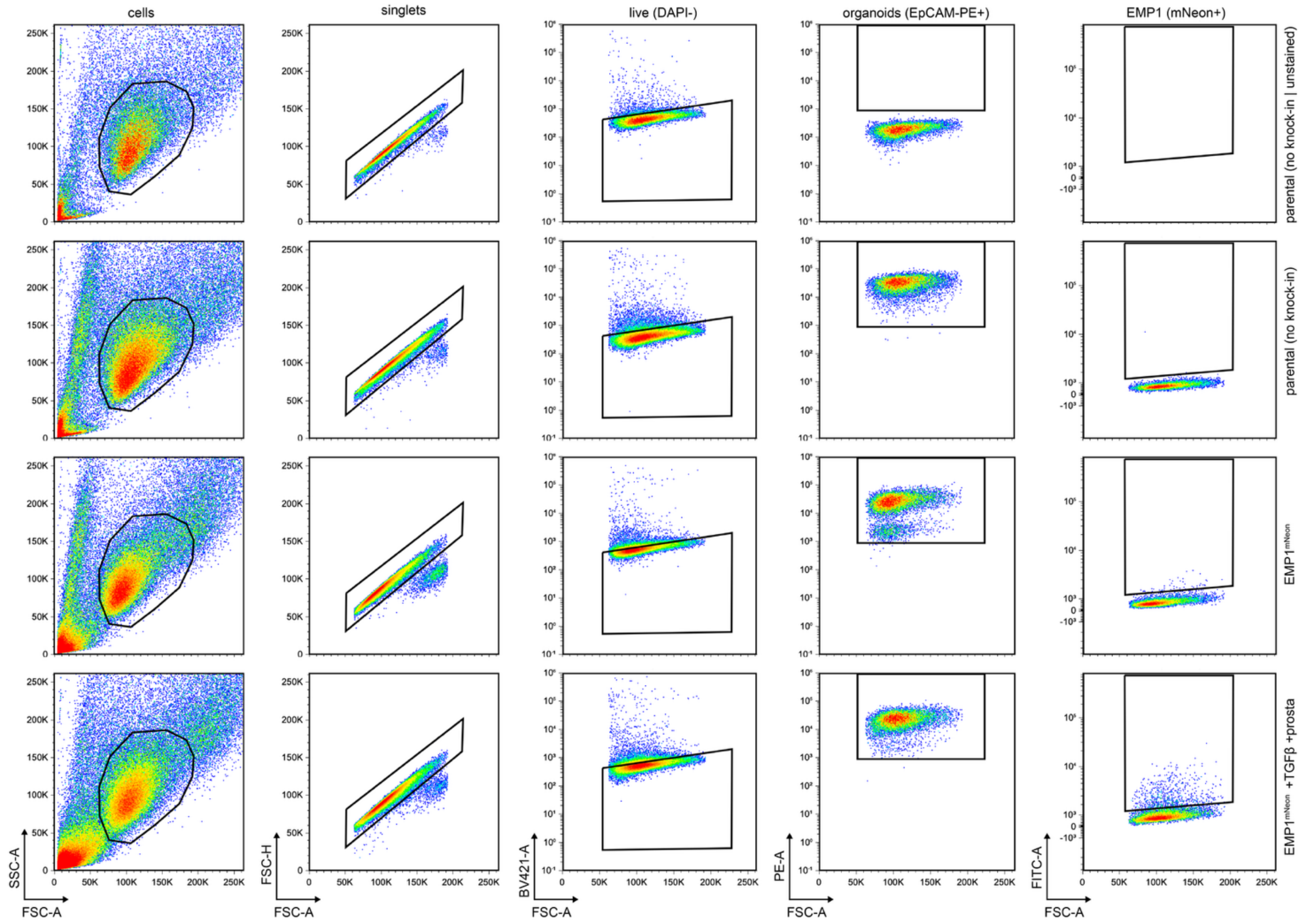

Supplementary Figure 3 | Flow cytometry gating strategy for EMP1-mNeon. Example of gating strategy for quantification of EMP1-mNeon+ cells in reporter organoids. Related to Figure 4.

Description of additional Supplementary Files

Supplementary Table 1
Clinical data of patient samples used in this study.

Supplementary Table 2
Invasive front and core tumor cell signatures derived from Nanostring GeoMx spatial transcriptomics (WTA, top 100 differentially expressed genes from panCK positive segments in tumor core (CORE) vs. invasive front (INV), linear mixed effect model, ranked by log2 fold-change, FDR < 0.05).

Supplementary Table 3
Gene signatures used in analyses.

Supplementary Table 4
Specific driver mutations of patients based on WGS.

Supplementary Table 5
scRNA-seq marker gene table of early-stage CRC tumor microenvironment. Table contains the output of the FindAllMarkers() function from the Seurat R package (log2 fold-change > 0.5, p-value < 0.01).

Supplementary Table 6
RNA-seq differential expression analysis of organoids from co-culture experiment. Table contains batch-corrected pairwise comparisons between the indicated experimental groups: monoculture (None), co-culture with fibroblasts on plastic (2D) and co-culture with fibroblasts in Matrigel (3D).

Supplementary Table 7
RNA-seq differential expression analysis of fibroblasts from co-culture experiment, cultured on plastic (2D) or in Matrigel (3D).

Supplementary Table 8
Sequences of qPCR primers.

Supplementary Report 1
Nanostring GeoMx CTA quality control report in HTML format.

Supplementary Report 2
Nanostring GeoMx WTA quality control report in in HTML format.
