# Supplementary material for: Emergence of oncofetal plasticity is ubiquitous in early colorectal cancers
Source: Nature. 2026 Apr 15;654(8117):229–39. doi: 10.1038/s41586-026-10344-7 (PMC13233332; doi:10.1038/s41586-026-10344-7)
Supplement: Supplementary file 2 — Reporting Summary [file 41586_2026_10344_MOESM2_ESM.pdf]

nature portfolio

Corresponding author(s): Hugo Snippert
Last updated by author(s): 15-02-2026

Reporting Summary

Nature Portfolio wishes to improve the reproducibility of the work that we publish. This form provides structure for consistency and transparency in reporting. For further information on Nature Portfolio policies, see our Editorial Policies and the Editorial Policy Checklist.

Statistics

For all statistical analyses, confirm that the following items are present in the figure legend, table legend, main text, or Methods section.

n/a	Confirmed	
☐	☒	The exact sample size (*n*) for each experimental group/condition, given as a discrete number and unit of measurement
☐	☒	A statement on whether measurements were taken from distinct samples or whether the same sample was measured repeatedly
☐	☒	The statistical test(s) used AND whether they are one- or two-sided<br>*Only common tests should be described solely by name; describe more complex techniques in the Methods section.*
☒	☐	A description of all covariates tested
☐	☒	A description of any assumptions or corrections, such as tests of normality and adjustment for multiple comparisons
☐	☒	A full description of the statistical parameters including central tendency (e.g. means) or other basic estimates (e.g. regression coefficient) AND variation (e.g. standard deviation) or associated estimates of uncertainty (e.g. confidence intervals)
☐	☒	For null hypothesis testing, the test statistic (e.g. *F*, *t*, *r*) with confidence intervals, effect sizes, degrees of freedom and *P* value noted<br>*Give P values as exact values whenever suitable.*
☒	☐	For Bayesian analysis, information on the choice of priors and Markov chain Monte Carlo settings
☒	☐	For hierarchical and complex designs, identification of the appropriate level for tests and full reporting of outcomes
☐	☒	Estimates of effect sizes (e.g. Cohen's *d*, Pearson's *r*), indicating how they were calculated

Our web collection on statistics for biologists contains articles on many of the points above.

Software and code

Policy information about availability of computer code

Data collection

• FACS Celesta (for EMP1-mNG organoid quantifications): BD FACS DIVA v9.2
• FACSaria III (for sorting cells for scRNA-seq): BD FACS DIVA v.8.0.1
• Microscopy Leica SP8 (for imaging EMP1-mNG organoids): LAS X v3.5.7.23225
• qPCR: CFX Manager Version 3.1.1517.0823.

Data analysis

Nanostring GeoMx:
• Fgsea v1.24.0
• ssGSEA (implemented in GSVA v1.46.0)
• CMScaller v2.0.1
• inferCNV v1.14.2
• NicheNet v2.0.0
• VariancePartition v1.38.1

WGS:
• oncoanalyser v1.0.0
• HaplotypeCaller v4.1.3
• SMuRF v3.02
• ape v5.8

scRNA-seq:

- nf-core scrnaseq pipeline v2.4.0
- STARsolo v2.7.10b
- STARsolo v2.7.10b
- Seurat v5.0.1
- Monocle3 v1.4.26
- CytoTRACE2 v1.1.0
- Slingshot v2.16.0
- CellRank v2.0.7

Nanostring CosMx:
- Seurat v5.0.1
- Sctransform

Bulk RNA-seq
- nf-core RNAseq pipeline v3.14.0
- FastQC v0.12.1
- Trim Galore! v0.6.7
- STAR v2.7.9a
- Salmon v1.10.1
- DESeq2 v1.38.3
- clusterProfiler (4.8.3)

Flow cytometry:
- Floreada v8/6/24
- DIVA version v9.2

Microscopy:
- OrganoSeg v1
- Stardist v0.3.0
- FIJI ImageJ 2.14.0/1.54f
- QuPath v0.6.0

General:
- Rstudio 2024.12.1+563
- R v4.2.0
- Excel 16.95
- Prism 10.4.1
- ggplot2 v3.5.1
- ggpubr v0.6.0

For manuscripts utilizing custom algorithms or software that are central to the research but not yet described in published literature, software must be made available to editors and reviewers. We strongly encourage code deposition in a community repository (e.g. GitHub). See the Nature Portfolio guidelines for submitting code & software for further information.

Data

Policy information about availability of data

All manuscripts must include a data availability statement. This statement should provide the following information, where applicable:

- Accession codes, unique identifiers, or web links for publicly available datasets
- A description of any restrictions on data availability
- For clinical datasets or third party data, please ensure that the statement adheres to our policy

Whole genome sequencing data of patient-derived organoids (EGAD50000002204), RNA sequencing data of organoids and organoid-fibroblast co-cultures (EGAD50000002202) and scRNA-sequencing data of early-stage colorectal cancers (EGAD50000002203) are available through the European Genome-Phenome Archive (EGA) under accession number EGAS50000001532. Bulk (Nanostring GeoMx) and single-cell (Nanostring CosMx) spatial transcriptomic data of T1 colorectal cancers and processed expression data (RNA-seq of organoids and organoid-fibroblast co-cultures and scRNA-seq of early-stage colorectal cancer biopsies) are available at Zenodo (https://doi.org/10.5281/zenodo.17671259). Expression data (scRNA-seq, Nanostring GeoMx CTA, Nanostring CosMx Fig.3) can be accessed through an interactive dashboard (https://snippertlab.nl/resources). Published scRNA-seq data of human colorectal cancers were obtained from GSE144735 and GSE13246545; GSE20134955; and GSE17834154.

Research involving human participants, their data, or biological material

Policy information about studies with human participants or human data. See also policy information about sex, gender (identity/presentation), and sexual orientation and race, ethnicity and racism.

Reporting on sex and gender	Information on sex is reported in Supplementary Table S1. Out of the 16 patients included for organoid derivation, 8 (50%) were male and 8 (50%) were female. All patients gave informed consent to share this information.
Reporting on race, ethnicity, or other socially relevant groupings	Information on race and ethnicity has not been collected

Population characteristics	Relevant clinical information for the patients is listed in Supplementary Table 1. The organoid/fibroblast biobank consists of 16 early-stage colorectal cancer patients. Patients were aged between 60 and 81. Patients were not pre-treated. For GeoMx, 10 T1 colorectal cancers (5x with lymph node metastases and 5x without lymph node metastases) were analyzed using the GeoMx CTA (Cancer Transcriptome Atlas) panel and 9 T1 colorectal cancers (3x without metastases, 3x with lymph node metastases, and 3x with distant metastases) were analyzed using the GeoMx WTA (Whole Transcriptome Atlas) panel. For the tumor specimens analyzed by CTA, samples were selected such that risk factors, including lymphovasclular invasion and tumor budding and location and morphology were similar between metastatic and non-metastatic primary tumors within the CTA cohort. For Nanostring CosMx 1 T1 patient from the organoid biobank (pt5) was selected for single-cell spatial transcriptomics of a complete tumor cross section. Additionally, 11 CRC specimens capturing the moment of malignant transformation at the start of invasive tumor growth (3x tumor in situ, 5x T1 sm1 and 3x T1 sm3) were selected for Nanostring CosMx and sectioned to fit on 2 slides.
Recruitment	The biobank participants were 16 patients suspected of early-stage colorectal cancer who underwent surgery for removal of the primary tumor, instead of endoscopic removal due to inaccessibility of the tumor. This could skew the sampled tumors towards tumors that are difficult to access by endoscopy. Patients were recruited by the Utrecht Platform for Organoid Technology (UPORT) (https://uport.umcutrecht.nl/researcher/en/).
Ethics oversight	This study was approved by the University Medical Centre (UMC) Utrecht ethical committee

Note that full information on the approval of the study protocol must also be provided in the manuscript.

Field-specific reporting

Please select the one below that is the best fit for your research. If you are not sure, read the appropriate sections before making your selection.

☒ Life sciences ☐ Behavioural & social sciences ☐ Ecological, evolutionary & environmental sciences

For a reference copy of the document with all sections, see nature.com/documents/nr-reporting-summary-flat.pdf

Life sciences study design

All studies must disclose on these points even when the disclosure is negative.

Sample size	There was no statistical calculation of sample size upfront. To establish a biobank representative of early-stage CRC, we aimed to include all eligible patients during the study period, targeting a minimum of 10 participants. Ultimately, 16 patients were enrolled in the biobank. For the Nanostring GeoMx and CosMX, sample size (19 and 11 patients, respectively) was determined based on previous knowledge of the sequencing facility on experimental variation. Sample sizes of experiments are indicated throughout the manuscript.
Data exclusions	In scRNA-seq, epithelial cells were excluded due to low sequencing quality (based on the total number of transcripts per cell and mitochondrial transcripts per cell). In Nanostring GeoMx, 1 patient was excluded because it was classified as stage T3, whereas all other patients were stage T1.
Replication	All experiments were performed in multiple independent replicates, as described in the figure legends and Methods section.
Randomization	Experimental groups for the in vivo analyses were not based on randomization, but on pathological classification of normal, adenoma, tumor core, and invasive front. Organoids and fibroblasts used in the in vitro experiments were randomly assigned to an experimental group.
Blinding	The researchers were blinded to patient identities. In the experiments, no blinding of researchers for organoid identities or treatments was performed, to avoid sample swaps and as is according to common practice in the field.

Reporting for specific materials, systems and methods

We require information from authors about some types of materials, experimental systems and methods used in many studies. Here, indicate whether each material, system or method listed is relevant to your study. If you are not sure if a list item applies to your research, read the appropriate section before selecting a response.

Materials & experimental systems

n/a	Involved in the study	
☐	☒	Antibodies
☐	☒	Eukaryotic cell lines
☒	☐	Palaeontology and archaeology
☒	☐	Animals and other organisms
☒	☐	Clinical data
☒	☐	Dual use research of concern
☒	☐	Plants

Methods

n/a	Involved in the study	
☒	☐	ChIP-seq
☐	☒	Flow cytometry
☒	☐	MRI-based neuroimaging

Antibodies

Antibodies used	The following antibodies were used for immunohistochemistry: SFRP2 (PA5-29390, Invitrogen, 1:200), LAMC2 (AMAb91098, Atlas Antibodies, 1:500), PanCK (AlexaFluor 532 conjugated; NBP2-33200 Novus 1:500, and NBP3-08398 Novus 1:300), DNA SytoTM 13 (S7575, Invitrogen, 1:10k), Alexa 594 anti-rabbit (Invitrogen A11037; 2 μg/ml) and Alexa 594 anti-mouse (Invitrogen A11032; 2 μg/ml)<br><br>The following antibodies were used for Nanostring: Pan-Cytokeratin (PanCK, Novus Biologicals NBP2-33200AF532, 1 μg/ml) and CD45 (Novus Biologicals NBP2-34528AF594, 5 μg/ml) and DNA SytoTM 13 (S7575, Invitrogen, 500 nM).<br><br>The following antibodies were used for tissue staining for scRNA-seq: PE anti-human CD326 (EpCAM) (324205 9C4, Biolegend, 1:200) and FITC anti-CD45 (368507 2D1, Biolegend, 1:200)<br><br>The following antibodies were used for staining of organoids: PE anti-human HLA A/B/C (311405 W6/32, Biolegend, 1:400), PE anti-human CD326 (EpCAM) (324205 9C4, Biolegend, 1:400); beta-catenin (C2206, Sigma-Aldrich, 1:500), LAMC2 (AMAb91098, Atlas Antibodies, 1:500)., 647 anti-mouse (Invitrogen A21236; 1:500) and Alexa 568 anti-rabbit (Invitrogen A11011; 1:1,000).
Validation	All antibodies were validated for species reactivity and application by the manufacturer, as specified below:<br><br>SFRP2: verified for human immunohistochemistry and immunofluorescence<br>LAMC2: verified for human immunohistochemistry and immunofluorescence<br>PanCK: verified for human immunohistochemistry and immunofluorescence<br>CD45 (Novus Biologicals): verified for human immunohistochemistry<br>EpCAM: verified for human flow cytometry<br>CD45 (Biolegend): verified for human flow cytometry<br>HLA: verified for human flow cytometry<br>beta-catenin: verified for human immunofluorescence<br><br>Additional validation was performed by confirming the expected localization and expression patterns.

Eukaryotic cell lines

Policy information about cell lines and Sex and Gender in Research

Cell line source(s)	All cell lines (organoids and fibroblasts) used were generated in this study as described in the methods. Patient information is listed in Supplementary Table 1.
Authentication	Organoids were subjected to whole-genome sequencing and functionally tested in growth factor dependency screens. This way, the most used organoids could be authenticated based on their specific mutation in driver genes (TP53, KRAS) and on their growth factor dependency. Additionally, organoid morphology (see Extended Data Fig. 3) was continuously monitored to prevent sample swaps.
Mycoplasma contamination	Cell lines were routinely tested for mycoplasma and results were always negative.
Commonly misidentified lines<br>(See ICLAC register)	None

Plants

Seed stocks	*Report on the source of all seed stocks or other plant material used. If applicable, state the seed stock centre and catalogue number. If plant specimens were collected from the field, describe the collection location, date and sampling procedures.*
Novel plant genotypes	*Describe the methods by which all novel plant genotypes were produced. This includes those generated by transgenic approaches, gene editing, chemical/radiation-based mutagenesis and hybridization. For transgenic lines, describe the transformation method, the number of independent lines analyzed and the generation upon which experiments were performed. For gene-edited lines, describe the editor used, the endogenous sequence targeted for editing, the targeting guide RNA sequence (if applicable) and how the editor was applied.*
Authentication	*Describe any authentication procedures for each seed stock used or novel genotype generated. Describe any experiments used to assess the effect of a mutation and, where applicable, how potential secondary effects (e.g. second site T-DNA insertions, mosiacism, off-target gene editing) were examined.*

Flow Cytometry

Plots

Confirm that:

☒ The axis labels state the marker and fluorochrome used (e.g. CD4-FITC).

☒ The axis scales are clearly visible. Include numbers along axes only for bottom left plot of group (a 'group' is an analysis of identical markers).

☒ All plots are contour plots with outliers or pseudocolor plots.

☒ A numerical value for number of cells or percentage (with statistics) is provided.

Methodology

Sample preparation	Single-cell organoid suspensions were prepared by trypsinization with Trypsin-EDTA for 5 min at 37 °C.<br><br>Single-cell tissue suspensions used for scRNA-seq were prepared by mincing punch biopsies with scissors and subjecting them to enzymatic digestion at 37 °C for 15-25 min with 1 mg/ml collagenase and 1 mg/ml Dispase II. The resulting tissue fragments were washed 3 times by means of centrifugation, resuspended in Recovery Medium and then cryo-preserved. Later, tissue fragments were thawed, washed with basal medium, and trypsinized to single cell suspensions using TrypLE supplemented with 10 µM Y-27632 for 5 min at 37 °C. To distinguish epithelial, immune and stromal cell populations and sort equal amounts of these 3 populations, single cell suspensions were stained with DRAQ7, PE anti-human CD326 (EpCAM) and FITC anti-CD45 in advanced DMEM/F12 for 30 min on ice.
Instrument	Organoids: BD FACSCelesta<br>Primary tissue for scRNA-seq: BD FACSAria III
Software	Data collection:<br>• FACS Celesta: BD FACS DIVA v9.2<br>• FACSAria III: BD FACS DIVA v.8.0.1<br><br>Data analysis:<br>BD FACS Diva v9.2 and Floreada v8/6/24
Cell population abundance	The CD45-positive and CD45-negative cell populations were subjected to scRNA-seq after sorting, which confirmed their identities as respectively immune cells and non-immune stroma. Moreover, the index sorting allowed us to confirm the expression of CD45-PE in the immune cell clusters, as shown in Extended Data Fig. 5c
Gating strategy	Organoids: SSC-A/FSC-A was used to select cells, FSC-H/FSC-A was used to select singlets. DAPI staining was used to mark dying cells. Single live cells (DAPI-) were gated in the BV421-A channel and organoid cells were separated from fibroblasts based on EpCAM-PE measured in the PE-A channel. mNeon fluorescence was measured in the FITC-A channel. Gates were set based on negative control samples.<br><br>Primary tissue for scRNA-seq: SSC-A/FSC-A was used to select cells, FSC-H/FSC-A was used to select singlets. DRAQ7 staining was used to mark dying cells, EpCam for epithelial cells, and CD45 for immune cells. Gates for all stainings were based on negative controls.

☒ Tick this box to confirm that a figure exemplifying the gating strategy is provided in the Supplementary Information.
