# Supplementary material for: Emergence of oncofetal plasticity is ubiquitous in early colorectal cancers
Source: Nature. 2026 Apr 15;654(8117):229–39. doi: 10.1038/s41586-026-10344-7 (PMC13233332; doi:10.1038/s41586-026-10344-7)
Supplement: Supplementary file 5 — Nanostring GeoMx WTA quality control report in in HTML format. [file 41586_2026_10344_MOESM5_ESM.html]

Nanostring GeoMx analysis: WTA Quality control


Code 

- Show All Code
- Hide All Code
- Download Rmd

# Nanostring GeoMx analysis: WTA Quality control

#### Remy (Sascha)

# Dataset: Human WTA

**date: 15:42 Wed 08 March, 2023**

---

Input variables

```
# input experiment folder
input_experiment <- 'exp2'

qc_assessment_date <- '2023_03_08' ### CHANGE

# save_plots <- FALSE # not used
save_tables <- TRUE
save_object <- TRUE # save probe QC Nanostring object for intermediate panel comparison
```

# 1 Loading base files

```
# Reference the main folder 'file.path' containing the sub-folders with each data file type:
base_dir <- list(
  hpc='/hpc/dlab_snippert/P0001_T1_EARLY_CANCER',
  mnt='/home/sascha/hpc/dlab_snippert/P0001_T1_EARLY_CANCER',
  umc='/home/cog/sbrunner/hpc/dlab_snippert/P0001_T1_EARLY_CANCER'
)

for(i in base_dir){
  if(dir.exists(i)){
    base_dir <- i
    break
  }
}

output_dir <- list(
  local='/home/sascha/hpc/dlab_snippert/P0001_T1_EARLY_CANCER/processed/nanostring',
  umc='/home/cog/sbrunner/hpc/dlab_snippert/P0001_T1_EARLY_CANCER/processed/nanostring'
)

for(i in output_dir){
  if(dir.exists(i)){
    output_dir <- i
    break
  }
}

plots_output_dir <- list(
  local='/home/sascha',
  umc='/home/cog/sbrunner/Documents/2022_spatial_transcriptomics/results'
)

for(i in plots_output_dir){
  if(dir.exists(i)){
    plots_output_dir <- i
    break
  }
}

# get todays date
current_date <- format(Sys.Date(), '%Y_%m_%d')
```

**loading dependencies** Please make sure the following
packages are installed and required libraries can be loaded:

- install.packages(‘devtools’)
- devtools::install\_github(‘Nanostring-Biostats/NanoStringNCTools’)
- devtools::install\_github(‘Nanostring-Biostats/GeomxTools’, ref =
  ‘dev’)
- devtools::install\_github(‘Nanostring-Biostats/GeoMxWorkflows’, ref =
  ‘main’)
- devtools::install\_github(‘DavisLaboratory/standR’)
- BiocManager::install(‘SpatialDecon’)
- BiocManager::install(‘GSVA’)
- install.packages(‘plotly’)
- install.packages(‘DT’)
- install.packages(‘msigdbr’)

```
# load libraries
source(paste0(base_dir, '/analysis/nanostring/r_objects/libs.R'))

# load color codes
source(paste0(base_dir, '/analysis/nanostring/r_objects/color_codes.R'))
ann_colors <- ann_colors_WTA
```

To locate a specific file path replace the above line with base\_dir
<- file.path(‘~/Folder/SubFolder/DataLocation’) replace the Folder,
SubFolder, DataLocation as needed. The DataLocation folder should
contain a dccs, pkcs, and annotation folder with each set of files
present as needed automatically list files in each directory for
use.

**Take care you import a column with nuclei count separately if
you want.**

```
DCCFiles <- dir(file.path(paste0(base_dir, '/processed/nanostring/', input_experiment), 'dccs'),
                pattern = '.dcc$',
                full.names = TRUE,
                recursive = TRUE)
PKCFiles <- dir(file.path(paste0(base_dir, '/processed/nanostring/', input_experiment), 'pkcs'),
                pattern = '.pkc$',
                full.names = TRUE,
                recursive = TRUE)
SampleAnnotationFile <- dir(file.path(paste0(base_dir, '/processed/nanostring/', input_experiment), 'annotation'),
                            pattern = 'final.xlsx$',
                            full.names = TRUE,
                            recursive = TRUE)
```

# 2 Load data

```
Data <- readNanoStringGeoMxSet(dccFiles = DCCFiles,
                               pkcFiles = PKCFiles,
                               # check that colnames are correct, order by Sample_ID for proper NTC calculation!
                               phenoDataFile = SampleAnnotationFile,
                               phenoDataSheet = 'Metadata', # "Metadata"
                               phenoDataDccColName = 'Sample_ID',
                               protocolDataColNames = c('aoi', 'roi'),
                               experimentDataColNames = c('panel'))
```

```
## New names:
## • `` -> `...1`
```

```
paste('Reads from following runs used: ', unique(pData(protocolData(Data))$SeqSetId))
```

```
## [1] "Reads from following runs used:  VH00208:200:AAAWKKLHV"
```

# 3 Study design

```
pkcs <- annotation(Data)
modules <- gsub('.pkc', '', pkcs)
kable(data.frame(PKCs = pkcs, modules = modules))
```

| PKCs | modules |
| --- | --- |
| Hs\_R\_NGS\_WTA\_v1.0.pkc | Hs\_R\_NGS\_WTA\_v1.0 |

Select the annotations we want to show, use `` to surround column
names with spaces or special symbols.

```
ann_selection <- list(
  annotations = c('slide name', 'Patient_ID', 'slideLocation', 'segment', 'tissueRegion'),
  annotation_labels = c('Slide name', 'Patient ID', 'Slide Location', 'Segment', 'Tissue Region')
)

# count how many ROIs per annotation
count_mat <- dplyr::count(pData(Data), across(all_of(ann_selection$annotations)))
```

Simplify the slide names if required.

```
# $'slide name' <- gsub('disease', 'd', gsub('normal', 'n', count_mat$'slide name'))
```

Gather the data and plot in order.

```
test_gr <- gather_set_data(count_mat, 1:length(ann_selection$annotations))
test_gr$x <- factor(test_gr$x, labels = ann_selection$annotations)
```

## 3.1 Plot Sankey

```
test_gr %>%
ggplot(., height = 15, width = 15, unit = 'cm', aes(x, id = id, split = y, value = n)) +
  geom_parallel_sets(aes(fill = segment), alpha = 0.5, axis.width = 0.45) +
  geom_parallel_sets_axes(aes(fill = if_else(segment == y, segment, 'grey')),
                          axis.width = 0.45, color = 'black', fill = 'grey') +
  geom_parallel_sets_labels(color = 'black', size = 5, angle = 0) +
  scale_y_continuous(expand = expansion(0)) +
  scale_x_discrete(expand = expansion(0), labels = ann_selection$annotation_labels, position = 'top') +
  labs(x = '', y = '') +
  scale_fill_manual(values = ann_colors$segment) +
  annotate(geom = 'segment', x = 4.4, xend = 4.4,
           y = 10, yend = 110, lwd = 2) +
  annotate(geom = 'text', x = 4.34, y = 50, angle = 90, size = 5,
           hjust = 0.5, label = '100 segments') +
  theme_classic(base_size = 12) +
  theme(legend.position = 'none',
        legend.title = element_blank(),
        legend.text = element_text(size = 15),
        panel.background = element_rect(fill = 'white'),
        axis.ticks.x = element_blank(),
        axis.ticks.y = element_blank(),
        axis.line = element_blank(),
        # axis.text.x = element_blank(),
        axis.text.x = element_text(size = 16, face = 'bold'),
        axis.text.y = element_blank(),
        plot.margin = margin(1,1,1,1, unit = 'cm'))
```

# 4 QC & Pre-processing

Shift counts to one

```
# shift any expression counts with a value of 0 to 1 to enable in downstream transformations.
Data <- shiftCountsOne(Data, useDALogic = TRUE)
```

## 4.1 Segment QC

We first assess sequencing quality and adequate tissue sampling for
every ROI/AOI segment. Every ROI/AOI segment will be tested for:  
 -
**Raw sequencing reads**: segments with <1000 raw reads
are removed.  
 - **% Aligned,% Trimmed, or % Stitched sequencing
reads**: Segments with reads below ~80% of the raw reads number
for one or more of these QC parameters are removed.  
 - **%
Sequencing saturation ([1 - deduplicated reads/aligned
reads]%)**: 100% sequencing saturation indicates a representative
sample, while 0% sequencing saturation indicates that all reads were
unique. Segments below ~50% require additional sequencing to capture
full sample diversity and are not typically analyzed until improved.  
- **Negative Count**: This is the geometric mean of the
several unique negative probes in the GeoMx panel that do not target any
mRNA and establish the background level of technical noise expected. It
is calculated per segment; segments with low negative counts (1-10) are
not necessarily removed but may be studied closer for low endogenous
gene signal and/or insufficient tissue sampling.  
 - **No
Template Control (NTC) count**: Values >1,000 could indicate
contamination for the segments associated with this NTC; however, in
cases where the NTC count is between 1,000-10,000, the segments may be
used if the NTC data is uniformly low (e.g. 0-2 counts for all
probes).  
 - **Nuclei**: >100 nuclei per segment is
generally recommended; however, this cutoff is highly study/tissue
dependent and may need to be reduced. What is most important is
consistency in the nuclei distribution for segments within the
study.  
 - **Area**: Generally correlates with nuclei; a
strict cutoff is not generally applied based on area.

### 4.1.1 Select Segment QC

First, we select the QC parameter cutoffs, against which our ROI/AOI
segments will be tested and flagged appropriately. We have selected the
appropriate study-specific parameters for this study. Note: the default
QC values recommended above are advised when surveying a new dataset for
the first time. Default QC cutoffs are commented in () adjacent to the
respective parameters study-specific values were selected after
visualizing the QC.  
 Results in more detail below.

```
segmentQC_params <-
  list(minsegmentReads = 1000, # Minimum number of reads (1000)
       percentTrimmed = 80,    # Minimum % of reads trimmed (80%)
       percentStitched = 80,   # Minimum % of reads stitched (80%)
       percentAligned = 80,    # Minimum % of reads aligned (80%)
       percentSaturation = 50, # Minimum sequencing saturation (50%)
       minNegativeCount = 1,   # Minimum negative control counts (10), maybe choose 2 based on the negGeoMeans
       maxNTCCount = 12000,     # Maximum counts observed in NTC well (1000)
       minNuclei = 100,         # Minimum # of nuclei estimated (100)
       minArea = 5000)         # Minimum segment area (5000)

# # set correct column names for QC flags functions
# # setNucleiFlags() expects a column 'nuclei' (lower case)
# pData(Data)$nuclei <- pData(Data)[, 'AOINucleiCount']
# pData(Data) <- subset(pData(Data), select = -c(AOINucleiCount))
# # setAreaFlags() expects a column 'area' (lower case)
# pData(Data)$area <- pData(Data)[, 'Area']

Data <- setSegmentQCFlags(Data, qcCutoffs = segmentQC_params)

segmentQCparams_df <- data.frame (
  items = c('minsegmentReads', 'percentTrimmed', 'percentStitched',
            'percentAligned', 'percentSaturation', 'minNegativeCount',
            'maxNTCCount', 'minNuclei', 'minArea'),
  defaults = c(1000, 80, 80,
               80, 50, 10,
               1000, 100, 5000),
  actual = c(segmentQC_params$minsegmentReads, segmentQC_params$percentTrimmed, segmentQC_params$percentStitched,
             segmentQC_params$percentAligned, segmentQC_params$percentSaturation, segmentQC_params$minNegativeCount,
             segmentQC_params$maxNTCCount, segmentQC_params$minNuclei, segmentQC_params$minArea)
)

datatable(segmentQCparams_df, rownames = FALSE,
          caption = 'QC thresholds',
          extensions = 'Buttons',
          options = list(
            dom = 'Bftrip',
            buttons = c('copy', 'csv', 'excel', 'pdf', 'print')
          )
)
```

### Collate QC Results

```
segmentQCResults <- protocolData(Data)[['QCFlags']]
flag_columns <- colnames(segmentQCResults)
segmentQC_Summary <- data.frame(Pass = colSums(!segmentQCResults[, flag_columns]),
                         Warning = colSums(segmentQCResults[, flag_columns]))
segmentQCResults$QCStatus <- apply(segmentQCResults, 1L, function(x) {
  ifelse(sum(x) == 0L, 'PASS', 'WARNING')
})

segmentQCResults <- segmentQCResults %>%
  mutate(nFlags = rowSums(across(where(is.logical))))

tmp_sdata <- sData(Data) %>%
  select(segment, roi, Patient_ID, tissueRegion, nuclei, `slide name`) %>%
  mutate(Replicate_ID = paste0(Patient_ID, '_', tissueRegion, '_', segment)) %>%
  mutate(Replicate_ID = factor(Replicate_ID)) %>%
  mutate(Replicate_ID_num = as.numeric(Replicate_ID))

segmentQCResults_pre <- merge(tmp_sdata, segmentQCResults, by = 0)
```

#### Segment

```
segmentQCResults_segment <- segmentQCResults_pre %>%
  group_by(segment) %>%
  summarise(across(LowReads:LowArea, sum)) %>%
  column_to_rownames(var = 'segment') %>%
  t() %>%
  bind_cols(segmentQC_Summary, .)

datatable(segmentQCResults_segment,
          caption = 'ROIs flagged per segment',
          extensions = 'Buttons',
          options = list (
            dom = 'Bftrip',
            buttons = c('copy', 'csv', 'excel', 'pdf', 'print')
          )
)
```

#### Patient

```
segmentQCResults_patient <- segmentQCResults_pre %>%
  group_by(Patient_ID) %>%
  summarise(across(LowReads:LowArea, sum)) %>%
  column_to_rownames(var = 'Patient_ID') %>%
  t() %>%
  bind_cols(segmentQC_Summary, .)

datatable(segmentQCResults_patient,
          caption = 'ROIs flagged per patient',
          extensions = 'Buttons',
          options = list (
            dom = 'Bftrip',
            buttons = c('copy', 'csv', 'excel', 'pdf', 'print')
          )
)
```

#### Region

```
segmentQCResults_region <- segmentQCResults_pre %>%
  group_by(tissueRegion) %>%
  summarise(across(LowReads:LowArea, sum)) %>%
  column_to_rownames(var = 'tissueRegion') %>%
  t() %>%
  bind_cols(segmentQC_Summary, .)

datatable(segmentQCResults_region,
          caption = 'ROIs flagged per region',
          extensions = 'Buttons',
          options = list (
            dom = 'Bftrip',
            buttons = c('copy', 'csv', 'excel', 'pdf', 'print')
          )
)
```

#### Slide name

```
segmentQCResults_slide_name <- segmentQCResults_pre %>%
  group_by(`slide name`) %>%
  summarise(across(LowReads:LowArea, sum)) %>%
  column_to_rownames(var = 'slide name') %>%
  t() %>%
  bind_cols(segmentQC_Summary, .)

datatable(segmentQCResults_slide_name,
          caption = 'ROIs flagged per slide',
          extensions = 'Buttons',
          options = list (
            dom = 'Bftrip',
            buttons = c('copy', 'csv', 'excel', 'pdf', 'print')
          )
)
```

### Replicates per ROI lost after Segment QC

```
# how many replicates per Replicate_ID do we lose?
segmentQCResults_replicates <- segmentQCResults_pre %>%
  group_by(Replicate_ID, QCStatus) %>%
  summarise(n_rep_flag = n()) %>%
  group_by(Replicate_ID) %>%
  mutate(n_rep = sum(n_rep_flag)) %>%
  ungroup() %>%
  mutate(
    PropFlagged = round((n_rep_flag / n_rep), 2),
    PropFlaggedLabel = paste0(n_rep_flag, '/', n_rep)) %>%
  mutate(
    ReplicateWarning = case_when(
      QCStatus == 'WARNING' & n_rep - n_rep_flag == 0 ~ 'NO REPLICATES LEFT',
      QCStatus == 'WARNING' & n_rep - n_rep_flag == 1 ~ '1 REPLICATES LEFT',
      TRUE ~ NA_character_
    )
  ) %>%
  select(-n_rep_flag, -n_rep) %>%
  arrange(desc(QCStatus), desc(PropFlagged), ReplicateWarning)

datatable(segmentQCResults_replicates, rownames=FALSE,
          caption = 'Number of replicates per Replicate_ID that were flagged',
          extensions = 'Buttons',
          options = list (
            dom = 'Bftrip',
            buttons = c('copy', 'csv', 'excel', 'pdf', 'print')
          )
)
```

### 4.1.2 Graphical summaries of QC statistics

```
segmentQC_Summary['TOTAL FLAGS', ] <- c(sum(segmentQCResults[, 'QCStatus'] == 'PASS'),
                                 sum(segmentQCResults[, 'QCStatus'] == 'WARNING'))

# column to fill plots by
col_by <- 'segment'

QC_histogram <- function(assay_data = NULL,
                         annotation = NULL,
                         fill_by = NULL,
                         thr = NULL,
                         scale_trans = NULL) {
  plt <- ggplot(assay_data,
                aes_string(
                  x = paste0('unlist(`', annotation, '`)'),
                  fill = fill_by)
                ) +
    geom_histogram(bins = 100) +
    geom_vline(
      xintercept = thr,
      lty = 'dashed',
      color = 'black'
      ) +
    theme_bw() +
    guides(fill = 'none') +
    facet_wrap(
      as.formula(paste('~', fill_by)),
      nrow = 4,
      scales = 'free_y'
      ) +
    scale_fill_manual(values = ann_colors[[fill_by]]) +
    labs(
      title = annotation,
      x = annotation,
      y = '# segments'
      )
  if(!is.null(scale_trans)) {
    plt <- plt +
      scale_x_continuous(trans = scale_trans)
  }
  plt
}
```

#### Trimmed

```
QC_histogram(sData(Data), 'Trimmed (%)', col_by, segmentQC_params$percentTrimmed) +
  scale_x_continuous(
    limits = c(80,100),
    breaks = c(85, 90, 95, 100, segmentQC_params$percentTrimmed)
  )
```

#### Stiched (%)

```
QC_histogram(sData(Data), 'Stitched (%)', col_by, segmentQC_params$percentStitched) +
  scale_x_continuous(
    limits = c(0,100),
    breaks = c(0, 25, 50, 75, 100, segmentQC_params$percentStitched)
  )
```

#### Aligned (%)

```
QC_histogram(sData(Data), 'Aligned (%)', col_by,segmentQC_params$percentAligned) +
  scale_x_continuous(
    limits = c(0,100),
    breaks = c(0, 25, 50, 75, 100, segmentQC_params$percentAligned)
  )
```

#### Sequencing Saturation (%)

```
QC_histogram(sData(Data), 'Saturated (%)', col_by, segmentQC_params$percentSaturation) +
  labs(
    title = 'Sequencing Saturation (%)',
    x = 'Sequencing Saturation (%)'
    ) +
  scale_x_continuous(
    limits = c(0,100),
    breaks = c(0, 25, 50, 75, 100, segmentQC_params$percentSaturation)
  )
```

#### Area

```
QC_histogram(sData(Data), 'area', col_by, segmentQC_params$minArea, scale_trans = 'log10') +
    labs(
    title = 'Area',
    x = 'Area'
    ) +
  scale_x_continuous(
    limits = c(1000,200000),
    breaks = c(1000, 10000, 100000, segmentQC_params$minArea),
    trans = 'log10'
  )
```

```
## Scale for x is already present.
## Adding another scale for x, which will replace the existing scale.
```

#### Nuclei count

```
QC_histogram(sData(Data), 'nuclei', col_by, segmentQC_params$minNuclei) +
    labs(
    title = '# Nuclei',
    x = '# Nuclei'
    ) +
  scale_x_continuous(
    limits = c(0,1500),
    breaks = c(0, 250, 500, 750, 1000, 1250, 1500, segmentQC_params$minNuclei)
  )
```

#### DuplicationRate: Dedup / Raw

```
ggplot(pData(protocolData(Data)),
       aes(
         x = Plate_ID,
         y = (DeduplicatedReads/Raw))
         ) +
  geom_violin(aes(fill = Plate_ID)) +
  geom_jitter(width = 0.2) +
  labs(y = 'Deduplicated / Raw reads') +
  scale_y_continuous(labels = scales::percent) +
  theme_bw() +
    theme(
    axis.title.x = element_blank(),
    axis.text.x = element_blank(),
    axis.ticks.x = element_blank()
  )
```

#### DeuplicationRate: Dedup / Aligned

```
ggplot(pData(protocolData(Data)),
       aes(
         x = Plate_ID,
         y = (DeduplicatedReads/Aligned))
         ) +
  geom_violin(aes(fill = Plate_ID)) +
  geom_jitter(width = 0.2) +
  labs(y = 'Deduplicated / Aligned reads') +
  scale_y_continuous(labels = scales::percent) +
  theme_bw() +
  theme(
    axis.title.x = element_blank(),
    axis.text.x = element_blank(),
    axis.ticks.x = element_blank()
  )
```

#### Negprobes vs Endogenous

```
tmp_target_Data <- aggregateCounts(Data)

# get negative probe data
tmp_negs <- subset(tmp_target_Data, CodeClass == 'Negative')

p1 <- ggplot(pData(tmp_negs),
             aes(
               x = segment,
               y = assayDataElement(tmp_negs, elt = 'exprs'))) +
  geom_violin(aes(fill = segment)) +
  geom_jitter(width = 0.2) +
  coord_flip() +
  labs(y = 'Negative probes expression') +
  scale_fill_manual(values = ann_colors$segment) +
  scale_y_continuous(limits = c(1,3000), trans = 'log2') +
  theme_bw()

# get endogenous probe data
tmp_end <- subset(tmp_target_Data, CodeClass == 'Endogenous')

p2 <- ggplot(pData(tmp_end),
             aes(
               x = segment,
               y = colMeans(assayDataElement(tmp_end, elt = 'exprs')))) +
  geom_violin(aes(fill = segment)) +
  geom_jitter(width = 0.2) +
  coord_flip() +
  labs(y = 'Endogenous probes expression (mean)') +
  scale_fill_manual(values = ann_colors$segment) +
  scale_y_continuous(limits = c(1,3000), trans = 'log2') +
  theme_bw()

pl <- list(p1, p2)

plot_grid(plotlist = pl, nrow = 2, align = 'v')
```

#### Neg\_probe reads compared to raw\_reads

```
# make background total neg probe count
tmp_fdata_df <- fData(Data)
negprobesnames <- rownames(tmp_fdata_df[tmp_fdata_df$Negative == TRUE,])
tmp_exp <- assayDataElement(Data, elt = 'exprs')
negprobe_expr_fd <- tmp_exp[rownames(tmp_exp) %in% negprobesnames, ]
tot_neg_ctrl_reads <- colSums(negprobe_expr_fd)
tot_dedup_reads <- pData(protocolData(Data))$DeduplicatedReads

tmp_negdedupreads_df <- data.frame('aoi' = names(tot_neg_ctrl_reads),
                                   'tot_dedup_reads' = as.numeric(tot_dedup_reads),
                                   'tot_neg_ctrl_reads' = as.numeric(tot_neg_ctrl_reads)
)

tmp_negdedupreads_df <- melt(tmp_negdedupreads_df, id = 'aoi')

ggplot(tmp_negdedupreads_df,
       aes(
         x = aoi,
         y = value,
         fill = variable)) +
  geom_bar(position = 'identity', stat = 'identity') +
  scale_y_continuous(trans = log2_trans()) +
  labs(
    x = 'AOI',
    y = 'Number of reads',
    fill = ''
    ) +
  theme(
    legend.position = 'bottom',
    axis.text.x = element_blank(),
    axis.ticks.x = element_blank()
    )
```

#### Duplicated reads vs Background

```
# get dcc per plate. sum negprobe counts/dcc/plate
ggplot(pData(protocolData(Data)),
       aes(
         x = Plate_ID,
         y = DeduplicatedReads,
         fill = Plate_ID)) +
  geom_violin() +
  geom_jitter(width = 0.2) +
  labs(y = 'Deduplicated / Raw reads') +
  scale_y_log10() +
  geom_hline(data = pData(protocolData(Data)),
             aes(
               yintercept = NTC,
               colour = Plate_ID
               )) +
  theme_bw()
```

#### Duplicated reads vs ROIarea

```
tmp_df <- cbind(pData(Data),
                 pData(protocolData(Data)),
                 dcc=rownames(pData(Data)))

ggplot(tmp_df %>% filter((DeduplicatedReads/area) <= 5),
       aes(
         x = dcc,
         y = (DeduplicatedReads/area),
         colour =`slide name`)) +
  geom_point(size = 4, position = position_dodge(width = 1)) +
  scale_x_discrete(expand = expand_scale(add = c(1,1)))+
  scale_y_continuous(limits = c(0,5)) +
  labs(y = 'Deduplicated reads / ROI area') +
  theme(
    plot.margin = unit(c(1,1,1,6), 'cm'),
    axis.text = element_text(size = 6),
    axis.text.x = element_text(angle = 45, hjust = 1)
    )
```

#### Duplicated reads vs nuclei

```
tmp_df <- cbind(pData(Data),
                 pData(protocolData(Data)),
                 dcc=rownames(pData(Data)))

ggplot(tmp_df %>% filter((DeduplicatedReads/nuclei) < 3000),
       aes(
         x = dcc,
         y = (DeduplicatedReads/nuclei),
         colour = `slide name`
         )) +
  geom_point(size = 4, position = position_dodge(width = 1)) +
  scale_x_discrete(expand = expand_scale(add = c(1,1)))+
  scale_y_continuous(limits = c(0,3000)) +
  labs(y = 'Deduplicated reads / nuclei') +
  theme(
    plot.margin = unit(c(1,1,1,2), 'cm'),
    axis.text = element_text(size = 6),
    axis.text.x = element_text(angle = 45, hjust = 1)
    )
```

### 4.1.3 Process Negative GeoMeans

```
# calculate the negative geometric means for each module
negativeGeoMeans <- esBy(negativeControlSubset(Data),
                         GROUP = 'Module',
                         FUN = function(x) {
                           assayDataApply(x, MARGIN = 2, FUN = ngeoMean, elt = 'exprs')
                           })
protocolData(Data)[['NegGeoMean']] <- negativeGeoMeans
negCols <- paste0('NegGeoMean_', modules)
pData(Data)[, negCols] <- sData(Data)[['NegGeoMean']]

for(ann in negCols) {
  plt <- QC_histogram(pData(Data), ann, col_by, 10, scale_trans = 'log10')
  print(plt)
}
```

```
# Detatch neg_geomean columns ahead of aggregateCounts call
pData(Data) <- pData(Data)[, !colnames(pData(Data)) %in% negCols]
```

Show all NTC values, Freq = # of segments with a given NTC count:

```
tmp_segmentQC <- sData(Data)

# calculate how many flags a ROI received
tmp_segmentQC <- tmp_segmentQC %>%
  mutate(
    QCStatus = if_else(rowSums(across(QCFlags)) == 0L, 'PASS', 'WARNING'),
    nFlags = rowSums(across(QCFlags)), .after = QCFlags
    )

tmp_ntc_df <- tmp_segmentQC[,c('slide name', 'Plate_ID', 'NTC_ID', 'NTC')]
tmp_ntc_df <- tmp_ntc_df %>% dplyr::count(tmp_ntc_df$'slide name', tmp_ntc_df$NTC_ID, tmp_ntc_df$Plate_ID, tmp_ntc_df$NTC)
colnames(tmp_ntc_df) <- c('Slide name', 'NTC_ID', 'Plate_ID',
                         'NTC count', 'Number of samples')

datatable(tmp_ntc_df, rownames = FALSE)
```

```
datatable(segmentQC_Summary,
          caption = 'AOI QC Summary',
          extensions = 'Buttons',
          options = list (
            dom = 'Bftrip',
            buttons = c('copy', 'csv', 'excel', 'pdf', 'print')
          )
)
```

### AOIs that fail QC

Show AOIs which fail critical QCs.

```
qc_col_list <- as.list(colnames(tmp_segmentQC))
names(qc_col_list) <- colnames(tmp_segmentQC)
failed_qc_segments_all <- tmp_segmentQC[tmp_segmentQC[qc_col_list$QCStatus] == 'WARNING',] # all flagged segments

# pull nested QCFlags df out of object (it will mess up the datatable otherwise); tidy the table
failed_qc_segments_all1 <- cbind(failed_qc_segments_all, failed_qc_segments_all %>% pull(QCFlags)) %>%
  # remove the nested dfs, they interfere with the generation of a TSV
  select(-c(`Trimmed (%)`:NegGeoMean), -QCFlags)

# pull nested QC percentages and NegGeoMean nested dfs and bring them to the same level as the other columns to write the table to disk
failed_qc_segments_all1 <- cbind(failed_qc_segments_all1,
                                 failed_qc_segments_all %>% pull(`Trimmed (%)`),
                                 failed_qc_segments_all %>% pull(`Stitched (%)`),
                                 failed_qc_segments_all %>% pull(`Aligned (%)`),
                                 failed_qc_segments_all %>% pull(`Saturated (%)`),
                                 failed_qc_segments_all %>% pull(NegGeoMean)) %>%
  select(SampleID, `slide name`, Patient_ID, roi, segment, slideLocation, tissueRegion,
         QCStatus, nFlags, area, nuclei, umiQ30, rtsQ30,
         Raw:Aligned, DeduplicatedReads, `Trimmed (%)`:Aligned, LowReads:LowArea, NTC_ID, NTC) %>%
  # rename for better readability
  rename(
    SampleID = 'Sample_ID'
  ) %>%
  arrange(`slide name`, Patient_ID, roi)

# write this table to disk
qc_dir <- paste0(output_dir, '/', input_experiment, '/output/qc_results/', current_date)
if (!dir.exists(qc_dir)) {
  dir.create(qc_dir, recursive = TRUE)
}
write_tsv(
  failed_qc_segments_all1,
  file = paste0(qc_dir, '/failed_qc_segments.tsv')
) 

# also print the table
datatable(failed_qc_segments_all1,
          rownames = FALSE,
          extensions = 'Buttons', options = list (
            dom = 'Bftrip',
            buttons = c('copy', 'csv', 'excel', 'pdf', 'print')
          )
)
```

There are 4 segments which failed at least one QC measure. This table
was assessed manually for whether segments were still acceptable to be
included in downstream analysis. Manually assessed segments were put
into one of three categories:  
 - **INCLUDE:** segment
showed enough quality to be included even after flagging. There could be
several reasons for this. For example, it scored just below the
threshold for one parameter, but had good qualities overall. Or, it was
flagged due to QC measures which are largely study dependent and have a
lot of leeway (e.g. number of nuclei).  
 - **CONSIDER:**
segment showed didn’t show enough quality to be included, but is also
not of worse enough quality to be completely removed. The reasons for
this are different and mostly the interplay of mutliple quality measures
were considered for this decision. It could either be that one quality
measure was subpar, but others were amazing. Or, multiple quality
measures were found to be just below threshold. This category provides
potential segments that could be included further downstream, but have
to be monitored closely.  
 - **REMOVE:** The segment
showed very bad quality for one or even multiple QC measures.  
 Every
decision is accompanied with a comment that can be found in the column
*AssessmentComment*. Read the manually assessed segment QC table
into R again.  
 After manual assessment of the segments that were
flagged as subpar according to the default QC parameters, onlt those
that were flagged **INCLUDED** were included.

```
# failed_qc_assessed_file <- paste0(output_dir, '/', input_experiment, '/output/qc_results/', qc_assessment_date, '/failed_qc_segments_assessed.csv')
```

Subsetting our dataset: remove samples which did not pass QC.

```
# Data <- Data[, segmentQCResults$ManualAssessment %in% c('INCLUDE')]
Data <- Data[, segmentQCResults$QCStatus == 'PASS']
# dim(Data)
```

## 4.2 Probe QC

Generally keep the qcCutoffs parameters for probe QC unchanged. Set
*removeLocalOutliers* to `FALSE` if you do not want to
remove local outliers. To check which local outliers have been flagged,
use `exprs(Data)`; the outliers are marked `NA`
and are removed when aggregating the probe counts per target withing the
`aggregateCounts(Data)` function downstream via the
`na.rm = TRUE` parameter.

```
Data <- setBioProbeQCFlags(Data,
                           qcCutoffs = list(minProbeRatio = 0.1,
                                            percentFailGrubbs = 20),
                           removeLocalOutliers = TRUE)
ProbeQCResults <- fData(Data)[['QCFlags']]

probeQC_tmp <- ProbeQCResults %>%
  select(GlobalGrubbsOutlier) %>%
  rownames_to_column(var = 'RTS_ID')

probeQC_tmp2 <- probeQC_tmp %>%
  inner_join(fData(Data), by = c('RTS_ID'))

# how many probes per gene target?
mean_number_of_probes_per_gene <- probeQC_tmp2 %>%
  group_by(TargetName) %>%
  dplyr::count() %>%
  ungroup() %>%
  summarise(mean = mean(n)) %>%
  pull()

ProbeQCResults$QCStatus <- apply(ProbeQCResults, 1L, function(x) {
  ifelse(sum(x) == 0L, 'PASS', 'WARNING')
})

Local_outliers <- ProbeQCResults %>%
  select(starts_with('LocalGrubbsOutlier'))
nLocal_outliers <- colSums(Local_outliers)
nLocal_outliers <- sort(nLocal_outliers, decreasing = T)
Local_outliers$nSegmentOutlier <- apply(Local_outliers, 1L, function(x) {
  sum(x)
})

# sort
Local_outliers <- Local_outliers %>%
  arrange(desc(nSegmentOutlier))

total_local_outliers <- sum(Local_outliers$nSegmentOutlier)

nProbes_with_double_digit_segment_outliers <- Local_outliers %>%
  filter(nSegmentOutlier > 9) %>%
  summarise(sum = n()) %>% pull()

### OPTIONAL: write Probe QC nanostring object to disk to compare CTA & WTA probes
if (save_object) {
  saveRDS(
    object = Data,
    file = paste0(output_dir, '/', input_experiment, '/output/qc_results/', qc_assessment_date, '/probeqc_Data.RDS')
  )
}
```

There are 18815 probes tested with an average of of 1.01 probes per
gene target in `featureData`. A total of 66 local outliers
out of 5287015 (0%) possible outliers were identified according to the
Grubb’s test. The segment with the highest number of local outliers (1)
was DSP-1001660016773-G-A09. 1 probes were found to be local outliers in
at least 10 segments. The most faulty probe was RTS0039347 which was
found to be an outlier in 4% of segments. Mean and median number of
outlier segments per probe were 0 and 0, respectively.  
 EDIT: After
assessing how many probes were flagged as local outliers, we decided to
exclude them, because we believe that we don’t lose too much
information.

Define QC table for Probe QC

```
rm(Local_outliers)
probeQC_df <- data.frame(Passed = sum(rowSums(ProbeQCResults[, grepl('GrubbsOutlier', colnames(ProbeQCResults))]) == 0),
                    Global = sum(ProbeQCResults$GlobalGrubbsOutlier),
                    Local = sum(rowSums(ProbeQCResults[, grepl('LocalGrubbsOutlier', colnames(ProbeQCResults))]) > 0
                                & !ProbeQCResults$GlobalGrubbsOutlier))
```

Subset object to exclude all that did not pass probe ratio &
global testing.

```
ProbeQCPassed <- subset(Data,
                        fData(Data)[['QCFlags']][, c('LowProbeRatio')] == FALSE &
                          fData(Data)[['QCFlags']][, c('GlobalGrubbsOutlier')] == FALSE)
dim(ProbeQCPassed)
```

```
## Features  Samples 
##    18815      281
```

```
Data <- ProbeQCPassed
```

Check how many unique targets (i.e. genes) the object has.

```
length(unique(featureData(Data)[['TargetName']]))
```

```
## [1] 18677
```

### Collapse to targets

The probes are aggregated per target by using the geometric mean of
all target probes in all segments by default.  
 The function also
calculates the NegGeoMean and NegGeoSD for each segment & module and
appends this information to `pData(Data)`.

```
target_Data <- aggregateCounts(Data)
dim(target_Data)
```

```
## Features  Samples 
##    18677      281
```

```
exprs(target_Data)[1:5, 1:2]
```

```
##       DSP-1001660016773-G-A02.dcc DSP-1001660016773-G-A03.dcc
## A2M                           233                          76
## NAT2                           22                          28
## ACADM                          11                          60
## ACADS                          22                          86
## ACAT1                          23                          92
```

## 4.3 Background quantification QC

### 4.3.1 Limit of Quantification

We define a limit of quantification (LOQ) per ROI/AOI segment based
on the negative control probes to guide the filtering of segments and
genes with low signal relative to background. The formula for
calculating the LOQ in the \(i^{th}\)
segment at \(n\) standard deviations
  
 (\(n = 2\) for this study) is:
\(LOQ\_i=geomean(NegProbe\_i)\*geoSD(NegProbe\_i)^n\)

Define LOQ SD threshold and minimum value

```
LOQSD_cutoff <- 0.5
minLOQ <- 2
```

Calculate LOQ per module tested

```
LOQ <- data.frame(row.names = colnames(target_Data))

for(module in modules) {
  vars <- paste0(c('NegGeoMean_', 'NegGeoSD_'),
                 module)
  if(all(vars[1:2] %in% colnames(pData(target_Data)))) {
    LOQ[, module] <- pmax(minLOQ,
                          pData(target_Data)[, vars[1]] * pData(target_Data)[, vars[2]] ^ LOQSD_cutoff
                          )
  }
}

pData(target_Data)$LOQ <- LOQ
```

### 4.3.2 Low negative background filtering

After determining the limit of quantification (LOQ) per segment, we
recommend filtering out either segments and/or genes with abnormally low
signal. Filtering is an important step to focus on the true biological
data of interest. We determine the number of genes detected in each
segment across the dataset.

```
LOQ_Mat <- c()
for(module in modules) {
  ind <- fData(target_Data)$Module == module
  Mat_i <- t(esApply(target_Data[ind, ], MARGIN = 1,
                     FUN = function(x) {
                       x > LOQ[, module]
                     }))
  LOQ_Mat <- rbind(LOQ_Mat, Mat_i)
}

# ensure ordering since this is stored outside of the geomxSet
LOQ_Mat <- LOQ_Mat[fData(target_Data)$TargetName, ]
```

### 4.3.3 Segment Gene Detection

We first filter out segments with exceptionally low signal. These
segments will have a small fraction of panel genes detected above the
LOQ relative to the other segments in the study. Let’s visualize the
distribution of segments with respect to their % genes detected:

Save detection rate information to pheno data

```
pData(target_Data)$GenesDetected <- colSums(LOQ_Mat, na.rm = TRUE)
pData(target_Data)$GeneDetectionRate <- pData(target_Data)$GenesDetected / nrow(target_Data)
```

Determine detection thresholds: 1%, 5%, 10%, 15%, >15%

```
pData(target_Data)$DetectionThreshold <- cut(pData(target_Data)$GeneDetectionRate,
                                             breaks = c(0, 0.01, 0.05, 0.1, 0.15, 0.2, 1),
                                             labels = c('<1%', '1-5%', '5-10%', '10-15%', '15-20%', '>20%'))
```

```
# column to fill plots by
col_by <- 'segment'

# gene detection rate plotting function
gene_det_rate_barplot <- function(assay_data = NULL,
                         annotation = NULL,
                         fill_by = NULL) {
  plt <- ggplot(assay_data,
                aes(x = DetectionThreshold)) +
  geom_bar(aes_string(fill = paste0('unlist(`', fill_by, '`)'))) +
  geom_text(stat = 'count', aes(label = ..count..), vjust = -0.5) +
  theme_bw() +
  scale_y_continuous(expand = expansion(mult = c(0, 0.1))) +
  scale_fill_manual(values = ann_colors[[fill_by]]) +
  labs(x = 'Gene Detection Rate',
       y = 'segments, #',
       fill = annotation)
  
  return(plt)
}
```

#### By Segment Type

```
# stacked bar plot of different cut points (1%, 5%, 10%, 15%)
gene_det_rate_barplot(pData(target_Data), annotation = 'Segment', fill_by = 'segment')
```

```
kable(table(pData(target_Data)$DetectionThreshold,
            pData(target_Data)$segment))
```

|  | PanCK- | PanCK+ |
| --- | --- | --- |
| <1% | 0 | 0 |
| 1-5% | 0 | 0 |
| 5-10% | 0 | 0 |
| 10-15% | 0 | 0 |
| 15-20% | 0 | 0 |
| >20% | 138 | 143 |

#### By Region

```
# stacked bar plot of different cut points (1%, 5%, 10%, 15%)
gene_det_rate_barplot(pData(target_Data), annotation = 'Tissue region', fill_by = 'tissueRegion')
```

```
kable(table(pData(target_Data)$DetectionThreshold,
            pData(target_Data)$tissueRegion))
```

|  | ADE | BONUS | CORE | INV | NOR |
| --- | --- | --- | --- | --- | --- |
| <1% | 0 | 0 | 0 | 0 | 0 |
| 1-5% | 0 | 0 | 0 | 0 | 0 |
| 5-10% | 0 | 0 | 0 | 0 | 0 |
| 10-15% | 0 | 0 | 0 | 0 | 0 |
| 15-20% | 0 | 0 | 0 | 0 | 0 |
| >20% | 42 | 2 | 92 | 96 | 49 |

#### By Patient

```
# stacked bar plot of different cut points (1%, 5%, 10%, 15%)
gene_det_rate_barplot(pData(target_Data), annotation = 'Patient ID', fill_by = 'Patient_ID')
```

```
kable(table(pData(target_Data)$DetectionThreshold,
            pData(target_Data)$Patient_ID))
```

|  | T1\_NANO\_011 | T1\_NANO\_012 | T1\_NANO\_013 | T1\_NANO\_014 | T1\_NANO\_015 | T1\_NANO\_016 | T1\_NANO\_017 | T1\_NANO\_018 | T1\_NANO\_019 | T1\_NANO\_020 |
| --- | --- | --- | --- | --- | --- | --- | --- | --- | --- | --- |
| <1% | 0 | 0 | 0 | 0 | 0 | 0 | 0 | 0 | 0 | 0 |
| 1-5% | 0 | 0 | 0 | 0 | 0 | 0 | 0 | 0 | 0 | 0 |
| 5-10% | 0 | 0 | 0 | 0 | 0 | 0 | 0 | 0 | 0 | 0 |
| 10-15% | 0 | 0 | 0 | 0 | 0 | 0 | 0 | 0 | 0 | 0 |
| 15-20% | 0 | 0 | 0 | 0 | 0 | 0 | 0 | 0 | 0 | 0 |
| >20% | 24 | 31 | 34 | 34 | 16 | 25 | 21 | 32 | 30 | 34 |

#### By Slide

```
# stacked bar plot of different cut points (1%, 5%, 10%, 15%)
gene_det_rate_barplot(pData(target_Data), annotation = 'Slide', fill_by = 'slide name')
```

```
kable(table(pData(target_Data)$DetectionThreshold,
            pData(target_Data)$`slide name`))
```

|  | slide1 | slide2 | slide3 | slide4 | slide5 |
| --- | --- | --- | --- | --- | --- |
| <1% | 0 | 0 | 0 | 0 | 0 |
| 1-5% | 0 | 0 | 0 | 0 | 0 |
| 5-10% | 0 | 0 | 0 | 0 | 0 |
| 10-15% | 0 | 0 | 0 | 0 | 0 |
| 15-20% | 0 | 0 | 0 | 0 | 0 |
| >20% | 55 | 68 | 41 | 53 | 64 |

### Gene detection rate visualized

```
# set threshold for detectionlevel
# default 0.1
default_gene_det_threshold <- 0.1
gene_det_threshold <- 0.05

# calc median gene detection rate
median_gene_detection_rate <- median(pData(target_Data)$GeneDetectionRate)

# visualize gene detection rate as density histogram 
ggplot(pData(target_Data),
       aes(
         x = GeneDetectionRate
       )) +
  geom_histogram(
    aes(
      y = ..density..
      ),
    bins = 50,
    color = 'black',
    fill = 'white'
    ) +
  geom_density(
    color = 'steelblue',
    fill = 'steelblue',
    alpha = 0.3
    ) +
  geom_vline(
    aes(
      xintercept = median_gene_detection_rate
    ),
    color = '#6C7B8B',
    lwd = 2
  ) +
  geom_vline(
    aes(
      xintercept = default_gene_det_threshold
    ),
    color = '#1C86EE',
    lwd = 2
  ) +
  geom_vline(
    aes(
      xintercept = gene_det_threshold
    ),
    color = '#104E8B',
    lwd = 2
  ) +
  scale_x_continuous(
    limits = c(-0.1,1),
    breaks = round(c(seq(0, 1, by = 0.25), median_gene_detection_rate), 2)
  )
```

Grey thick vertical line denotes the median gene detection rate
across all segments/ROIs.  
 Lightblue thick vertical line denotes the
default gene detection threshold. Darkblue thick vertical line denotes
the chosen gene detection threshold after examining the data.

### Filter out segments with abnormally low signal

```
# filter out segments with abnormally low signal
target_Data <- target_Data[, pData(target_Data)$GeneDetectionRate >= gene_det_threshold]

dim(target_Data)
```

```
## Features  Samples 
##    18677      281
```

```
# calculate how many segments are lost due to gene detection threshold
nSamples_prefilter_LOQ <- unname(ncol(Data))
nSample_postfilter_LOQ <- unname(ncol(target_Data))
nSamples_lost_LOQ <- nSamples_prefilter_LOQ - nSample_postfilter_LOQ
```

LOQ filtering thresholds were:  
 1. minimum LOQ of 2 (default:
2).  
 2. minimum NegGeoSD ^ 0.5 (default \(NegGeoSD^2\)) standard deviations per
segment.  
 3. minimum gene detection rate of 0.05 (default:
0.1).  
  
 After filtering the segments by gene detection rate based
on LOQ, there were 0 samples lost (prefilter: 281, postfilter: 281).

## 4.4 collect annotations

```
# **Select the annotations we want to show, use `` to surround column names with spaces or special symbols**
count_mat <- dplyr::count(pData(Data), segment, slideLocation , tissueRegion, Patient_ID, `slide name`)

# gather the data and plot in order: class, slide name, region, segment
test_gr <- gather_set_data(count_mat, 1:2)
test_gr$x <- factor(test_gr$x,
                    levels = c('slide name', 'segment'))

aoilist <-names(as.data.frame(assayDataElement(target_Data, elt = 'exprs')))

segment <-as.data.frame(pData(target_Data)$segment, unique(count_mat$segment))
colnames(segment) <- 'segment'
row.names(segment) <- aoilist

slideLocation <- as.data.frame(pData(target_Data)$slideLocation, unique(count_mat$slideLocation))
colnames(slideLocation) <- 'Location'
row.names(slideLocation) <- aoilist

tissueRegion <- as.data.frame(pData(target_Data)$tissueRegion, unique(count_mat$tissueRegion))
colnames(tissueRegion) <- 'Structure'
row.names(tissueRegion) <- aoilist

Patient_ID <- as.data.frame(pData(target_Data)$Patient_ID, unique(count_mat$Patient_ID))
colnames(Patient_ID) <- 'id'
row.names(Patient_ID) <- aoilist

SN <- as.data.frame(pData(target_Data)$`slide name`, unique(count_mat$`slide name`))
colnames(SN) <- 'slide name'
row.names(SN) <- aoilist

ann <- cbind(segment, slideLocation, tissueRegion, Patient_ID, SN)
```

## 4.5 Manual removal of samples/classes

```
# active_aois <- rownames(ann)[ann$status! = 'Inflamed']

# target_Data <-  target_Data[, active_aois]

# dim(target_Data)

# count_mat <- count_mat[count_mat$segment != 'Inflamed',]
```

re-Collect annotations

```
# gather the data and plot in order: slide name, segment, slideLocation tissueRegion, Patient_ID
count_mat_filtered <- dplyr::count(pData(target_Data), `slide name`, segment, slideLocation, tissueRegion, Patient_ID)
test_gr_filtered <- gather_set_data(count_mat_filtered, 1:5)
test_gr_filtered$x <- factor(test_gr_filtered$x,
                             labels = c('slide name', 'Patient_ID', 'slideLocation', 'segment', 'tissueRegion'))
```

re-Plot Sankey

```
test_gr_filtered %>%
  ggplot(., height = 10, width = 10, aes(x, id = id, split = y, value = n)) +
  geom_parallel_sets(aes(fill = segment), alpha = 0.5, axis.width = 0.45) +
  geom_parallel_sets_axes(aes(fill = if_else(segment == y, segment, 'grey')),
                          axis.width = 0.45, color = 'black', fill = 'grey') +
  geom_parallel_sets_labels(color = 'black', size = 5, angle = 0) +
  scale_y_continuous(expand = expansion(0)) +
  scale_x_discrete(expand = expansion(0), labels = ann_selection$annotation_labels, position = 'top') +
  labs(x = '', y = '') +
  scale_fill_manual(values = ann_colors$segment) +
  annotate(geom = 'segment', x = 4.4, xend = 4.4,
           y = 10, yend = 110, lwd = 2) +
  annotate(geom = 'text', x = 4.34, y = 50, angle = 90, size = 5,
           hjust = 0.5, label = '100 segments') +
  theme_classic(base_size = 12) +
  theme(legend.position = 'none',
        legend.title = element_blank(),
        legend.text = element_text(size = 15),
        panel.background = element_rect(fill = 'white'),
        axis.ticks.x = element_blank(),
        axis.ticks.y = element_blank(),
        axis.line = element_blank(),
        # axis.text.x = element_blank(),
        axis.text.x = element_text(size = 16, face = 'bold'),
        axis.text.y = element_blank(),
        plot.margin = margin(1,1,1,1, unit = 'cm'))
```

## 4.6 Gene Level QC

### Calculate gene detection rate

```
LOQ_Mat <- LOQ_Mat[, colnames(target_Data)]

fData(target_Data)$DetectedSegments <- rowSums(LOQ_Mat, na.rm = TRUE)
fData(target_Data)$DetectionRate <- fData(target_Data)$DetectedSegments / nrow(pData(target_Data))
```

Check which genes are failing to be detected across different
annotation features

Gene of interest detection table:

```
# Gene of interest detection table
goi <- c('ACTA2', 'PDGFRA', 'PDGFRB', 'IL6', 'FAP', 'TNF','CXCL14', 'SFRP1', 'RSPO3', 'GREM1', 'EMP1', 'LGR5',
                   'SFRP2', 'SFRP4', 'TGFB1', 'TGFB3', 'GLI1', 'BMP4', 'MMP7', 'MMP1', 'MMP3', 'RSPO1', 'RSPO2', 'RSPO4')
goi_df <- data.frame(
    Gene = goi,
    Number = fData(target_Data)[goi, "DetectedSegments"],
    DetectionRate = percent(fData(target_Data)[goi, "DetectionRate"])) %>%
  arrange(Gene)

# write to disk
if (save_tables) {
  write_tsv(
    goi_df,
    file = paste0(output_dir, '/', input_experiment, '/output/qc_results/', current_date, '/goi_detection_rate.tsv')
  )
}
```

### Gene Filtering

We will graph the total number of genes detected in different
percentages of segments. Based on the visualization below, we can better
understand global gene detection in our study and select how many low
detected genes to filter out of the dataset. Gene filtering increases
performance of downstream statistical tests and improves interpretation
of true biological signal.

Plot detection rate

```
plot_detect <- data.frame(Freq = c(0,1, 2, 5, 10, 20, 30, 50, 75, 90, 100))
plot_detect$Number <- unlist(lapply(c(0,0.01, 0.02, 0.05, 0.1, 0.2, 0.3, 0.5, 0.75, 0.9, 1),
                                    function(x) {
                                      sum(fData(target_Data)$DetectionRate >= x)
                                      }))
plot_detect$Rate <- plot_detect$Number / nrow(fData(target_Data))
rownames(plot_detect) <- plot_detect$Freq

ggplot(plot_detect,
       aes(
         x = as.factor(Freq),
         y = Rate,
         fill = Rate)) +
  geom_bar(stat = 'identity') +
  geom_text(
    aes(label = formatC(Number, format = 'd', big.mark = ',')),
    vjust = 1.6,
    color = 'black',
    size = 4) +
  scale_fill_gradient2(
    low = 'orange2',
    mid = 'lightblue',
    high = 'dodgerblue3',
    midpoint = 0.65,
    limits = c(0,1),
    labels = scales::percent) +
  theme_bw() +
  scale_y_continuous(
    labels = scales::percent,
    limits = c(0,1),
    expand = expansion(mult = c(0, 0))) +
  labs(x = '% of segments',
       y = 'Genes Detected, % of Panel > LOQ')
```

Subset to target genes detected in at least 5% of the samples
(default: >=10% of segments). Also manually include the negative
control probe, for downstream use.

```
# calculate how many segments are lost due to gene detection threshold
nGenes_prefilter_LOQ <- unname(nrow(target_Data))

# filter out genes that are found in less than N % of segments due to below-LOQ expression
# default = 0.1 (>=10% of segments)
negativeProbefData <- subset(fData(target_Data), CodeClass == 'Negative')
neg_probes <- unique(negativeProbefData$TargetName)
target_Data <- target_Data[fData(target_Data)$DetectionRate >= gene_det_threshold | fData(target_Data)$TargetName %in% neg_probes, ]

# calculate how many segments are lost due to gene detection threshold
nGenes_postfilter_LOQ <- unname(nrow(target_Data))
nGenes_lost_LOQ <- nGenes_prefilter_LOQ - nGenes_postfilter_LOQ

dim(target_Data)
```

```
## Features  Samples 
##    18441      281
```

```
# retain only detected genes of interest
# goi <- goi[goi %in% rownames(target_Data)]
```

After filtering the genes by gene detection rate based on LOQ, there
were 236 genes lost (prefilter: 18677, postfilter: 18441).

```
# save filtered object
saveRDS(
  object = target_Data,
  file = paste0(output_dir, '/', input_experiment, '/output/qc_results/', current_date, '/target_Data.RDS')
)
```

LS0tCnRpdGxlOiAnTmFub3N0cmluZyBHZW9NeCBhbmFseXNpczogV1RBIFF1YWxpdHkgY29udHJvbCcKYXV0aG9yOiAnUmVteSAoU2FzY2hhKScKb3V0cHV0OgogIGh0bWxfZG9jdW1lbnQ6CiAgICBjb2RlX2Rvd25sb2FkOiB0cnVlCiAgICBjb2RlX2ZvbGRpbmc6IGhpZGUKICAgIHRvYzogdHJ1ZQogICAgdG9jX2Zsb2F0OgogICAgICBjb2xsYXBzZWQ6IGZhbHNlCiAgICAgIHNtb290aF9zY3JvbGw6IHRydWUKICAgIHRvY19kZXB0aDogMwplZGl0b3Jfb3B0aW9uczoKICBtYXJrZG93bjoKICAgIHdyYXA6IDcyCi0tLQoKYGBge3Igc2V0dXAsIGluY2x1ZGU9RkFMU0V9CmtuaXRyOjpvcHRzX2NodW5rJHNldChlY2hvID0gVFJVRSwgd2FybmluZyA9IEZBTFNFKQpvcHRpb25zKAogIHNjaXBlbiA9IDk5OSwgIyByZW5kZXIgbnVtYmVycyBhcyByZWFsIG51bWJlcnMgYW5kIG5vdCBpbiBzY2llbnRpZmljIHdyaXRpbmcgc3R5bGUKICBiaXRtYXBUeXBlID0gJ2NhaXJvJyAjIGZpeCB0aGUgc2VtaS10cmFuc3BhcmVuY3kgY29sb3IgaXNzdWUgaW4gZ2dwbG90OiBzZWUgaHR0cHM6Ly9zdGFja292ZXJmbG93LmNvbS9xdWVzdGlvbnMvMjI1ODE1Njcvc2VtaS10cmFuc3BhcmVuY3ktaW4tcnN0dWRpbwogICkKYGBgCgojIERhdGFzZXQ6IEh1bWFuIFdUQQoKKipkYXRlOiBgciBmb3JtYXQoU3lzLnRpbWUoKSwgJyVIOiVNICVhICVkICVCLCAlWScpYCoqCgotLS0tLS0tLS0tLS0tLS0tLS0tLS0tLS0tLS0tLS0tLS0tLS0tLS0tLS0tLS0tLS0tLS0tLS0tLS0tLS0tLS0tLS0tLS0tLS0KCklucHV0IHZhcmlhYmxlcwoKYGBge3IgaW5wdXRfdmFyaWFibGVzfQojIGlucHV0IGV4cGVyaW1lbnQgZm9sZGVyCmlucHV0X2V4cGVyaW1lbnQgPC0gJ2V4cDInCgpxY19hc3Nlc3NtZW50X2RhdGUgPC0gJzIwMjNfMDNfMDgnICMjIyBDSEFOR0UKCiMgc2F2ZV9wbG90cyA8LSBGQUxTRSAjIG5vdCB1c2VkCnNhdmVfdGFibGVzIDwtIFRSVUUKc2F2ZV9vYmplY3QgPC0gVFJVRSAjIHNhdmUgcHJvYmUgUUMgTmFub3N0cmluZyBvYmplY3QgZm9yIGludGVybWVkaWF0ZSBwYW5lbCBjb21wYXJpc29uCmBgYAoKIyAxIExvYWRpbmcgYmFzZSBmaWxlcwoKYGBge3IgbG9hZGluZ19iYXNlX2RhdGF9CiMgUmVmZXJlbmNlIHRoZSBtYWluIGZvbGRlciAnZmlsZS5wYXRoJyBjb250YWluaW5nIHRoZSBzdWItZm9sZGVycyB3aXRoIGVhY2ggZGF0YSBmaWxlIHR5cGU6CmJhc2VfZGlyIDwtIGxpc3QoCiAgaHBjPScvaHBjL2RsYWJfc25pcHBlcnQvUDAwMDFfVDFfRUFSTFlfQ0FOQ0VSJywKICBtbnQ9Jy9ob21lL3Nhc2NoYS9ocGMvZGxhYl9zbmlwcGVydC9QMDAwMV9UMV9FQVJMWV9DQU5DRVInLAogIHVtYz0nL2hvbWUvY29nL3NicnVubmVyL2hwYy9kbGFiX3NuaXBwZXJ0L1AwMDAxX1QxX0VBUkxZX0NBTkNFUicKKQoKZm9yKGkgaW4gYmFzZV9kaXIpewogIGlmKGRpci5leGlzdHMoaSkpewogICAgYmFzZV9kaXIgPC0gaQogICAgYnJlYWsKICB9Cn0KCm91dHB1dF9kaXIgPC0gbGlzdCgKICBsb2NhbD0nL2hvbWUvc2FzY2hhL2hwYy9kbGFiX3NuaXBwZXJ0L1AwMDAxX1QxX0VBUkxZX0NBTkNFUi9wcm9jZXNzZWQvbmFub3N0cmluZycsCiAgdW1jPScvaG9tZS9jb2cvc2JydW5uZXIvaHBjL2RsYWJfc25pcHBlcnQvUDAwMDFfVDFfRUFSTFlfQ0FOQ0VSL3Byb2Nlc3NlZC9uYW5vc3RyaW5nJwopCgpmb3IoaSBpbiBvdXRwdXRfZGlyKXsKICBpZihkaXIuZXhpc3RzKGkpKXsKICAgIG91dHB1dF9kaXIgPC0gaQogICAgYnJlYWsKICB9Cn0KCnBsb3RzX291dHB1dF9kaXIgPC0gbGlzdCgKICBsb2NhbD0nL2hvbWUvc2FzY2hhJywKICB1bWM9Jy9ob21lL2NvZy9zYnJ1bm5lci9Eb2N1bWVudHMvMjAyMl9zcGF0aWFsX3RyYW5zY3JpcHRvbWljcy9yZXN1bHRzJwopCgpmb3IoaSBpbiBwbG90c19vdXRwdXRfZGlyKXsKICBpZihkaXIuZXhpc3RzKGkpKXsKICAgIHBsb3RzX291dHB1dF9kaXIgPC0gaQogICAgYnJlYWsKICB9Cn0KCiMgZ2V0IHRvZGF5cyBkYXRlCmN1cnJlbnRfZGF0ZSA8LSBmb3JtYXQoU3lzLkRhdGUoKSwgJyVZXyVtXyVkJykKYGBgCgoqKmxvYWRpbmcgZGVwZW5kZW5jaWVzKiogUGxlYXNlIG1ha2Ugc3VyZSB0aGUgZm9sbG93aW5nIHBhY2thZ2VzIGFyZQppbnN0YWxsZWQgYW5kIHJlcXVpcmVkIGxpYnJhcmllcyBjYW4gYmUgbG9hZGVkOgoKLSAgIGluc3RhbGwucGFja2FnZXMoJ2RldnRvb2xzJykKLSAgIGRldnRvb2xzOjppbnN0YWxsX2dpdGh1YignTmFub3N0cmluZy1CaW9zdGF0cy9OYW5vU3RyaW5nTkNUb29scycpCi0gICBkZXZ0b29sczo6aW5zdGFsbF9naXRodWIoJ05hbm9zdHJpbmctQmlvc3RhdHMvR2VvbXhUb29scycsIHJlZiA9CiAgICAnZGV2JykKLSAgIGRldnRvb2xzOjppbnN0YWxsX2dpdGh1YignTmFub3N0cmluZy1CaW9zdGF0cy9HZW9NeFdvcmtmbG93cycsIHJlZiA9CiAgICAnbWFpbicpCi0gICBkZXZ0b29sczo6aW5zdGFsbF9naXRodWIoJ0RhdmlzTGFib3JhdG9yeS9zdGFuZFInKQotICAgQmlvY01hbmFnZXI6Omluc3RhbGwoJ1NwYXRpYWxEZWNvbicpCi0gICBCaW9jTWFuYWdlcjo6aW5zdGFsbCgnR1NWQScpCi0gICBpbnN0YWxsLnBhY2thZ2VzKCdwbG90bHknKQotICAgaW5zdGFsbC5wYWNrYWdlcygnRFQnKQotICAgaW5zdGFsbC5wYWNrYWdlcygnbXNpZ2RicicpCgpgYGB7ciBsb2FkX2xpYnJhcmllcywgbWVzc2FnZT1GQUxTRSwgd2FybmluZz1GQUxTRX0KIyBsb2FkIGxpYnJhcmllcwpzb3VyY2UocGFzdGUwKGJhc2VfZGlyLCAnL2FuYWx5c2lzL25hbm9zdHJpbmcvcl9vYmplY3RzL2xpYnMuUicpKQoKIyBsb2FkIGNvbG9yIGNvZGVzCnNvdXJjZShwYXN0ZTAoYmFzZV9kaXIsICcvYW5hbHlzaXMvbmFub3N0cmluZy9yX29iamVjdHMvY29sb3JfY29kZXMuUicpKQphbm5fY29sb3JzIDwtIGFubl9jb2xvcnNfV1RBCmBgYAoKVG8gbG9jYXRlIGEgc3BlY2lmaWMgZmlsZSBwYXRoIHJlcGxhY2UgdGhlIGFib3ZlIGxpbmUgd2l0aCBiYXNlX2RpciBcPC0KZmlsZS5wYXRoKCdcfi9Gb2xkZXIvU3ViRm9sZGVyL0RhdGFMb2NhdGlvbicpIHJlcGxhY2UgdGhlIEZvbGRlciwKU3ViRm9sZGVyLCBEYXRhTG9jYXRpb24gYXMgbmVlZGVkLiBUaGUgRGF0YUxvY2F0aW9uIGZvbGRlciBzaG91bGQKY29udGFpbiBhIGRjY3MsIHBrY3MsIGFuZCBhbm5vdGF0aW9uIGZvbGRlciB3aXRoIGVhY2ggc2V0IG9mIGZpbGVzCnByZXNlbnQgYXMgbmVlZGVkIGF1dG9tYXRpY2FsbHkgbGlzdCBmaWxlcyBpbiBlYWNoIGRpcmVjdG9yeSBmb3IgdXNlLgoKKipUYWtlIGNhcmUgeW91IGltcG9ydCBhIGNvbHVtbiB3aXRoIG51Y2xlaSBjb3VudCBzZXBhcmF0ZWx5IGlmIHlvdQp3YW50LioqCgpgYGB7ciBwYXJzZV9maWxlc30KRENDRmlsZXMgPC0gZGlyKGZpbGUucGF0aChwYXN0ZTAoYmFzZV9kaXIsICcvcHJvY2Vzc2VkL25hbm9zdHJpbmcvJywgaW5wdXRfZXhwZXJpbWVudCksICdkY2NzJyksCiAgICAgICAgICAgICAgICBwYXR0ZXJuID0gJy5kY2MkJywKICAgICAgICAgICAgICAgIGZ1bGwubmFtZXMgPSBUUlVFLAogICAgICAgICAgICAgICAgcmVjdXJzaXZlID0gVFJVRSkKUEtDRmlsZXMgPC0gZGlyKGZpbGUucGF0aChwYXN0ZTAoYmFzZV9kaXIsICcvcHJvY2Vzc2VkL25hbm9zdHJpbmcvJywgaW5wdXRfZXhwZXJpbWVudCksICdwa2NzJyksCiAgICAgICAgICAgICAgICBwYXR0ZXJuID0gJy5wa2MkJywKICAgICAgICAgICAgICAgIGZ1bGwubmFtZXMgPSBUUlVFLAogICAgICAgICAgICAgICAgcmVjdXJzaXZlID0gVFJVRSkKU2FtcGxlQW5ub3RhdGlvbkZpbGUgPC0gZGlyKGZpbGUucGF0aChwYXN0ZTAoYmFzZV9kaXIsICcvcHJvY2Vzc2VkL25hbm9zdHJpbmcvJywgaW5wdXRfZXhwZXJpbWVudCksICdhbm5vdGF0aW9uJyksCiAgICAgICAgICAgICAgICAgICAgICAgICAgICBwYXR0ZXJuID0gJ2ZpbmFsLnhsc3gkJywKICAgICAgICAgICAgICAgICAgICAgICAgICAgIGZ1bGwubmFtZXMgPSBUUlVFLAogICAgICAgICAgICAgICAgICAgICAgICAgICAgcmVjdXJzaXZlID0gVFJVRSkKYGBgCgojIDIgTG9hZCBkYXRhCgpgYGB7ciBsb2FkX2RhdGF9CkRhdGEgPC0gcmVhZE5hbm9TdHJpbmdHZW9NeFNldChkY2NGaWxlcyA9IERDQ0ZpbGVzLAogICAgICAgICAgICAgICAgICAgICAgICAgICAgICAgcGtjRmlsZXMgPSBQS0NGaWxlcywKICAgICAgICAgICAgICAgICAgICAgICAgICAgICAgICMgY2hlY2sgdGhhdCBjb2xuYW1lcyBhcmUgY29ycmVjdCwgb3JkZXIgYnkgU2FtcGxlX0lEIGZvciBwcm9wZXIgTlRDIGNhbGN1bGF0aW9uIQogICAgICAgICAgICAgICAgICAgICAgICAgICAgICAgcGhlbm9EYXRhRmlsZSA9IFNhbXBsZUFubm90YXRpb25GaWxlLAogICAgICAgICAgICAgICAgICAgICAgICAgICAgICAgcGhlbm9EYXRhU2hlZXQgPSAnTWV0YWRhdGEnLCAjICJNZXRhZGF0YSIKICAgICAgICAgICAgICAgICAgICAgICAgICAgICAgIHBoZW5vRGF0YURjY0NvbE5hbWUgPSAnU2FtcGxlX0lEJywKICAgICAgICAgICAgICAgICAgICAgICAgICAgICAgIHByb3RvY29sRGF0YUNvbE5hbWVzID0gYygnYW9pJywgJ3JvaScpLAogICAgICAgICAgICAgICAgICAgICAgICAgICAgICAgZXhwZXJpbWVudERhdGFDb2xOYW1lcyA9IGMoJ3BhbmVsJykpCgpwYXN0ZSgnUmVhZHMgZnJvbSBmb2xsb3dpbmcgcnVucyB1c2VkOiAnLCB1bmlxdWUocERhdGEocHJvdG9jb2xEYXRhKERhdGEpKSRTZXFTZXRJZCkpCmBgYAoKIyAzIFN0dWR5IGRlc2lnbgoKYGBge3IgYW5ub3RhdGV9CnBrY3MgPC0gYW5ub3RhdGlvbihEYXRhKQptb2R1bGVzIDwtIGdzdWIoJy5wa2MnLCAnJywgcGtjcykKa2FibGUoZGF0YS5mcmFtZShQS0NzID0gcGtjcywgbW9kdWxlcyA9IG1vZHVsZXMpKQpgYGAKClNlbGVjdCB0aGUgYW5ub3RhdGlvbnMgd2Ugd2FudCB0byBzaG93LCB1c2UgXGBcYCB0byBzdXJyb3VuZCBjb2x1bW4KbmFtZXMgd2l0aCBzcGFjZXMgb3Igc3BlY2lhbCBzeW1ib2xzLgoKYGBge3Igc2VsZWN0X2Fubm90YXRpb25zfQphbm5fc2VsZWN0aW9uIDwtIGxpc3QoCiAgYW5ub3RhdGlvbnMgPSBjKCdzbGlkZSBuYW1lJywgJ1BhdGllbnRfSUQnLCAnc2xpZGVMb2NhdGlvbicsICdzZWdtZW50JywgJ3Rpc3N1ZVJlZ2lvbicpLAogIGFubm90YXRpb25fbGFiZWxzID0gYygnU2xpZGUgbmFtZScsICdQYXRpZW50IElEJywgJ1NsaWRlIExvY2F0aW9uJywgJ1NlZ21lbnQnLCAnVGlzc3VlIFJlZ2lvbicpCikKCiMgY291bnQgaG93IG1hbnkgUk9JcyBwZXIgYW5ub3RhdGlvbgpjb3VudF9tYXQgPC0gZHBseXI6OmNvdW50KHBEYXRhKERhdGEpLCBhY3Jvc3MoYWxsX29mKGFubl9zZWxlY3Rpb24kYW5ub3RhdGlvbnMpKSkKYGBgCgpTaW1wbGlmeSB0aGUgc2xpZGUgbmFtZXMgaWYgcmVxdWlyZWQuCgpgYGB7ciBzaW1wbGlmeV9uYW1lc30KIyAkJ3NsaWRlIG5hbWUnIDwtIGdzdWIoJ2Rpc2Vhc2UnLCAnZCcsIGdzdWIoJ25vcm1hbCcsICduJywgY291bnRfbWF0JCdzbGlkZSBuYW1lJykpCmBgYAoKR2F0aGVyIHRoZSBkYXRhIGFuZCBwbG90IGluIG9yZGVyLgoKYGBge3IgZ2F0aGVyX2RhdGF9CnRlc3RfZ3IgPC0gZ2F0aGVyX3NldF9kYXRhKGNvdW50X21hdCwgMTpsZW5ndGgoYW5uX3NlbGVjdGlvbiRhbm5vdGF0aW9ucykpCnRlc3RfZ3IkeCA8LSBmYWN0b3IodGVzdF9nciR4LCBsYWJlbHMgPSBhbm5fc2VsZWN0aW9uJGFubm90YXRpb25zKQpgYGAKCiMjIDMuMSBQbG90IFNhbmtleQoKYGBge3IgU2Fua2V5X3Bsb3QsIGZpZy53aWR0aD0yMCxmaWcuaGVpZ2h0PTExfQp0ZXN0X2dyICU+JQpnZ3Bsb3QoLiwgaGVpZ2h0ID0gMTUsIHdpZHRoID0gMTUsIHVuaXQgPSAnY20nLCBhZXMoeCwgaWQgPSBpZCwgc3BsaXQgPSB5LCB2YWx1ZSA9IG4pKSArCiAgZ2VvbV9wYXJhbGxlbF9zZXRzKGFlcyhmaWxsID0gc2VnbWVudCksIGFscGhhID0gMC41LCBheGlzLndpZHRoID0gMC40NSkgKwogIGdlb21fcGFyYWxsZWxfc2V0c19heGVzKGFlcyhmaWxsID0gaWZfZWxzZShzZWdtZW50ID09IHksIHNlZ21lbnQsICdncmV5JykpLAogICAgICAgICAgICAgICAgICAgICAgICAgIGF4aXMud2lkdGggPSAwLjQ1LCBjb2xvciA9ICdibGFjaycsIGZpbGwgPSAnZ3JleScpICsKICBnZW9tX3BhcmFsbGVsX3NldHNfbGFiZWxzKGNvbG9yID0gJ2JsYWNrJywgc2l6ZSA9IDUsIGFuZ2xlID0gMCkgKwogIHNjYWxlX3lfY29udGludW91cyhleHBhbmQgPSBleHBhbnNpb24oMCkpICsKICBzY2FsZV94X2Rpc2NyZXRlKGV4cGFuZCA9IGV4cGFuc2lvbigwKSwgbGFiZWxzID0gYW5uX3NlbGVjdGlvbiRhbm5vdGF0aW9uX2xhYmVscywgcG9zaXRpb24gPSAndG9wJykgKwogIGxhYnMoeCA9ICcnLCB5ID0gJycpICsKICBzY2FsZV9maWxsX21hbnVhbCh2YWx1ZXMgPSBhbm5fY29sb3JzJHNlZ21lbnQpICsKICBhbm5vdGF0ZShnZW9tID0gJ3NlZ21lbnQnLCB4ID0gNC40LCB4ZW5kID0gNC40LAogICAgICAgICAgIHkgPSAxMCwgeWVuZCA9IDExMCwgbHdkID0gMikgKwogIGFubm90YXRlKGdlb20gPSAndGV4dCcsIHggPSA0LjM0LCB5ID0gNTAsIGFuZ2xlID0gOTAsIHNpemUgPSA1LAogICAgICAgICAgIGhqdXN0ID0gMC41LCBsYWJlbCA9ICcxMDAgc2VnbWVudHMnKSArCiAgdGhlbWVfY2xhc3NpYyhiYXNlX3NpemUgPSAxMikgKwogIHRoZW1lKGxlZ2VuZC5wb3NpdGlvbiA9ICdub25lJywKICAgICAgICBsZWdlbmQudGl0bGUgPSBlbGVtZW50X2JsYW5rKCksCiAgICAgICAgbGVnZW5kLnRleHQgPSBlbGVtZW50X3RleHQoc2l6ZSA9IDE1KSwKICAgICAgICBwYW5lbC5iYWNrZ3JvdW5kID0gZWxlbWVudF9yZWN0KGZpbGwgPSAnd2hpdGUnKSwKICAgICAgICBheGlzLnRpY2tzLnggPSBlbGVtZW50X2JsYW5rKCksCiAgICAgICAgYXhpcy50aWNrcy55ID0gZWxlbWVudF9ibGFuaygpLAogICAgICAgIGF4aXMubGluZSA9IGVsZW1lbnRfYmxhbmsoKSwKICAgICAgICAjIGF4aXMudGV4dC54ID0gZWxlbWVudF9ibGFuaygpLAogICAgICAgIGF4aXMudGV4dC54ID0gZWxlbWVudF90ZXh0KHNpemUgPSAxNiwgZmFjZSA9ICdib2xkJyksCiAgICAgICAgYXhpcy50ZXh0LnkgPSBlbGVtZW50X2JsYW5rKCksCiAgICAgICAgcGxvdC5tYXJnaW4gPSBtYXJnaW4oMSwxLDEsMSwgdW5pdCA9ICdjbScpKQpgYGAKCiMgNCBRQyAmIFByZS1wcm9jZXNzaW5nCgpTaGlmdCBjb3VudHMgdG8gb25lCgpgYGB7ciBzaGlmdF9jb3VudHN9CiMgc2hpZnQgYW55IGV4cHJlc3Npb24gY291bnRzIHdpdGggYSB2YWx1ZSBvZiAwIHRvIDEgdG8gZW5hYmxlIGluIGRvd25zdHJlYW0gdHJhbnNmb3JtYXRpb25zLgpEYXRhIDwtIHNoaWZ0Q291bnRzT25lKERhdGEsIHVzZURBTG9naWMgPSBUUlVFKQpgYGAKCiMjIDQuMSBTZWdtZW50IFFDCgpXZSBmaXJzdCBhc3Nlc3Mgc2VxdWVuY2luZyBxdWFsaXR5IGFuZCBhZGVxdWF0ZSB0aXNzdWUgc2FtcGxpbmcgZm9yCmV2ZXJ5IFJPSS9BT0kgc2VnbWVudC4gRXZlcnkgUk9JL0FPSSBzZWdtZW50IHdpbGwgYmUgdGVzdGVkIGZvcjo8YnI+Ci0gKipSYXcgc2VxdWVuY2luZyByZWFkcyoqOiBzZWdtZW50cyB3aXRoIFw8MTAwMCByYXcgcmVhZHMgYXJlIHJlbW92ZWQuPGJyPgotICoqJSBBbGlnbmVkLCUgVHJpbW1lZCwgb3IgJSBTdGl0Y2hlZCBzZXF1ZW5jaW5nIHJlYWRzKio6IFNlZ21lbnRzIHdpdGggcmVhZHMKYmVsb3cgXH44MCUgb2YgdGhlIHJhdyByZWFkcyBudW1iZXIgZm9yIG9uZSBvciBtb3JlIG9mIHRoZXNlIFFDCnBhcmFtZXRlcnMgYXJlIHJlbW92ZWQuPGJyPgotICoqJSBTZXF1ZW5jaW5nIHNhdHVyYXRpb24gKFsxIC0gZGVkdXBsaWNhdGVkIHJlYWRzL2FsaWduZWQgcmVhZHNdJSkqKjogCjEwMCUgc2VxdWVuY2luZyBzYXR1cmF0aW9uIGluZGljYXRlcyBhIHJlcHJlc2VudGF0aXZlIHNhbXBsZSwgd2hpbGUgMCUgc2VxdWVuY2luZyBzYXR1cmF0aW9uIGluZGljYXRlcyB0aGF0IGFsbApyZWFkcyB3ZXJlIHVuaXF1ZS4gU2VnbWVudHMgYmVsb3cgXH41MCUgcmVxdWlyZSBhZGRpdGlvbmFsIHNlcXVlbmNpbmcgdG8KY2FwdHVyZSBmdWxsIHNhbXBsZSBkaXZlcnNpdHkgYW5kIGFyZSBub3QgdHlwaWNhbGx5IGFuYWx5emVkIHVudGlsIGltcHJvdmVkLjxicj4KLSAqKk5lZ2F0aXZlIENvdW50Kio6IFRoaXMgaXMgdGhlIGdlb21ldHJpYyBtZWFuIG9mIHRoZSBzZXZlcmFsCnVuaXF1ZSBuZWdhdGl2ZSBwcm9iZXMgaW4gdGhlIEdlb014IHBhbmVsIHRoYXQgZG8gbm90IHRhcmdldCBhbnkgbVJOQQphbmQgZXN0YWJsaXNoIHRoZSBiYWNrZ3JvdW5kIGxldmVsIG9mIHRlY2huaWNhbCBub2lzZSBleHBlY3RlZC4gSXQgaXMKY2FsY3VsYXRlZCBwZXIgc2VnbWVudDsgc2VnbWVudHMgd2l0aCBsb3cgbmVnYXRpdmUgY291bnRzICgxLTEwKSBhcmUgbm90Cm5lY2Vzc2FyaWx5IHJlbW92ZWQgYnV0IG1heSBiZSBzdHVkaWVkIGNsb3NlciBmb3IgbG93IGVuZG9nZW5vdXMgZ2VuZQpzaWduYWwgYW5kL29yIGluc3VmZmljaWVudCB0aXNzdWUgc2FtcGxpbmcuPGJyPgotICoqTm8gVGVtcGxhdGUgQ29udHJvbCAoTlRDKSBjb3VudCoqOiBWYWx1ZXMgXD4xLDAwMCBjb3VsZCBpbmRpY2F0ZSBjb250YW1pbmF0aW9uIGZvciB0aGUgc2VnbWVudHMKYXNzb2NpYXRlZCB3aXRoIHRoaXMgTlRDOyBob3dldmVyLCBpbiBjYXNlcyB3aGVyZSB0aGUgTlRDIGNvdW50IGlzCmJldHdlZW4gMSwwMDAtMTAsMDAwLCB0aGUgc2VnbWVudHMgbWF5IGJlIHVzZWQgaWYgdGhlIE5UQyBkYXRhIGlzCnVuaWZvcm1seSBsb3cgKGUuZy4gMC0yIGNvdW50cyBmb3IgYWxsIHByb2JlcykuPGJyPgotICoqTnVjbGVpKio6IFw+MTAwIG51Y2xlaSBwZXIgc2VnbWVudCBpcyBnZW5lcmFsbHkgcmVjb21tZW5kZWQ7IApob3dldmVyLCB0aGlzIGN1dG9mZiBpcyBoaWdobHkgc3R1ZHkvdGlzc3VlIGRlcGVuZGVudCBhbmQgbWF5IG5lZWQgdG8gYmUgcmVkdWNlZC4gCldoYXQgaXMgbW9zdCBpbXBvcnRhbnQgaXMgY29uc2lzdGVuY3kgaW4gdGhlIG51Y2xlaSBkaXN0cmlidXRpb24gZm9yIHNlZ21lbnRzIHdpdGhpbgp0aGUgc3R1ZHkuPGJyPgotICoqQXJlYSoqOiBHZW5lcmFsbHkgY29ycmVsYXRlcyB3aXRoIG51Y2xlaTsgYSBzdHJpY3QgY3V0b2ZmIGlzCm5vdCBnZW5lcmFsbHkgYXBwbGllZCBiYXNlZCBvbiBhcmVhLiAKCiMjIyA0LjEuMSBTZWxlY3QgU2VnbWVudCBRQwoKRmlyc3QsIHdlIHNlbGVjdCB0aGUgUUMgcGFyYW1ldGVyIGN1dG9mZnMsIGFnYWluc3Qgd2hpY2ggb3VyIFJPSS9BT0kKc2VnbWVudHMgd2lsbCBiZSB0ZXN0ZWQgYW5kIGZsYWdnZWQgYXBwcm9wcmlhdGVseS4gV2UgaGF2ZSBzZWxlY3RlZCB0aGUKYXBwcm9wcmlhdGUgc3R1ZHktc3BlY2lmaWMgcGFyYW1ldGVycyBmb3IgdGhpcyBzdHVkeS4gTm90ZTogdGhlIGRlZmF1bHQKUUMgdmFsdWVzIHJlY29tbWVuZGVkIGFib3ZlIGFyZSBhZHZpc2VkIHdoZW4gc3VydmV5aW5nIGEgbmV3IGRhdGFzZXQgZm9yCnRoZSBmaXJzdCB0aW1lLiBEZWZhdWx0IFFDIGN1dG9mZnMgYXJlIGNvbW1lbnRlZCBpbiAoKSBhZGphY2VudCB0byB0aGUKcmVzcGVjdGl2ZSBwYXJhbWV0ZXJzIHN0dWR5LXNwZWNpZmljIHZhbHVlcyB3ZXJlIHNlbGVjdGVkIGFmdGVyIHZpc3VhbGl6aW5nIHRoZSBRQy48YnI+ClJlc3VsdHMgaW4gbW9yZSBkZXRhaWwgYmVsb3cuCgpgYGB7ciBzZXRfc2VnbWVudFFDX3BhcmFtc30Kc2VnbWVudFFDX3BhcmFtcyA8LQogIGxpc3QobWluc2VnbWVudFJlYWRzID0gMTAwMCwgIyBNaW5pbXVtIG51bWJlciBvZiByZWFkcyAoMTAwMCkKICAgICAgIHBlcmNlbnRUcmltbWVkID0gODAsICAgICMgTWluaW11bSAlIG9mIHJlYWRzIHRyaW1tZWQgKDgwJSkKICAgICAgIHBlcmNlbnRTdGl0Y2hlZCA9IDgwLCAgICMgTWluaW11bSAlIG9mIHJlYWRzIHN0aXRjaGVkICg4MCUpCiAgICAgICBwZXJjZW50QWxpZ25lZCA9IDgwLCAgICAjIE1pbmltdW0gJSBvZiByZWFkcyBhbGlnbmVkICg4MCUpCiAgICAgICBwZXJjZW50U2F0dXJhdGlvbiA9IDUwLCAjIE1pbmltdW0gc2VxdWVuY2luZyBzYXR1cmF0aW9uICg1MCUpCiAgICAgICBtaW5OZWdhdGl2ZUNvdW50ID0gMSwgICAjIE1pbmltdW0gbmVnYXRpdmUgY29udHJvbCBjb3VudHMgKDEwKSwgbWF5YmUgY2hvb3NlIDIgYmFzZWQgb24gdGhlIG5lZ0dlb01lYW5zCiAgICAgICBtYXhOVENDb3VudCA9IDEyMDAwLCAgICAgIyBNYXhpbXVtIGNvdW50cyBvYnNlcnZlZCBpbiBOVEMgd2VsbCAoMTAwMCkKICAgICAgIG1pbk51Y2xlaSA9IDEwMCwgICAgICAgICAjIE1pbmltdW0gIyBvZiBudWNsZWkgZXN0aW1hdGVkICgxMDApCiAgICAgICBtaW5BcmVhID0gNTAwMCkgICAgICAgICAjIE1pbmltdW0gc2VnbWVudCBhcmVhICg1MDAwKQoKIyAjIHNldCBjb3JyZWN0IGNvbHVtbiBuYW1lcyBmb3IgUUMgZmxhZ3MgZnVuY3Rpb25zCiMgIyBzZXROdWNsZWlGbGFncygpIGV4cGVjdHMgYSBjb2x1bW4gJ251Y2xlaScgKGxvd2VyIGNhc2UpCiMgcERhdGEoRGF0YSkkbnVjbGVpIDwtIHBEYXRhKERhdGEpWywgJ0FPSU51Y2xlaUNvdW50J10KIyBwRGF0YShEYXRhKSA8LSBzdWJzZXQocERhdGEoRGF0YSksIHNlbGVjdCA9IC1jKEFPSU51Y2xlaUNvdW50KSkKIyAjIHNldEFyZWFGbGFncygpIGV4cGVjdHMgYSBjb2x1bW4gJ2FyZWEnIChsb3dlciBjYXNlKQojIHBEYXRhKERhdGEpJGFyZWEgPC0gcERhdGEoRGF0YSlbLCAnQXJlYSddCgpEYXRhIDwtIHNldFNlZ21lbnRRQ0ZsYWdzKERhdGEsIHFjQ3V0b2ZmcyA9IHNlZ21lbnRRQ19wYXJhbXMpCgpzZWdtZW50UUNwYXJhbXNfZGYgPC0gZGF0YS5mcmFtZSAoCiAgaXRlbXMgPSBjKCdtaW5zZWdtZW50UmVhZHMnLCAncGVyY2VudFRyaW1tZWQnLCAncGVyY2VudFN0aXRjaGVkJywKICAgICAgICAgICAgJ3BlcmNlbnRBbGlnbmVkJywgJ3BlcmNlbnRTYXR1cmF0aW9uJywgJ21pbk5lZ2F0aXZlQ291bnQnLAogICAgICAgICAgICAnbWF4TlRDQ291bnQnLCAnbWluTnVjbGVpJywgJ21pbkFyZWEnKSwKICBkZWZhdWx0cyA9IGMoMTAwMCwgODAsIDgwLAogICAgICAgICAgICAgICA4MCwgNTAsIDEwLAogICAgICAgICAgICAgICAxMDAwLCAxMDAsIDUwMDApLAogIGFjdHVhbCA9IGMoc2VnbWVudFFDX3BhcmFtcyRtaW5zZWdtZW50UmVhZHMsIHNlZ21lbnRRQ19wYXJhbXMkcGVyY2VudFRyaW1tZWQsIHNlZ21lbnRRQ19wYXJhbXMkcGVyY2VudFN0aXRjaGVkLAogICAgICAgICAgICAgc2VnbWVudFFDX3BhcmFtcyRwZXJjZW50QWxpZ25lZCwgc2VnbWVudFFDX3BhcmFtcyRwZXJjZW50U2F0dXJhdGlvbiwgc2VnbWVudFFDX3BhcmFtcyRtaW5OZWdhdGl2ZUNvdW50LAogICAgICAgICAgICAgc2VnbWVudFFDX3BhcmFtcyRtYXhOVENDb3VudCwgc2VnbWVudFFDX3BhcmFtcyRtaW5OdWNsZWksIHNlZ21lbnRRQ19wYXJhbXMkbWluQXJlYSkKKQoKZGF0YXRhYmxlKHNlZ21lbnRRQ3BhcmFtc19kZiwgcm93bmFtZXMgPSBGQUxTRSwKICAgICAgICAgIGNhcHRpb24gPSAnUUMgdGhyZXNob2xkcycsCiAgICAgICAgICBleHRlbnNpb25zID0gJ0J1dHRvbnMnLAogICAgICAgICAgb3B0aW9ucyA9IGxpc3QoCiAgICAgICAgICAgIGRvbSA9ICdCZnRyaXAnLAogICAgICAgICAgICBidXR0b25zID0gYygnY29weScsICdjc3YnLCAnZXhjZWwnLCAncGRmJywgJ3ByaW50JykKICAgICAgICAgICkKKQpgYGAKCiMjIyBDb2xsYXRlIFFDIFJlc3VsdHMgey50YWJzZXQgLnRhYnNldC1waWxsc30KCmBgYHtyIGNvbGxhdGVfUUNfcmVzdWx0c30Kc2VnbWVudFFDUmVzdWx0cyA8LSBwcm90b2NvbERhdGEoRGF0YSlbWydRQ0ZsYWdzJ11dCmZsYWdfY29sdW1ucyA8LSBjb2xuYW1lcyhzZWdtZW50UUNSZXN1bHRzKQpzZWdtZW50UUNfU3VtbWFyeSA8LSBkYXRhLmZyYW1lKFBhc3MgPSBjb2xTdW1zKCFzZWdtZW50UUNSZXN1bHRzWywgZmxhZ19jb2x1bW5zXSksCiAgICAgICAgICAgICAgICAgICAgICAgICBXYXJuaW5nID0gY29sU3VtcyhzZWdtZW50UUNSZXN1bHRzWywgZmxhZ19jb2x1bW5zXSkpCnNlZ21lbnRRQ1Jlc3VsdHMkUUNTdGF0dXMgPC0gYXBwbHkoc2VnbWVudFFDUmVzdWx0cywgMUwsIGZ1bmN0aW9uKHgpIHsKICBpZmVsc2Uoc3VtKHgpID09IDBMLCAnUEFTUycsICdXQVJOSU5HJykKfSkKCnNlZ21lbnRRQ1Jlc3VsdHMgPC0gc2VnbWVudFFDUmVzdWx0cyAlPiUKICBtdXRhdGUobkZsYWdzID0gcm93U3VtcyhhY3Jvc3Mod2hlcmUoaXMubG9naWNhbCkpKSkKCnRtcF9zZGF0YSA8LSBzRGF0YShEYXRhKSAlPiUKICBzZWxlY3Qoc2VnbWVudCwgcm9pLCBQYXRpZW50X0lELCB0aXNzdWVSZWdpb24sIG51Y2xlaSwgYHNsaWRlIG5hbWVgKSAlPiUKICBtdXRhdGUoUmVwbGljYXRlX0lEID0gcGFzdGUwKFBhdGllbnRfSUQsICdfJywgdGlzc3VlUmVnaW9uLCAnXycsIHNlZ21lbnQpKSAlPiUKICBtdXRhdGUoUmVwbGljYXRlX0lEID0gZmFjdG9yKFJlcGxpY2F0ZV9JRCkpICU+JQogIG11dGF0ZShSZXBsaWNhdGVfSURfbnVtID0gYXMubnVtZXJpYyhSZXBsaWNhdGVfSUQpKQoKc2VnbWVudFFDUmVzdWx0c19wcmUgPC0gbWVyZ2UodG1wX3NkYXRhLCBzZWdtZW50UUNSZXN1bHRzLCBieSA9IDApCmBgYAoKIyMjIyBTZWdtZW50CgpgYGB7cn0Kc2VnbWVudFFDUmVzdWx0c19zZWdtZW50IDwtIHNlZ21lbnRRQ1Jlc3VsdHNfcHJlICU+JQogIGdyb3VwX2J5KHNlZ21lbnQpICU+JQogIHN1bW1hcmlzZShhY3Jvc3MoTG93UmVhZHM6TG93QXJlYSwgc3VtKSkgJT4lCiAgY29sdW1uX3RvX3Jvd25hbWVzKHZhciA9ICdzZWdtZW50JykgJT4lCiAgdCgpICU+JQogIGJpbmRfY29scyhzZWdtZW50UUNfU3VtbWFyeSwgLikKCmRhdGF0YWJsZShzZWdtZW50UUNSZXN1bHRzX3NlZ21lbnQsCiAgICAgICAgICBjYXB0aW9uID0gJ1JPSXMgZmxhZ2dlZCBwZXIgc2VnbWVudCcsCiAgICAgICAgICBleHRlbnNpb25zID0gJ0J1dHRvbnMnLAogICAgICAgICAgb3B0aW9ucyA9IGxpc3QgKAogICAgICAgICAgICBkb20gPSAnQmZ0cmlwJywKICAgICAgICAgICAgYnV0dG9ucyA9IGMoJ2NvcHknLCAnY3N2JywgJ2V4Y2VsJywgJ3BkZicsICdwcmludCcpCiAgICAgICAgICApCikKYGBgCgojIyMjIFBhdGllbnQKCmBgYHtyfQpzZWdtZW50UUNSZXN1bHRzX3BhdGllbnQgPC0gc2VnbWVudFFDUmVzdWx0c19wcmUgJT4lCiAgZ3JvdXBfYnkoUGF0aWVudF9JRCkgJT4lCiAgc3VtbWFyaXNlKGFjcm9zcyhMb3dSZWFkczpMb3dBcmVhLCBzdW0pKSAlPiUKICBjb2x1bW5fdG9fcm93bmFtZXModmFyID0gJ1BhdGllbnRfSUQnKSAlPiUKICB0KCkgJT4lCiAgYmluZF9jb2xzKHNlZ21lbnRRQ19TdW1tYXJ5LCAuKQoKZGF0YXRhYmxlKHNlZ21lbnRRQ1Jlc3VsdHNfcGF0aWVudCwKICAgICAgICAgIGNhcHRpb24gPSAnUk9JcyBmbGFnZ2VkIHBlciBwYXRpZW50JywKICAgICAgICAgIGV4dGVuc2lvbnMgPSAnQnV0dG9ucycsCiAgICAgICAgICBvcHRpb25zID0gbGlzdCAoCiAgICAgICAgICAgIGRvbSA9ICdCZnRyaXAnLAogICAgICAgICAgICBidXR0b25zID0gYygnY29weScsICdjc3YnLCAnZXhjZWwnLCAncGRmJywgJ3ByaW50JykKICAgICAgICAgICkKKQpgYGAKCiMjIyMgUmVnaW9uCgpgYGB7cn0Kc2VnbWVudFFDUmVzdWx0c19yZWdpb24gPC0gc2VnbWVudFFDUmVzdWx0c19wcmUgJT4lCiAgZ3JvdXBfYnkodGlzc3VlUmVnaW9uKSAlPiUKICBzdW1tYXJpc2UoYWNyb3NzKExvd1JlYWRzOkxvd0FyZWEsIHN1bSkpICU+JQogIGNvbHVtbl90b19yb3duYW1lcyh2YXIgPSAndGlzc3VlUmVnaW9uJykgJT4lCiAgdCgpICU+JQogIGJpbmRfY29scyhzZWdtZW50UUNfU3VtbWFyeSwgLikKCmRhdGF0YWJsZShzZWdtZW50UUNSZXN1bHRzX3JlZ2lvbiwKICAgICAgICAgIGNhcHRpb24gPSAnUk9JcyBmbGFnZ2VkIHBlciByZWdpb24nLAogICAgICAgICAgZXh0ZW5zaW9ucyA9ICdCdXR0b25zJywKICAgICAgICAgIG9wdGlvbnMgPSBsaXN0ICgKICAgICAgICAgICAgZG9tID0gJ0JmdHJpcCcsCiAgICAgICAgICAgIGJ1dHRvbnMgPSBjKCdjb3B5JywgJ2NzdicsICdleGNlbCcsICdwZGYnLCAncHJpbnQnKQogICAgICAgICAgKQopCmBgYAoKIyMjIyBTbGlkZSBuYW1lCgpgYGB7ciwgbWVzc2FnZT1GQUxTRSwgd2FybmluZz1GQUxTRX0Kc2VnbWVudFFDUmVzdWx0c19zbGlkZV9uYW1lIDwtIHNlZ21lbnRRQ1Jlc3VsdHNfcHJlICU+JQogIGdyb3VwX2J5KGBzbGlkZSBuYW1lYCkgJT4lCiAgc3VtbWFyaXNlKGFjcm9zcyhMb3dSZWFkczpMb3dBcmVhLCBzdW0pKSAlPiUKICBjb2x1bW5fdG9fcm93bmFtZXModmFyID0gJ3NsaWRlIG5hbWUnKSAlPiUKICB0KCkgJT4lCiAgYmluZF9jb2xzKHNlZ21lbnRRQ19TdW1tYXJ5LCAuKQoKZGF0YXRhYmxlKHNlZ21lbnRRQ1Jlc3VsdHNfc2xpZGVfbmFtZSwKICAgICAgICAgIGNhcHRpb24gPSAnUk9JcyBmbGFnZ2VkIHBlciBzbGlkZScsCiAgICAgICAgICBleHRlbnNpb25zID0gJ0J1dHRvbnMnLAogICAgICAgICAgb3B0aW9ucyA9IGxpc3QgKAogICAgICAgICAgICBkb20gPSAnQmZ0cmlwJywKICAgICAgICAgICAgYnV0dG9ucyA9IGMoJ2NvcHknLCAnY3N2JywgJ2V4Y2VsJywgJ3BkZicsICdwcmludCcpCiAgICAgICAgICApCikKYGBgCgojIyMgUmVwbGljYXRlcyBwZXIgUk9JIGxvc3QgYWZ0ZXIgU2VnbWVudCBRQwoKYGBge3IsIG1lc3NhZ2U9RkFMU0UsIHdhcm5pbmc9RkFMU0V9CiMgaG93IG1hbnkgcmVwbGljYXRlcyBwZXIgUmVwbGljYXRlX0lEIGRvIHdlIGxvc2U/CnNlZ21lbnRRQ1Jlc3VsdHNfcmVwbGljYXRlcyA8LSBzZWdtZW50UUNSZXN1bHRzX3ByZSAlPiUKICBncm91cF9ieShSZXBsaWNhdGVfSUQsIFFDU3RhdHVzKSAlPiUKICBzdW1tYXJpc2Uobl9yZXBfZmxhZyA9IG4oKSkgJT4lCiAgZ3JvdXBfYnkoUmVwbGljYXRlX0lEKSAlPiUKICBtdXRhdGUobl9yZXAgPSBzdW0obl9yZXBfZmxhZykpICU+JQogIHVuZ3JvdXAoKSAlPiUKICBtdXRhdGUoCiAgICBQcm9wRmxhZ2dlZCA9IHJvdW5kKChuX3JlcF9mbGFnIC8gbl9yZXApLCAyKSwKICAgIFByb3BGbGFnZ2VkTGFiZWwgPSBwYXN0ZTAobl9yZXBfZmxhZywgJy8nLCBuX3JlcCkpICU+JQogIG11dGF0ZSgKICAgIFJlcGxpY2F0ZVdhcm5pbmcgPSBjYXNlX3doZW4oCiAgICAgIFFDU3RhdHVzID09ICdXQVJOSU5HJyAmIG5fcmVwIC0gbl9yZXBfZmxhZyA9PSAwIH4gJ05PIFJFUExJQ0FURVMgTEVGVCcsCiAgICAgIFFDU3RhdHVzID09ICdXQVJOSU5HJyAmIG5fcmVwIC0gbl9yZXBfZmxhZyA9PSAxIH4gJzEgUkVQTElDQVRFUyBMRUZUJywKICAgICAgVFJVRSB+IE5BX2NoYXJhY3Rlcl8KICAgICkKICApICU+JQogIHNlbGVjdCgtbl9yZXBfZmxhZywgLW5fcmVwKSAlPiUKICBhcnJhbmdlKGRlc2MoUUNTdGF0dXMpLCBkZXNjKFByb3BGbGFnZ2VkKSwgUmVwbGljYXRlV2FybmluZykKCmRhdGF0YWJsZShzZWdtZW50UUNSZXN1bHRzX3JlcGxpY2F0ZXMsIHJvd25hbWVzPUZBTFNFLAogICAgICAgICAgY2FwdGlvbiA9ICdOdW1iZXIgb2YgcmVwbGljYXRlcyBwZXIgUmVwbGljYXRlX0lEIHRoYXQgd2VyZSBmbGFnZ2VkJywKICAgICAgICAgIGV4dGVuc2lvbnMgPSAnQnV0dG9ucycsCiAgICAgICAgICBvcHRpb25zID0gbGlzdCAoCiAgICAgICAgICAgIGRvbSA9ICdCZnRyaXAnLAogICAgICAgICAgICBidXR0b25zID0gYygnY29weScsICdjc3YnLCAnZXhjZWwnLCAncGRmJywgJ3ByaW50JykKICAgICAgICAgICkKKQpgYGAKCiMjIyA0LjEuMiBHcmFwaGljYWwgc3VtbWFyaWVzIG9mIFFDIHN0YXRpc3RpY3Mgey50YWJzZXQgLnRhYnNldC1waWxsc30KCmBgYHtyIFFDX3Bsb3R0aW5nfQpzZWdtZW50UUNfU3VtbWFyeVsnVE9UQUwgRkxBR1MnLCBdIDwtIGMoc3VtKHNlZ21lbnRRQ1Jlc3VsdHNbLCAnUUNTdGF0dXMnXSA9PSAnUEFTUycpLAogICAgICAgICAgICAgICAgICAgICAgICAgICAgICAgICBzdW0oc2VnbWVudFFDUmVzdWx0c1ssICdRQ1N0YXR1cyddID09ICdXQVJOSU5HJykpCgojIGNvbHVtbiB0byBmaWxsIHBsb3RzIGJ5CmNvbF9ieSA8LSAnc2VnbWVudCcKClFDX2hpc3RvZ3JhbSA8LSBmdW5jdGlvbihhc3NheV9kYXRhID0gTlVMTCwKICAgICAgICAgICAgICAgICAgICAgICAgIGFubm90YXRpb24gPSBOVUxMLAogICAgICAgICAgICAgICAgICAgICAgICAgZmlsbF9ieSA9IE5VTEwsCiAgICAgICAgICAgICAgICAgICAgICAgICB0aHIgPSBOVUxMLAogICAgICAgICAgICAgICAgICAgICAgICAgc2NhbGVfdHJhbnMgPSBOVUxMKSB7CiAgcGx0IDwtIGdncGxvdChhc3NheV9kYXRhLAogICAgICAgICAgICAgICAgYWVzX3N0cmluZygKICAgICAgICAgICAgICAgICAgeCA9IHBhc3RlMCgndW5saXN0KGAnLCBhbm5vdGF0aW9uLCAnYCknKSwKICAgICAgICAgICAgICAgICAgZmlsbCA9IGZpbGxfYnkpCiAgICAgICAgICAgICAgICApICsKICAgIGdlb21faGlzdG9ncmFtKGJpbnMgPSAxMDApICsKICAgIGdlb21fdmxpbmUoCiAgICAgIHhpbnRlcmNlcHQgPSB0aHIsCiAgICAgIGx0eSA9ICdkYXNoZWQnLAogICAgICBjb2xvciA9ICdibGFjaycKICAgICAgKSArCiAgICB0aGVtZV9idygpICsKICAgIGd1aWRlcyhmaWxsID0gJ25vbmUnKSArCiAgICBmYWNldF93cmFwKAogICAgICBhcy5mb3JtdWxhKHBhc3RlKCd+JywgZmlsbF9ieSkpLAogICAgICBucm93ID0gNCwKICAgICAgc2NhbGVzID0gJ2ZyZWVfeScKICAgICAgKSArCiAgICBzY2FsZV9maWxsX21hbnVhbCh2YWx1ZXMgPSBhbm5fY29sb3JzW1tmaWxsX2J5XV0pICsKICAgIGxhYnMoCiAgICAgIHRpdGxlID0gYW5ub3RhdGlvbiwKICAgICAgeCA9IGFubm90YXRpb24sCiAgICAgIHkgPSAnIyBzZWdtZW50cycKICAgICAgKQogIGlmKCFpcy5udWxsKHNjYWxlX3RyYW5zKSkgewogICAgcGx0IDwtIHBsdCArCiAgICAgIHNjYWxlX3hfY29udGludW91cyh0cmFucyA9IHNjYWxlX3RyYW5zKQogIH0KICBwbHQKfQpgYGAKCiMjIyMgVHJpbW1lZAoKYGBge3J9ClFDX2hpc3RvZ3JhbShzRGF0YShEYXRhKSwgJ1RyaW1tZWQgKCUpJywgY29sX2J5LCBzZWdtZW50UUNfcGFyYW1zJHBlcmNlbnRUcmltbWVkKSArCiAgc2NhbGVfeF9jb250aW51b3VzKAogICAgbGltaXRzID0gYyg4MCwxMDApLAogICAgYnJlYWtzID0gYyg4NSwgOTAsIDk1LCAxMDAsIHNlZ21lbnRRQ19wYXJhbXMkcGVyY2VudFRyaW1tZWQpCiAgKQpgYGAKCiMjIyMgU3RpY2hlZCAoJSkKCmBgYHtyfQpRQ19oaXN0b2dyYW0oc0RhdGEoRGF0YSksICdTdGl0Y2hlZCAoJSknLCBjb2xfYnksIHNlZ21lbnRRQ19wYXJhbXMkcGVyY2VudFN0aXRjaGVkKSArCiAgc2NhbGVfeF9jb250aW51b3VzKAogICAgbGltaXRzID0gYygwLDEwMCksCiAgICBicmVha3MgPSBjKDAsIDI1LCA1MCwgNzUsIDEwMCwgc2VnbWVudFFDX3BhcmFtcyRwZXJjZW50U3RpdGNoZWQpCiAgKQpgYGAKCiMjIyMgQWxpZ25lZCAoJSkKCmBgYHtyfQpRQ19oaXN0b2dyYW0oc0RhdGEoRGF0YSksICdBbGlnbmVkICglKScsIGNvbF9ieSxzZWdtZW50UUNfcGFyYW1zJHBlcmNlbnRBbGlnbmVkKSArCiAgc2NhbGVfeF9jb250aW51b3VzKAogICAgbGltaXRzID0gYygwLDEwMCksCiAgICBicmVha3MgPSBjKDAsIDI1LCA1MCwgNzUsIDEwMCwgc2VnbWVudFFDX3BhcmFtcyRwZXJjZW50QWxpZ25lZCkKICApCmBgYAoKIyMjIyBTZXF1ZW5jaW5nIFNhdHVyYXRpb24gKCUpIHsuYWN0aXZlfQoKYGBge3J9ClFDX2hpc3RvZ3JhbShzRGF0YShEYXRhKSwgJ1NhdHVyYXRlZCAoJSknLCBjb2xfYnksIHNlZ21lbnRRQ19wYXJhbXMkcGVyY2VudFNhdHVyYXRpb24pICsKICBsYWJzKAogICAgdGl0bGUgPSAnU2VxdWVuY2luZyBTYXR1cmF0aW9uICglKScsCiAgICB4ID0gJ1NlcXVlbmNpbmcgU2F0dXJhdGlvbiAoJSknCiAgICApICsKICBzY2FsZV94X2NvbnRpbnVvdXMoCiAgICBsaW1pdHMgPSBjKDAsMTAwKSwKICAgIGJyZWFrcyA9IGMoMCwgMjUsIDUwLCA3NSwgMTAwLCBzZWdtZW50UUNfcGFyYW1zJHBlcmNlbnRTYXR1cmF0aW9uKQogICkKYGBgCgojIyMjIEFyZWEKCmBgYHtyfQpRQ19oaXN0b2dyYW0oc0RhdGEoRGF0YSksICdhcmVhJywgY29sX2J5LCBzZWdtZW50UUNfcGFyYW1zJG1pbkFyZWEsIHNjYWxlX3RyYW5zID0gJ2xvZzEwJykgKwogICAgbGFicygKICAgIHRpdGxlID0gJ0FyZWEnLAogICAgeCA9ICdBcmVhJwogICAgKSArCiAgc2NhbGVfeF9jb250aW51b3VzKAogICAgbGltaXRzID0gYygxMDAwLDIwMDAwMCksCiAgICBicmVha3MgPSBjKDEwMDAsIDEwMDAwLCAxMDAwMDAsIHNlZ21lbnRRQ19wYXJhbXMkbWluQXJlYSksCiAgICB0cmFucyA9ICdsb2cxMCcKICApCmBgYAoKIyMjIyBOdWNsZWkgY291bnQKCmBgYHtyfQpRQ19oaXN0b2dyYW0oc0RhdGEoRGF0YSksICdudWNsZWknLCBjb2xfYnksIHNlZ21lbnRRQ19wYXJhbXMkbWluTnVjbGVpKSArCiAgICBsYWJzKAogICAgdGl0bGUgPSAnIyBOdWNsZWknLAogICAgeCA9ICcjIE51Y2xlaScKICAgICkgKwogIHNjYWxlX3hfY29udGludW91cygKICAgIGxpbWl0cyA9IGMoMCwxNTAwKSwKICAgIGJyZWFrcyA9IGMoMCwgMjUwLCA1MDAsIDc1MCwgMTAwMCwgMTI1MCwgMTUwMCwgc2VnbWVudFFDX3BhcmFtcyRtaW5OdWNsZWkpCiAgKQpgYGAKCiMjIyMgRHVwbGljYXRpb25SYXRlOiBEZWR1cCAvIFJhdwoKYGBge3J9CmdncGxvdChwRGF0YShwcm90b2NvbERhdGEoRGF0YSkpLAogICAgICAgYWVzKAogICAgICAgICB4ID0gUGxhdGVfSUQsCiAgICAgICAgIHkgPSAoRGVkdXBsaWNhdGVkUmVhZHMvUmF3KSkKICAgICAgICAgKSArCiAgZ2VvbV92aW9saW4oYWVzKGZpbGwgPSBQbGF0ZV9JRCkpICsKICBnZW9tX2ppdHRlcih3aWR0aCA9IDAuMikgKwogIGxhYnMoeSA9ICdEZWR1cGxpY2F0ZWQgLyBSYXcgcmVhZHMnKSArCiAgc2NhbGVfeV9jb250aW51b3VzKGxhYmVscyA9IHNjYWxlczo6cGVyY2VudCkgKwogIHRoZW1lX2J3KCkgKwogICAgdGhlbWUoCiAgICBheGlzLnRpdGxlLnggPSBlbGVtZW50X2JsYW5rKCksCiAgICBheGlzLnRleHQueCA9IGVsZW1lbnRfYmxhbmsoKSwKICAgIGF4aXMudGlja3MueCA9IGVsZW1lbnRfYmxhbmsoKQogICkKYGBgCgojIyMjIERldXBsaWNhdGlvblJhdGU6IERlZHVwIC8gQWxpZ25lZAoKYGBge3J9CmdncGxvdChwRGF0YShwcm90b2NvbERhdGEoRGF0YSkpLAogICAgICAgYWVzKAogICAgICAgICB4ID0gUGxhdGVfSUQsCiAgICAgICAgIHkgPSAoRGVkdXBsaWNhdGVkUmVhZHMvQWxpZ25lZCkpCiAgICAgICAgICkgKwogIGdlb21fdmlvbGluKGFlcyhmaWxsID0gUGxhdGVfSUQpKSArCiAgZ2VvbV9qaXR0ZXIod2lkdGggPSAwLjIpICsKICBsYWJzKHkgPSAnRGVkdXBsaWNhdGVkIC8gQWxpZ25lZCByZWFkcycpICsKICBzY2FsZV95X2NvbnRpbnVvdXMobGFiZWxzID0gc2NhbGVzOjpwZXJjZW50KSArCiAgdGhlbWVfYncoKSArCiAgdGhlbWUoCiAgICBheGlzLnRpdGxlLnggPSBlbGVtZW50X2JsYW5rKCksCiAgICBheGlzLnRleHQueCA9IGVsZW1lbnRfYmxhbmsoKSwKICAgIGF4aXMudGlja3MueCA9IGVsZW1lbnRfYmxhbmsoKQogICkKYGBgCgojIyMjIE5lZ3Byb2JlcyB2cyBFbmRvZ2Vub3VzCgpgYGB7ciBwbG90X25lZ3Byb2JlX2RhdGEsIGZpZy53aWR0aD0xMCxmaWcuaGVpZ2h0PTV9CnRtcF90YXJnZXRfRGF0YSA8LSBhZ2dyZWdhdGVDb3VudHMoRGF0YSkKCiMgZ2V0IG5lZ2F0aXZlIHByb2JlIGRhdGEKdG1wX25lZ3MgPC0gc3Vic2V0KHRtcF90YXJnZXRfRGF0YSwgQ29kZUNsYXNzID09ICdOZWdhdGl2ZScpCgpwMSA8LSBnZ3Bsb3QocERhdGEodG1wX25lZ3MpLAogICAgICAgICAgICAgYWVzKAogICAgICAgICAgICAgICB4ID0gc2VnbWVudCwKICAgICAgICAgICAgICAgeSA9IGFzc2F5RGF0YUVsZW1lbnQodG1wX25lZ3MsIGVsdCA9ICdleHBycycpKSkgKwogIGdlb21fdmlvbGluKGFlcyhmaWxsID0gc2VnbWVudCkpICsKICBnZW9tX2ppdHRlcih3aWR0aCA9IDAuMikgKwogIGNvb3JkX2ZsaXAoKSArCiAgbGFicyh5ID0gJ05lZ2F0aXZlIHByb2JlcyBleHByZXNzaW9uJykgKwogIHNjYWxlX2ZpbGxfbWFudWFsKHZhbHVlcyA9IGFubl9jb2xvcnMkc2VnbWVudCkgKwogIHNjYWxlX3lfY29udGludW91cyhsaW1pdHMgPSBjKDEsMzAwMCksIHRyYW5zID0gJ2xvZzInKSArCiAgdGhlbWVfYncoKQoKIyBnZXQgZW5kb2dlbm91cyBwcm9iZSBkYXRhCnRtcF9lbmQgPC0gc3Vic2V0KHRtcF90YXJnZXRfRGF0YSwgQ29kZUNsYXNzID09ICdFbmRvZ2Vub3VzJykKCnAyIDwtIGdncGxvdChwRGF0YSh0bXBfZW5kKSwKICAgICAgICAgICAgIGFlcygKICAgICAgICAgICAgICAgeCA9IHNlZ21lbnQsCiAgICAgICAgICAgICAgIHkgPSBjb2xNZWFucyhhc3NheURhdGFFbGVtZW50KHRtcF9lbmQsIGVsdCA9ICdleHBycycpKSkpICsKICBnZW9tX3Zpb2xpbihhZXMoZmlsbCA9IHNlZ21lbnQpKSArCiAgZ2VvbV9qaXR0ZXIod2lkdGggPSAwLjIpICsKICBjb29yZF9mbGlwKCkgKwogIGxhYnMoeSA9ICdFbmRvZ2Vub3VzIHByb2JlcyBleHByZXNzaW9uIChtZWFuKScpICsKICBzY2FsZV9maWxsX21hbnVhbCh2YWx1ZXMgPSBhbm5fY29sb3JzJHNlZ21lbnQpICsKICBzY2FsZV95X2NvbnRpbnVvdXMobGltaXRzID0gYygxLDMwMDApLCB0cmFucyA9ICdsb2cyJykgKwogIHRoZW1lX2J3KCkKCnBsIDwtIGxpc3QocDEsIHAyKQoKcGxvdF9ncmlkKHBsb3RsaXN0ID0gcGwsIG5yb3cgPSAyLCBhbGlnbiA9ICd2JykKYGBgCgojIyMjIE5lZ19wcm9iZSByZWFkcyBjb21wYXJlZCB0byByYXdfcmVhZHMKCmBgYHtyfQojIG1ha2UgYmFja2dyb3VuZCB0b3RhbCBuZWcgcHJvYmUgY291bnQKdG1wX2ZkYXRhX2RmIDwtIGZEYXRhKERhdGEpCm5lZ3Byb2Jlc25hbWVzIDwtIHJvd25hbWVzKHRtcF9mZGF0YV9kZlt0bXBfZmRhdGFfZGYkTmVnYXRpdmUgPT0gVFJVRSxdKQp0bXBfZXhwIDwtIGFzc2F5RGF0YUVsZW1lbnQoRGF0YSwgZWx0ID0gJ2V4cHJzJykKbmVncHJvYmVfZXhwcl9mZCA8LSB0bXBfZXhwW3Jvd25hbWVzKHRtcF9leHApICVpbiUgbmVncHJvYmVzbmFtZXMsIF0KdG90X25lZ19jdHJsX3JlYWRzIDwtIGNvbFN1bXMobmVncHJvYmVfZXhwcl9mZCkKdG90X2RlZHVwX3JlYWRzIDwtIHBEYXRhKHByb3RvY29sRGF0YShEYXRhKSkkRGVkdXBsaWNhdGVkUmVhZHMKCnRtcF9uZWdkZWR1cHJlYWRzX2RmIDwtIGRhdGEuZnJhbWUoJ2FvaScgPSBuYW1lcyh0b3RfbmVnX2N0cmxfcmVhZHMpLAogICAgICAgICAgICAgICAgICAgICAgICAgICAgICAgICAgICd0b3RfZGVkdXBfcmVhZHMnID0gYXMubnVtZXJpYyh0b3RfZGVkdXBfcmVhZHMpLAogICAgICAgICAgICAgICAgICAgICAgICAgICAgICAgICAgICd0b3RfbmVnX2N0cmxfcmVhZHMnID0gYXMubnVtZXJpYyh0b3RfbmVnX2N0cmxfcmVhZHMpCikKCnRtcF9uZWdkZWR1cHJlYWRzX2RmIDwtIG1lbHQodG1wX25lZ2RlZHVwcmVhZHNfZGYsIGlkID0gJ2FvaScpCgpnZ3Bsb3QodG1wX25lZ2RlZHVwcmVhZHNfZGYsCiAgICAgICBhZXMoCiAgICAgICAgIHggPSBhb2ksCiAgICAgICAgIHkgPSB2YWx1ZSwKICAgICAgICAgZmlsbCA9IHZhcmlhYmxlKSkgKwogIGdlb21fYmFyKHBvc2l0aW9uID0gJ2lkZW50aXR5Jywgc3RhdCA9ICdpZGVudGl0eScpICsKICBzY2FsZV95X2NvbnRpbnVvdXModHJhbnMgPSBsb2cyX3RyYW5zKCkpICsKICBsYWJzKAogICAgeCA9ICdBT0knLAogICAgeSA9ICdOdW1iZXIgb2YgcmVhZHMnLAogICAgZmlsbCA9ICcnCiAgICApICsKICB0aGVtZSgKICAgIGxlZ2VuZC5wb3NpdGlvbiA9ICdib3R0b20nLAogICAgYXhpcy50ZXh0LnggPSBlbGVtZW50X2JsYW5rKCksCiAgICBheGlzLnRpY2tzLnggPSBlbGVtZW50X2JsYW5rKCkKICAgICkKYGBgCgojIyMjIER1cGxpY2F0ZWQgcmVhZHMgdnMgQmFja2dyb3VuZAoKYGBge3J9CiMgZ2V0IGRjYyBwZXIgcGxhdGUuIHN1bSBuZWdwcm9iZSBjb3VudHMvZGNjL3BsYXRlCmdncGxvdChwRGF0YShwcm90b2NvbERhdGEoRGF0YSkpLAogICAgICAgYWVzKAogICAgICAgICB4ID0gUGxhdGVfSUQsCiAgICAgICAgIHkgPSBEZWR1cGxpY2F0ZWRSZWFkcywKICAgICAgICAgZmlsbCA9IFBsYXRlX0lEKSkgKwogIGdlb21fdmlvbGluKCkgKwogIGdlb21faml0dGVyKHdpZHRoID0gMC4yKSArCiAgbGFicyh5ID0gJ0RlZHVwbGljYXRlZCAvIFJhdyByZWFkcycpICsKICBzY2FsZV95X2xvZzEwKCkgKwogIGdlb21faGxpbmUoZGF0YSA9IHBEYXRhKHByb3RvY29sRGF0YShEYXRhKSksCiAgICAgICAgICAgICBhZXMoCiAgICAgICAgICAgICAgIHlpbnRlcmNlcHQgPSBOVEMsCiAgICAgICAgICAgICAgIGNvbG91ciA9IFBsYXRlX0lECiAgICAgICAgICAgICAgICkpICsKICB0aGVtZV9idygpCmBgYAoKIyMjIyBEdXBsaWNhdGVkIHJlYWRzIHZzIFJPSWFyZWEKCmBgYHtyLCBmaWcud2lkdGg9MTUsZmlnLmhlaWdodD01fQp0bXBfZGYgPC0gY2JpbmQocERhdGEoRGF0YSksCiAgICAgICAgICAgICAgICAgcERhdGEocHJvdG9jb2xEYXRhKERhdGEpKSwKICAgICAgICAgICAgICAgICBkY2M9cm93bmFtZXMocERhdGEoRGF0YSkpKQoKZ2dwbG90KHRtcF9kZiAlPiUgZmlsdGVyKChEZWR1cGxpY2F0ZWRSZWFkcy9hcmVhKSA8PSA1KSwKICAgICAgIGFlcygKICAgICAgICAgeCA9IGRjYywKICAgICAgICAgeSA9IChEZWR1cGxpY2F0ZWRSZWFkcy9hcmVhKSwKICAgICAgICAgY29sb3VyID1gc2xpZGUgbmFtZWApKSArCiAgZ2VvbV9wb2ludChzaXplID0gNCwgcG9zaXRpb24gPSBwb3NpdGlvbl9kb2RnZSh3aWR0aCA9IDEpKSArCiAgc2NhbGVfeF9kaXNjcmV0ZShleHBhbmQgPSBleHBhbmRfc2NhbGUoYWRkID0gYygxLDEpKSkrCiAgc2NhbGVfeV9jb250aW51b3VzKGxpbWl0cyA9IGMoMCw1KSkgKwogIGxhYnMoeSA9ICdEZWR1cGxpY2F0ZWQgcmVhZHMgLyBST0kgYXJlYScpICsKICB0aGVtZSgKICAgIHBsb3QubWFyZ2luID0gdW5pdChjKDEsMSwxLDYpLCAnY20nKSwKICAgIGF4aXMudGV4dCA9IGVsZW1lbnRfdGV4dChzaXplID0gNiksCiAgICBheGlzLnRleHQueCA9IGVsZW1lbnRfdGV4dChhbmdsZSA9IDQ1LCBoanVzdCA9IDEpCiAgICApCmBgYAoKIyMjIyBEdXBsaWNhdGVkIHJlYWRzIHZzIG51Y2xlaQoKYGBge3IsIGZpZy53aWR0aD0xNSxmaWcuaGVpZ2h0PTV9CnRtcF9kZiA8LSBjYmluZChwRGF0YShEYXRhKSwKICAgICAgICAgICAgICAgICBwRGF0YShwcm90b2NvbERhdGEoRGF0YSkpLAogICAgICAgICAgICAgICAgIGRjYz1yb3duYW1lcyhwRGF0YShEYXRhKSkpCgpnZ3Bsb3QodG1wX2RmICU+JSBmaWx0ZXIoKERlZHVwbGljYXRlZFJlYWRzL251Y2xlaSkgPCAzMDAwKSwKICAgICAgIGFlcygKICAgICAgICAgeCA9IGRjYywKICAgICAgICAgeSA9IChEZWR1cGxpY2F0ZWRSZWFkcy9udWNsZWkpLAogICAgICAgICBjb2xvdXIgPSBgc2xpZGUgbmFtZWAKICAgICAgICAgKSkgKwogIGdlb21fcG9pbnQoc2l6ZSA9IDQsIHBvc2l0aW9uID0gcG9zaXRpb25fZG9kZ2Uod2lkdGggPSAxKSkgKwogIHNjYWxlX3hfZGlzY3JldGUoZXhwYW5kID0gZXhwYW5kX3NjYWxlKGFkZCA9IGMoMSwxKSkpKwogIHNjYWxlX3lfY29udGludW91cyhsaW1pdHMgPSBjKDAsMzAwMCkpICsKICBsYWJzKHkgPSAnRGVkdXBsaWNhdGVkIHJlYWRzIC8gbnVjbGVpJykgKwogIHRoZW1lKAogICAgcGxvdC5tYXJnaW4gPSB1bml0KGMoMSwxLDEsMiksICdjbScpLAogICAgYXhpcy50ZXh0ID0gZWxlbWVudF90ZXh0KHNpemUgPSA2KSwKICAgIGF4aXMudGV4dC54ID0gZWxlbWVudF90ZXh0KGFuZ2xlID0gNDUsIGhqdXN0ID0gMSkKICAgICkKYGBgCgojIyMgNC4xLjMgUHJvY2VzcyBOZWdhdGl2ZSBHZW9NZWFucwoKYGBge3J9CiMgY2FsY3VsYXRlIHRoZSBuZWdhdGl2ZSBnZW9tZXRyaWMgbWVhbnMgZm9yIGVhY2ggbW9kdWxlCm5lZ2F0aXZlR2VvTWVhbnMgPC0gZXNCeShuZWdhdGl2ZUNvbnRyb2xTdWJzZXQoRGF0YSksCiAgICAgICAgICAgICAgICAgICAgICAgICBHUk9VUCA9ICdNb2R1bGUnLAogICAgICAgICAgICAgICAgICAgICAgICAgRlVOID0gZnVuY3Rpb24oeCkgewogICAgICAgICAgICAgICAgICAgICAgICAgICBhc3NheURhdGFBcHBseSh4LCBNQVJHSU4gPSAyLCBGVU4gPSBuZ2VvTWVhbiwgZWx0ID0gJ2V4cHJzJykKICAgICAgICAgICAgICAgICAgICAgICAgICAgfSkKcHJvdG9jb2xEYXRhKERhdGEpW1snTmVnR2VvTWVhbiddXSA8LSBuZWdhdGl2ZUdlb01lYW5zCm5lZ0NvbHMgPC0gcGFzdGUwKCdOZWdHZW9NZWFuXycsIG1vZHVsZXMpCnBEYXRhKERhdGEpWywgbmVnQ29sc10gPC0gc0RhdGEoRGF0YSlbWydOZWdHZW9NZWFuJ11dCgpmb3IoYW5uIGluIG5lZ0NvbHMpIHsKICBwbHQgPC0gUUNfaGlzdG9ncmFtKHBEYXRhKERhdGEpLCBhbm4sIGNvbF9ieSwgMTAsIHNjYWxlX3RyYW5zID0gJ2xvZzEwJykKICBwcmludChwbHQpCn0KCiMgRGV0YXRjaCBuZWdfZ2VvbWVhbiBjb2x1bW5zIGFoZWFkIG9mIGFnZ3JlZ2F0ZUNvdW50cyBjYWxsCnBEYXRhKERhdGEpIDwtIHBEYXRhKERhdGEpWywgIWNvbG5hbWVzKHBEYXRhKERhdGEpKSAlaW4lIG5lZ0NvbHNdCmBgYAoKU2hvdyBhbGwgTlRDIHZhbHVlcywgRnJlcSA9IFwjIG9mIHNlZ21lbnRzIHdpdGggYSBnaXZlbiBOVEMgY291bnQ6CgpgYGB7ciBRQ190YWJsZXN9CnRtcF9zZWdtZW50UUMgPC0gc0RhdGEoRGF0YSkKCiMgY2FsY3VsYXRlIGhvdyBtYW55IGZsYWdzIGEgUk9JIHJlY2VpdmVkCnRtcF9zZWdtZW50UUMgPC0gdG1wX3NlZ21lbnRRQyAlPiUKICBtdXRhdGUoCiAgICBRQ1N0YXR1cyA9IGlmX2Vsc2Uocm93U3VtcyhhY3Jvc3MoUUNGbGFncykpID09IDBMLCAnUEFTUycsICdXQVJOSU5HJyksCiAgICBuRmxhZ3MgPSByb3dTdW1zKGFjcm9zcyhRQ0ZsYWdzKSksIC5hZnRlciA9IFFDRmxhZ3MKICAgICkKCnRtcF9udGNfZGYgPC0gdG1wX3NlZ21lbnRRQ1ssYygnc2xpZGUgbmFtZScsICdQbGF0ZV9JRCcsICdOVENfSUQnLCAnTlRDJyldCnRtcF9udGNfZGYgPC0gdG1wX250Y19kZiAlPiUgZHBseXI6OmNvdW50KHRtcF9udGNfZGYkJ3NsaWRlIG5hbWUnLCB0bXBfbnRjX2RmJE5UQ19JRCwgdG1wX250Y19kZiRQbGF0ZV9JRCwgdG1wX250Y19kZiROVEMpCmNvbG5hbWVzKHRtcF9udGNfZGYpIDwtIGMoJ1NsaWRlIG5hbWUnLCAnTlRDX0lEJywgJ1BsYXRlX0lEJywKICAgICAgICAgICAgICAgICAgICAgICAgICdOVEMgY291bnQnLCAnTnVtYmVyIG9mIHNhbXBsZXMnKQoKZGF0YXRhYmxlKHRtcF9udGNfZGYsIHJvd25hbWVzID0gRkFMU0UpCgpkYXRhdGFibGUoc2VnbWVudFFDX1N1bW1hcnksCiAgICAgICAgICBjYXB0aW9uID0gJ0FPSSBRQyBTdW1tYXJ5JywKICAgICAgICAgIGV4dGVuc2lvbnMgPSAnQnV0dG9ucycsCiAgICAgICAgICBvcHRpb25zID0gbGlzdCAoCiAgICAgICAgICAgIGRvbSA9ICdCZnRyaXAnLAogICAgICAgICAgICBidXR0b25zID0gYygnY29weScsICdjc3YnLCAnZXhjZWwnLCAncGRmJywgJ3ByaW50JykKICAgICAgICAgICkKKQpgYGAKCiMjIyBBT0lzIHRoYXQgZmFpbCBRQwoKU2hvdyBBT0lzIHdoaWNoIGZhaWwgY3JpdGljYWwgUUNzLgoKYGBge3IgbGlzdF9mYWlsdXJlc30KcWNfY29sX2xpc3QgPC0gYXMubGlzdChjb2xuYW1lcyh0bXBfc2VnbWVudFFDKSkKbmFtZXMocWNfY29sX2xpc3QpIDwtIGNvbG5hbWVzKHRtcF9zZWdtZW50UUMpCmZhaWxlZF9xY19zZWdtZW50c19hbGwgPC0gdG1wX3NlZ21lbnRRQ1t0bXBfc2VnbWVudFFDW3FjX2NvbF9saXN0JFFDU3RhdHVzXSA9PSAnV0FSTklORycsXSAjIGFsbCBmbGFnZ2VkIHNlZ21lbnRzCgojIHB1bGwgbmVzdGVkIFFDRmxhZ3MgZGYgb3V0IG9mIG9iamVjdCAoaXQgd2lsbCBtZXNzIHVwIHRoZSBkYXRhdGFibGUgb3RoZXJ3aXNlKTsgdGlkeSB0aGUgdGFibGUKZmFpbGVkX3FjX3NlZ21lbnRzX2FsbDEgPC0gY2JpbmQoZmFpbGVkX3FjX3NlZ21lbnRzX2FsbCwgZmFpbGVkX3FjX3NlZ21lbnRzX2FsbCAlPiUgcHVsbChRQ0ZsYWdzKSkgJT4lCiAgIyByZW1vdmUgdGhlIG5lc3RlZCBkZnMsIHRoZXkgaW50ZXJmZXJlIHdpdGggdGhlIGdlbmVyYXRpb24gb2YgYSBUU1YKICBzZWxlY3QoLWMoYFRyaW1tZWQgKCUpYDpOZWdHZW9NZWFuKSwgLVFDRmxhZ3MpCgojIHB1bGwgbmVzdGVkIFFDIHBlcmNlbnRhZ2VzIGFuZCBOZWdHZW9NZWFuIG5lc3RlZCBkZnMgYW5kIGJyaW5nIHRoZW0gdG8gdGhlIHNhbWUgbGV2ZWwgYXMgdGhlIG90aGVyIGNvbHVtbnMgdG8gd3JpdGUgdGhlIHRhYmxlIHRvIGRpc2sKZmFpbGVkX3FjX3NlZ21lbnRzX2FsbDEgPC0gY2JpbmQoZmFpbGVkX3FjX3NlZ21lbnRzX2FsbDEsCiAgICAgICAgICAgICAgICAgICAgICAgICAgICAgICAgIGZhaWxlZF9xY19zZWdtZW50c19hbGwgJT4lIHB1bGwoYFRyaW1tZWQgKCUpYCksCiAgICAgICAgICAgICAgICAgICAgICAgICAgICAgICAgIGZhaWxlZF9xY19zZWdtZW50c19hbGwgJT4lIHB1bGwoYFN0aXRjaGVkICglKWApLAogICAgICAgICAgICAgICAgICAgICAgICAgICAgICAgICBmYWlsZWRfcWNfc2VnbWVudHNfYWxsICU+JSBwdWxsKGBBbGlnbmVkICglKWApLAogICAgICAgICAgICAgICAgICAgICAgICAgICAgICAgICBmYWlsZWRfcWNfc2VnbWVudHNfYWxsICU+JSBwdWxsKGBTYXR1cmF0ZWQgKCUpYCksCiAgICAgICAgICAgICAgICAgICAgICAgICAgICAgICAgIGZhaWxlZF9xY19zZWdtZW50c19hbGwgJT4lIHB1bGwoTmVnR2VvTWVhbikpICU+JQogIHNlbGVjdChTYW1wbGVJRCwgYHNsaWRlIG5hbWVgLCBQYXRpZW50X0lELCByb2ksIHNlZ21lbnQsIHNsaWRlTG9jYXRpb24sIHRpc3N1ZVJlZ2lvbiwKICAgICAgICAgUUNTdGF0dXMsIG5GbGFncywgYXJlYSwgbnVjbGVpLCB1bWlRMzAsIHJ0c1EzMCwKICAgICAgICAgUmF3OkFsaWduZWQsIERlZHVwbGljYXRlZFJlYWRzLCBgVHJpbW1lZCAoJSlgOkFsaWduZWQsIExvd1JlYWRzOkxvd0FyZWEsIE5UQ19JRCwgTlRDKSAlPiUKICAjIHJlbmFtZSBmb3IgYmV0dGVyIHJlYWRhYmlsaXR5CiAgcmVuYW1lKAogICAgU2FtcGxlSUQgPSAnU2FtcGxlX0lEJwogICkgJT4lCiAgYXJyYW5nZShgc2xpZGUgbmFtZWAsIFBhdGllbnRfSUQsIHJvaSkKCiMgd3JpdGUgdGhpcyB0YWJsZSB0byBkaXNrCnFjX2RpciA8LSBwYXN0ZTAob3V0cHV0X2RpciwgJy8nLCBpbnB1dF9leHBlcmltZW50LCAnL291dHB1dC9xY19yZXN1bHRzLycsIGN1cnJlbnRfZGF0ZSkKaWYgKCFkaXIuZXhpc3RzKHFjX2RpcikpIHsKICBkaXIuY3JlYXRlKHFjX2RpciwgcmVjdXJzaXZlID0gVFJVRSkKfQp3cml0ZV90c3YoCiAgZmFpbGVkX3FjX3NlZ21lbnRzX2FsbDEsCiAgZmlsZSA9IHBhc3RlMChxY19kaXIsICcvZmFpbGVkX3FjX3NlZ21lbnRzLnRzdicpCikgCgojIGFsc28gcHJpbnQgdGhlIHRhYmxlCmRhdGF0YWJsZShmYWlsZWRfcWNfc2VnbWVudHNfYWxsMSwKICAgICAgICAgIHJvd25hbWVzID0gRkFMU0UsCiAgICAgICAgICBleHRlbnNpb25zID0gJ0J1dHRvbnMnLCBvcHRpb25zID0gbGlzdCAoCiAgICAgICAgICAgIGRvbSA9ICdCZnRyaXAnLAogICAgICAgICAgICBidXR0b25zID0gYygnY29weScsICdjc3YnLCAnZXhjZWwnLCAncGRmJywgJ3ByaW50JykKICAgICAgICAgICkKKQpgYGAKClRoZXJlIGFyZSBgciBucm93KGZhaWxlZF9xY19zZWdtZW50c19hbGwxKWAgc2VnbWVudHMgd2hpY2ggZmFpbGVkIGF0CmxlYXN0IG9uZSBRQyBtZWFzdXJlLiBUaGlzIHRhYmxlIHdhcyBhc3Nlc3NlZCBtYW51YWxseSBmb3Igd2hldGhlciBzZWdtZW50cyB3ZXJlCnN0aWxsIGFjY2VwdGFibGUgdG8gYmUgaW5jbHVkZWQgaW4gZG93bnN0cmVhbSBhbmFseXNpcy4gTWFudWFsbHkKYXNzZXNzZWQgc2VnbWVudHMgd2VyZSBwdXQgaW50byBvbmUgb2YgdGhyZWUgY2F0ZWdvcmllczo8YnI+IAotICoqSU5DTFVERToqKiBzZWdtZW50IHNob3dlZCBlbm91Z2ggcXVhbGl0eSB0byBiZSBpbmNsdWRlZCBldmVuIGFmdGVyCmZsYWdnaW5nLiBUaGVyZSBjb3VsZCBiZSBzZXZlcmFsIHJlYXNvbnMgZm9yIHRoaXMuIEZvciBleGFtcGxlLCBpdApzY29yZWQganVzdCBiZWxvdyB0aGUgdGhyZXNob2xkIGZvciBvbmUgcGFyYW1ldGVyLCBidXQgaGFkIGdvb2QKcXVhbGl0aWVzIG92ZXJhbGwuIE9yLCBpdCB3YXMgZmxhZ2dlZCBkdWUgdG8gUUMgbWVhc3VyZXMgd2hpY2ggYXJlCmxhcmdlbHkgc3R1ZHkgZGVwZW5kZW50IGFuZCBoYXZlIGEgbG90IG9mIGxlZXdheSAoZS5nLiBudW1iZXIgb2YKbnVjbGVpKS48YnI+Ci0gKipDT05TSURFUjoqKiBzZWdtZW50IHNob3dlZCBkaWRuJ3Qgc2hvdyBlbm91Z2ggcXVhbGl0eSB0byBiZQppbmNsdWRlZCwgYnV0IGlzIGFsc28gbm90IG9mIHdvcnNlIGVub3VnaCBxdWFsaXR5IHRvIGJlIGNvbXBsZXRlbHkKcmVtb3ZlZC4gVGhlIHJlYXNvbnMgZm9yIHRoaXMgYXJlIGRpZmZlcmVudCBhbmQgbW9zdGx5IHRoZSBpbnRlcnBsYXkgb2YKbXV0bGlwbGUgcXVhbGl0eSBtZWFzdXJlcyB3ZXJlIGNvbnNpZGVyZWQgZm9yIHRoaXMgZGVjaXNpb24uIEl0IGNvdWxkCmVpdGhlciBiZSB0aGF0IG9uZSBxdWFsaXR5IG1lYXN1cmUgd2FzIHN1YnBhciwgYnV0IG90aGVycyB3ZXJlIGFtYXppbmcuCk9yLCBtdWx0aXBsZSBxdWFsaXR5IG1lYXN1cmVzIHdlcmUgZm91bmQgdG8gYmUganVzdCBiZWxvdyB0aHJlc2hvbGQuClRoaXMgY2F0ZWdvcnkgcHJvdmlkZXMgcG90ZW50aWFsIHNlZ21lbnRzIHRoYXQgY291bGQgYmUgaW5jbHVkZWQgZnVydGhlcgpkb3duc3RyZWFtLCBidXQgaGF2ZSB0byBiZSBtb25pdG9yZWQgY2xvc2VseS48YnI+Ci0gKipSRU1PVkU6KiogVGhlIHNlZ21lbnQKc2hvd2VkIHZlcnkgYmFkIHF1YWxpdHkgZm9yIG9uZSBvciBldmVuIG11bHRpcGxlIFFDIG1lYXN1cmVzLjxicj4gRXZlcnkKZGVjaXNpb24gaXMgYWNjb21wYW5pZWQgd2l0aCBhIGNvbW1lbnQgdGhhdCBjYW4gYmUgZm91bmQgaW4gdGhlIGNvbHVtbgoqQXNzZXNzbWVudENvbW1lbnQqLiBSZWFkIHRoZSBtYW51YWxseSBhc3Nlc3NlZCBzZWdtZW50IFFDIHRhYmxlIGludG8gUgphZ2Fpbi48YnI+IEFmdGVyIG1hbnVhbCBhc3Nlc3NtZW50IG9mIHRoZSBzZWdtZW50cyB0aGF0IHdlcmUgZmxhZ2dlZCBhcwpzdWJwYXIgYWNjb3JkaW5nIHRvIHRoZSBkZWZhdWx0IFFDIHBhcmFtZXRlcnMsIG9ubHQgdGhvc2UgdGhhdCB3ZXJlCmZsYWdnZWQgKipJTkNMVURFRCoqIHdlcmUgaW5jbHVkZWQuCgpgYGB7ciwgaW5jbHVkZT1UUlVFLCBldmFsPVRSVUV9CiMgZmFpbGVkX3FjX2Fzc2Vzc2VkX2ZpbGUgPC0gcGFzdGUwKG91dHB1dF9kaXIsICcvJywgaW5wdXRfZXhwZXJpbWVudCwgJy9vdXRwdXQvcWNfcmVzdWx0cy8nLCBxY19hc3Nlc3NtZW50X2RhdGUsICcvZmFpbGVkX3FjX3NlZ21lbnRzX2Fzc2Vzc2VkLmNzdicpCmBgYAoKYGBge3IsIG1lc3NhZ2U9RkFMU0UsIHdhcm5pbmc9RkFMU0UsIGluY2x1ZGU9RkFMU0UsIGV2YWw9RkFMU0V9CiMgY2hlY2sgaWYgYXNzZXNzZWQgZmlsZSBleGlzdHMKaWYgKCFmaWxlLmV4aXN0cyhmYWlsZWRfcWNfYXNzZXNzZWRfZmlsZSkpIHsKICBzdG9wKHBhc3RlMChmYWlsZWRfcWNfYXNzZXNzZWRfZmlsZSwgJyBub3QgZm91bmQuXG4nLCAKICAgICAgICAgICAgICAnWW91IG5lZWQgdG8gbWFudWFsbHkgYXNzZXNzIHRoZSBmYWlsZWQgc2VnbWVudHMgYWZ0ZXIgc2VnbWVudCBsZXZlbCBRQycsCiAgICAgICAgICAgICAgJyBhbmQgc2F2ZSB0aGUgYXNzZXNzZWQgdGFibGUgdG8gdGhlIHNhbWUgZm9sZGVyIHdpdGggdGhlIHN1ZmZpeCAiX2Fzc2Vzc2VkLmNzdiIuJykpCn0KIyByZWFkIGFzc2Vzc2VkIGZpbGUKZmFpbGVkX3FjX3NlZ21lbnRzX2FsbCA8LSByZWFkX3RzdihmaWxlID0gZmFpbGVkX3FjX2Fzc2Vzc2VkX2ZpbGUpICU+JQogIHNlbGVjdChTYW1wbGVfSUQsIE1hbnVhbEFzc2Vzc21lbnQsIEFzc2Vzc21lbnRDb21tZW50KSAlPiUKICBtdXRhdGUoU2FtcGxlX0lEID0gcGFzdGUwKFNhbXBsZV9JRCwgJy5kY2MnKSkgJT4lCiAgY29sdW1uX3RvX3Jvd25hbWVzKHZhciA9ICdTYW1wbGVfSUQnKQoKIyBqb2luIHRoZSBmYWlsZWQgUUMgc2VnbWVudCBkZiB0byBzZWdtZW50UUNSZXN1bHRzIChmaWx0ZXJpbmcgaXMgYmFzZWQgb24gc2VnbWVudFFDUmVzdWx0cyB0YWJsZSkKc2VnbWVudFFDUmVzdWx0cyA8LSBtZXJnZShzZWdtZW50UUNSZXN1bHRzLCBmYWlsZWRfcWNfc2VnbWVudHNfYWxsLCBieSA9ICdyb3cubmFtZXMnLCBhbGwgPSBUUlVFKQoKc2VnbWVudFFDUmVzdWx0cyA8LSBzZWdtZW50UUNSZXN1bHRzICU+JQogIG11dGF0ZSgKICAgIE1hbnVhbEFzc2Vzc21lbnQgPSBpZl9lbHNlKGlzLm5hKE1hbnVhbEFzc2Vzc21lbnQpLCAnSU5DTFVERScsIE1hbnVhbEFzc2Vzc21lbnQpCiAgKQoKIyBob3cgbWFueSByZXBsaWNhdGVzIHBlciBSZXBsaWNhdGVfSUQgZG8gd2UgbG9zZT8Kc2VnbWVudFFDUmVzdWx0c19yZXBsaWNhdGVzX3Bvc3QgPC0gc2VnbWVudFFDUmVzdWx0c19wcmUgJT4lCiAgaW5uZXJfam9pbihzZWdtZW50UUNSZXN1bHRzLCBieSA9IGMoJ1Jvdy5uYW1lcycsICdMb3dSZWFkcycsICdMb3dUcmltbWVkJywKICAgICAgICAgICAgICAgICAgICAgICAgICAgICAgICdMb3dTdGl0Y2hlZCcsICdMb3dBbGlnbmVkJywgJ0xvd1NhdHVyYXRpb24nLAogICAgICAgICAgICAgICAgICAgICAgICAgICAgICAgJ0xvd05lZ2F0aXZlcycsICdIaWdoTlRDJywgJ0xvd051Y2xlaScsCiAgICAgICAgICAgICAgICAgICAgICAgICAgICAgICAnTG93QXJlYScsICdRQ1N0YXR1cycsICduRmxhZ3MnKSkgJT4lCiAgZ3JvdXBfYnkoUmVwbGljYXRlX0lELCBNYW51YWxBc3Nlc3NtZW50KSAlPiUKICBzdW1tYXJpc2Uobl9yZXBfZmxhZyA9IG4oKSkgJT4lCiAgZ3JvdXBfYnkoUmVwbGljYXRlX0lEKSAlPiUKICBtdXRhdGUobl9yZXAgPSBzdW0obl9yZXBfZmxhZykpICU+JQogIHVuZ3JvdXAoKSAlPiUKICBtdXRhdGUoCiAgICBQcm9wRmxhZ2dlZCA9IHJvdW5kKChuX3JlcF9mbGFnIC8gbl9yZXApLCAyKSwKICAgIFByb3BGbGFnZ2VkTGFiZWwgPSBwYXN0ZTAobl9yZXBfZmxhZywgJy8nLCBuX3JlcCkKICAgICkgJT4lCiAgbXV0YXRlKAogICAgdGVzdF9sYWIgPSBpZl9lbHNlKE1hbnVhbEFzc2Vzc21lbnQgJWluJSBjKCdSRU1PVkUnLCAnQ09OU0lERVInKSwgJ3JtdicsICdpbmMnKQogICkgJT4lCiAgZ3JvdXBfYnkoUmVwbGljYXRlX0lELCB0ZXN0X2xhYikgJT4lCiAgbXV0YXRlKAogICAgdGVzdCA9IHN1bShuX3JlcF9mbGFnKQogICkgJT4lCiAgdW5ncm91cCgpICU+JQogIG11dGF0ZSgKICAgIFJlcGxpY2F0ZVdhcm5pbmcgPSBjYXNlX3doZW4oCiAgICAgIE1hbnVhbEFzc2Vzc21lbnQgJWluJSBjKCdSRU1PVkUnLCAnQ09OU0lERVInKSAmIHRlc3QgPT0gbl9yZXAgfiAnTk8gUkVQTElDQVRFUyBMRUZUJywKICAgICAgTWFudWFsQXNzZXNzbWVudCAlaW4lIGMoJ1JFTU9WRScsICdDT05TSURFUicpICYgbl9yZXAgLSBuX3JlcF9mbGFnID09IDAgfiAnTk8gUkVQTElDQVRFUyBMRUZUJywKICAgICAgTWFudWFsQXNzZXNzbWVudCAlaW4lIGMoJ1JFTU9WRScsICdDT05TSURFUicpICYgbl9yZXAgLSBuX3JlcF9mbGFnID09IDEgfiAnMSBSRVBMSUNBVEVTIExFRlQnLAogICAgICBUUlVFIH4gTkFfY2hhcmFjdGVyXwogICAgKQogICkgJT4lCiAgc2VsZWN0KC1uX3JlcF9mbGFnLCAtbl9yZXAsIC10ZXN0X2xhYiwgLXRlc3QpICU+JQogIGFycmFuZ2UoZGVzYyhNYW51YWxBc3Nlc3NtZW50KSwgZGVzYyhQcm9wRmxhZ2dlZCksIFJlcGxpY2F0ZVdhcm5pbmcpCmBgYAoKU3Vic2V0dGluZyBvdXIgZGF0YXNldDogcmVtb3ZlIHNhbXBsZXMgd2hpY2ggZGlkIG5vdCBwYXNzIFFDLgoKYGBge3Igc3Vic2V0dGluZ19RQ19mYWlsc30KIyBEYXRhIDwtIERhdGFbLCBzZWdtZW50UUNSZXN1bHRzJE1hbnVhbEFzc2Vzc21lbnQgJWluJSBjKCdJTkNMVURFJyldCkRhdGEgPC0gRGF0YVssIHNlZ21lbnRRQ1Jlc3VsdHMkUUNTdGF0dXMgPT0gJ1BBU1MnXQojIGRpbShEYXRhKQpgYGAKCiMjIDQuMiBQcm9iZSBRQwoKR2VuZXJhbGx5IGtlZXAgdGhlIHFjQ3V0b2ZmcyBwYXJhbWV0ZXJzIGZvciBwcm9iZSBRQyB1bmNoYW5nZWQuIFNldAoqcmVtb3ZlTG9jYWxPdXRsaWVycyogdG8gYEZBTFNFYCBpZiB5b3UgZG8gbm90IHdhbnQgdG8gcmVtb3ZlIGxvY2FsCm91dGxpZXJzLiBUbyBjaGVjayB3aGljaCBsb2NhbCBvdXRsaWVycyBoYXZlIGJlZW4gZmxhZ2dlZCwgdXNlIGBleHBycyhEYXRhKWA7IHRoZSBvdXRsaWVycyBhcmUgbWFya2VkIGBOQWAgYW5kIGFyZSByZW1vdmVkIHdoZW4gYWdncmVnYXRpbmcgdGhlIHByb2JlIGNvdW50cyBwZXIgdGFyZ2V0IHdpdGhpbmcgdGhlIGBhZ2dyZWdhdGVDb3VudHMoRGF0YSlgIGZ1bmN0aW9uIGRvd25zdHJlYW0gdmlhIHRoZSBgbmEucm0gPSBUUlVFYCBwYXJhbWV0ZXIuCgpgYGB7ciBwcm9jZXNzX1FDfQpEYXRhIDwtIHNldEJpb1Byb2JlUUNGbGFncyhEYXRhLAogICAgICAgICAgICAgICAgICAgICAgICAgICBxY0N1dG9mZnMgPSBsaXN0KG1pblByb2JlUmF0aW8gPSAwLjEsCiAgICAgICAgICAgICAgICAgICAgICAgICAgICAgICAgICAgICAgICAgICAgcGVyY2VudEZhaWxHcnViYnMgPSAyMCksCiAgICAgICAgICAgICAgICAgICAgICAgICAgIHJlbW92ZUxvY2FsT3V0bGllcnMgPSBUUlVFKQpQcm9iZVFDUmVzdWx0cyA8LSBmRGF0YShEYXRhKVtbJ1FDRmxhZ3MnXV0KCnByb2JlUUNfdG1wIDwtIFByb2JlUUNSZXN1bHRzICU+JQogIHNlbGVjdChHbG9iYWxHcnViYnNPdXRsaWVyKSAlPiUKICByb3duYW1lc190b19jb2x1bW4odmFyID0gJ1JUU19JRCcpCgpwcm9iZVFDX3RtcDIgPC0gcHJvYmVRQ190bXAgJT4lCiAgaW5uZXJfam9pbihmRGF0YShEYXRhKSwgYnkgPSBjKCdSVFNfSUQnKSkKCiMgaG93IG1hbnkgcHJvYmVzIHBlciBnZW5lIHRhcmdldD8KbWVhbl9udW1iZXJfb2ZfcHJvYmVzX3Blcl9nZW5lIDwtIHByb2JlUUNfdG1wMiAlPiUKICBncm91cF9ieShUYXJnZXROYW1lKSAlPiUKICBkcGx5cjo6Y291bnQoKSAlPiUKICB1bmdyb3VwKCkgJT4lCiAgc3VtbWFyaXNlKG1lYW4gPSBtZWFuKG4pKSAlPiUKICBwdWxsKCkKClByb2JlUUNSZXN1bHRzJFFDU3RhdHVzIDwtIGFwcGx5KFByb2JlUUNSZXN1bHRzLCAxTCwgZnVuY3Rpb24oeCkgewogIGlmZWxzZShzdW0oeCkgPT0gMEwsICdQQVNTJywgJ1dBUk5JTkcnKQp9KQoKTG9jYWxfb3V0bGllcnMgPC0gUHJvYmVRQ1Jlc3VsdHMgJT4lCiAgc2VsZWN0KHN0YXJ0c193aXRoKCdMb2NhbEdydWJic091dGxpZXInKSkKbkxvY2FsX291dGxpZXJzIDwtIGNvbFN1bXMoTG9jYWxfb3V0bGllcnMpCm5Mb2NhbF9vdXRsaWVycyA8LSBzb3J0KG5Mb2NhbF9vdXRsaWVycywgZGVjcmVhc2luZyA9IFQpCkxvY2FsX291dGxpZXJzJG5TZWdtZW50T3V0bGllciA8LSBhcHBseShMb2NhbF9vdXRsaWVycywgMUwsIGZ1bmN0aW9uKHgpIHsKICBzdW0oeCkKfSkKCiMgc29ydApMb2NhbF9vdXRsaWVycyA8LSBMb2NhbF9vdXRsaWVycyAlPiUKICBhcnJhbmdlKGRlc2MoblNlZ21lbnRPdXRsaWVyKSkKCnRvdGFsX2xvY2FsX291dGxpZXJzIDwtIHN1bShMb2NhbF9vdXRsaWVycyRuU2VnbWVudE91dGxpZXIpCgpuUHJvYmVzX3dpdGhfZG91YmxlX2RpZ2l0X3NlZ21lbnRfb3V0bGllcnMgPC0gTG9jYWxfb3V0bGllcnMgJT4lCiAgZmlsdGVyKG5TZWdtZW50T3V0bGllciA+IDkpICU+JQogIHN1bW1hcmlzZShzdW0gPSBuKCkpICU+JSBwdWxsKCkKCiMjIyBPUFRJT05BTDogd3JpdGUgUHJvYmUgUUMgbmFub3N0cmluZyBvYmplY3QgdG8gZGlzayB0byBjb21wYXJlIENUQSAmIFdUQSBwcm9iZXMKaWYgKHNhdmVfb2JqZWN0KSB7CiAgc2F2ZVJEUygKICAgIG9iamVjdCA9IERhdGEsCiAgICBmaWxlID0gcGFzdGUwKG91dHB1dF9kaXIsICcvJywgaW5wdXRfZXhwZXJpbWVudCwgJy9vdXRwdXQvcWNfcmVzdWx0cy8nLCBxY19hc3Nlc3NtZW50X2RhdGUsICcvcHJvYmVxY19EYXRhLlJEUycpCiAgKQp9CmBgYAoKVGhlcmUgYXJlIGByIG5yb3coUHJvYmVRQ1Jlc3VsdHMpYCBwcm9iZXMgdGVzdGVkIHdpdGggYW4gYXZlcmFnZSBvZiBvZgpgciByb3VuZChtZWFuX251bWJlcl9vZl9wcm9iZXNfcGVyX2dlbmUsIDIpYCBwcm9iZXMgcGVyIGdlbmUgdGFyZ2V0IGluCmBmZWF0dXJlRGF0YWAuIEEgdG90YWwgb2YgYHIgdG90YWxfbG9jYWxfb3V0bGllcnNgIGxvY2FsIG91dGxpZXJzIG91dCBvZgpgciBwcm9kKGRpbShMb2NhbF9vdXRsaWVyc1ssIC0xXSkpYAooYHIgcm91bmQodG90YWxfbG9jYWxfb3V0bGllcnMgLyBwcm9kKGRpbShMb2NhbF9vdXRsaWVyc1ssIC0xXSkpICogMTAwLCAyKWAlKQpwb3NzaWJsZSBvdXRsaWVycyB3ZXJlIGlkZW50aWZpZWQgYWNjb3JkaW5nIHRvIHRoZSBHcnViYidzIHRlc3QuIFRoZQpzZWdtZW50IHdpdGggdGhlIGhpZ2hlc3QgbnVtYmVyIG9mIGxvY2FsIG91dGxpZXJzCihgciBuTG9jYWxfb3V0bGllcnNbWzFdXWApIHdhcwpgciBzdHJfcmVwbGFjZV9hbGwobmFtZXMobkxvY2FsX291dGxpZXJzWzFdKSwgcGF0dGVybiA9IHJlZ2V4KCdeLisoRFNQLispXFwuZGNjJyksIHJlcGxhY2VtZW50ID0gJ1xcMScpYC4KYHIgblByb2Jlc193aXRoX2RvdWJsZV9kaWdpdF9zZWdtZW50X291dGxpZXJzYCBwcm9iZXMgd2VyZSBmb3VuZCB0byBiZQpsb2NhbCBvdXRsaWVycyBpbiBhdCBsZWFzdCAxMCBzZWdtZW50cy4gVGhlIG1vc3QgZmF1bHR5IHByb2JlIHdhcwpgciByb3duYW1lcyhMb2NhbF9vdXRsaWVyc1sxLF0pYCB3aGljaCB3YXMgZm91bmQgdG8gYmUgYW4gb3V0bGllciBpbgpgciByb3VuZChMb2NhbF9vdXRsaWVyc1sxLGxlbmd0aChMb2NhbF9vdXRsaWVycyldIC8gbmNvbChMb2NhbF9vdXRsaWVycykgKiAxMDAsIDApYCUKb2Ygc2VnbWVudHMuIE1lYW4gYW5kIG1lZGlhbiBudW1iZXIgb2Ygb3V0bGllciBzZWdtZW50cyBwZXIgcHJvYmUgd2VyZQpgciByb3VuZChtZWFuKExvY2FsX291dGxpZXJzWywgbmNvbChMb2NhbF9vdXRsaWVycyldKSwgMSlgIGFuZApgciBtZWRpYW4oTG9jYWxfb3V0bGllcnNbLCBuY29sKExvY2FsX291dGxpZXJzKV0pYCwgcmVzcGVjdGl2ZWx5Ljxicj4KRURJVDogQWZ0ZXIgYXNzZXNzaW5nIGhvdyBtYW55IHByb2JlcyB3ZXJlIGZsYWdnZWQgYXMgbG9jYWwgb3V0bGllcnMsIHdlCmRlY2lkZWQgdG8gZXhjbHVkZSB0aGVtLCBiZWNhdXNlIHdlIGJlbGlldmUgdGhhdCB3ZSBkb24ndCBsb3NlIHRvbyBtdWNoCmluZm9ybWF0aW9uLjxicj48YnI+CgpEZWZpbmUgUUMgdGFibGUgZm9yIFByb2JlIFFDCgpgYGB7ciBkZWZpbmVfcWNfdGFibGV9CnJtKExvY2FsX291dGxpZXJzKQpwcm9iZVFDX2RmIDwtIGRhdGEuZnJhbWUoUGFzc2VkID0gc3VtKHJvd1N1bXMoUHJvYmVRQ1Jlc3VsdHNbLCBncmVwbCgnR3J1YmJzT3V0bGllcicsIGNvbG5hbWVzKFByb2JlUUNSZXN1bHRzKSldKSA9PSAwKSwKICAgICAgICAgICAgICAgICAgICBHbG9iYWwgPSBzdW0oUHJvYmVRQ1Jlc3VsdHMkR2xvYmFsR3J1YmJzT3V0bGllciksCiAgICAgICAgICAgICAgICAgICAgTG9jYWwgPSBzdW0ocm93U3VtcyhQcm9iZVFDUmVzdWx0c1ssIGdyZXBsKCdMb2NhbEdydWJic091dGxpZXInLCBjb2xuYW1lcyhQcm9iZVFDUmVzdWx0cykpXSkgPiAwCiAgICAgICAgICAgICAgICAgICAgICAgICAgICAgICAgJiAhUHJvYmVRQ1Jlc3VsdHMkR2xvYmFsR3J1YmJzT3V0bGllcikpCmBgYAoKU3Vic2V0IG9iamVjdCB0byBleGNsdWRlIGFsbCB0aGF0IGRpZCBub3QgcGFzcyBwcm9iZSByYXRpbyAmIGdsb2JhbCB0ZXN0aW5nLgoKYGBge3Igc3Vic2V0fQpQcm9iZVFDUGFzc2VkIDwtIHN1YnNldChEYXRhLAogICAgICAgICAgICAgICAgICAgICAgICBmRGF0YShEYXRhKVtbJ1FDRmxhZ3MnXV1bLCBjKCdMb3dQcm9iZVJhdGlvJyldID09IEZBTFNFICYKICAgICAgICAgICAgICAgICAgICAgICAgICBmRGF0YShEYXRhKVtbJ1FDRmxhZ3MnXV1bLCBjKCdHbG9iYWxHcnViYnNPdXRsaWVyJyldID09IEZBTFNFKQpkaW0oUHJvYmVRQ1Bhc3NlZCkKCkRhdGEgPC0gUHJvYmVRQ1Bhc3NlZApgYGAKCkNoZWNrIGhvdyBtYW55IHVuaXF1ZSB0YXJnZXRzIChpLmUuIGdlbmVzKSB0aGUgb2JqZWN0IGhhcy4KCmBgYHtyIHVuaXF1ZV9jaGVja30KbGVuZ3RoKHVuaXF1ZShmZWF0dXJlRGF0YShEYXRhKVtbJ1RhcmdldE5hbWUnXV0pKQpgYGAKCiMjIyBDb2xsYXBzZSB0byB0YXJnZXRzCgpUaGUgcHJvYmVzIGFyZSBhZ2dyZWdhdGVkIHBlciB0YXJnZXQgYnkgdXNpbmcgdGhlIGdlb21ldHJpYyBtZWFuIG9mIGFsbCB0YXJnZXQgcHJvYmVzIGluIGFsbCBzZWdtZW50cyBieSBkZWZhdWx0Ljxicj4gVGhlIGZ1bmN0aW9uIGFsc28gY2FsY3VsYXRlcyB0aGUgTmVnR2VvTWVhbiBhbmQgTmVnR2VvU0QgZm9yIGVhY2ggc2VnbWVudCAmIG1vZHVsZSBhbmQgYXBwZW5kcyB0aGlzIGluZm9ybWF0aW9uIHRvIGBwRGF0YShEYXRhKWAuCgpgYGB7ciBjb2xsYXBzX3RhcmdldHN9CnRhcmdldF9EYXRhIDwtIGFnZ3JlZ2F0ZUNvdW50cyhEYXRhKQpkaW0odGFyZ2V0X0RhdGEpCgpleHBycyh0YXJnZXRfRGF0YSlbMTo1LCAxOjJdCmBgYAoKIyMgNC4zIEJhY2tncm91bmQgcXVhbnRpZmljYXRpb24gUUMgCgojIyMgNC4zLjEgTGltaXQgb2YgUXVhbnRpZmljYXRpb24KCldlIGRlZmluZSBhIGxpbWl0IG9mIHF1YW50aWZpY2F0aW9uIChMT1EpIHBlciBST0kvQU9JIHNlZ21lbnQgYmFzZWQgb24KdGhlIG5lZ2F0aXZlIGNvbnRyb2wgcHJvYmVzIHRvIGd1aWRlIHRoZSBmaWx0ZXJpbmcgb2Ygc2VnbWVudHMgYW5kIGdlbmVzCndpdGggbG93IHNpZ25hbCByZWxhdGl2ZSB0byBiYWNrZ3JvdW5kLiBUaGUgZm9ybXVsYSBmb3IgY2FsY3VsYXRpbmcgdGhlCkxPUSBpbiB0aGUgJGlee3RofSQgc2VnbWVudCBhdCAkbiQgc3RhbmRhcmQgZGV2aWF0aW9ucyA8YnI+CigkbiA9IDIkIGZvciB0aGlzIHN0dWR5KSBpczogJExPUV9pPWdlb21lYW4oTmVnUHJvYmVfaSkqZ2VvU0QoTmVnUHJvYmVfaSlebiQ8YnI+PGJyPgoKRGVmaW5lIExPUSBTRCB0aHJlc2hvbGQgYW5kIG1pbmltdW0gdmFsdWUKCmBgYHtyIHNldF9MT1F9CkxPUVNEX2N1dG9mZiA8LSAwLjUKbWluTE9RIDwtIDIKYGBgCgpDYWxjdWxhdGUgTE9RIHBlciBtb2R1bGUgdGVzdGVkCgpgYGB7ciBjYWxjdWxhdGVfTE9RfQpMT1EgPC0gZGF0YS5mcmFtZShyb3cubmFtZXMgPSBjb2xuYW1lcyh0YXJnZXRfRGF0YSkpCgpmb3IobW9kdWxlIGluIG1vZHVsZXMpIHsKICB2YXJzIDwtIHBhc3RlMChjKCdOZWdHZW9NZWFuXycsICdOZWdHZW9TRF8nKSwKICAgICAgICAgICAgICAgICBtb2R1bGUpCiAgaWYoYWxsKHZhcnNbMToyXSAlaW4lIGNvbG5hbWVzKHBEYXRhKHRhcmdldF9EYXRhKSkpKSB7CiAgICBMT1FbLCBtb2R1bGVdIDwtIHBtYXgobWluTE9RLAogICAgICAgICAgICAgICAgICAgICAgICAgIHBEYXRhKHRhcmdldF9EYXRhKVssIHZhcnNbMV1dICogcERhdGEodGFyZ2V0X0RhdGEpWywgdmFyc1syXV0gXiBMT1FTRF9jdXRvZmYKICAgICAgICAgICAgICAgICAgICAgICAgICApCiAgfQp9CgpwRGF0YSh0YXJnZXRfRGF0YSkkTE9RIDwtIExPUQpgYGAKCiMjIyA0LjMuMiBMb3cgbmVnYXRpdmUgYmFja2dyb3VuZCBmaWx0ZXJpbmcKCkFmdGVyIGRldGVybWluaW5nIHRoZSBsaW1pdCBvZiBxdWFudGlmaWNhdGlvbiAoTE9RKSBwZXIgc2VnbWVudCwgd2UKcmVjb21tZW5kIGZpbHRlcmluZyBvdXQgZWl0aGVyIHNlZ21lbnRzIGFuZC9vciBnZW5lcyB3aXRoIGFibm9ybWFsbHkgbG93CnNpZ25hbC4gRmlsdGVyaW5nIGlzIGFuIGltcG9ydGFudCBzdGVwIHRvIGZvY3VzIG9uIHRoZSB0cnVlIGJpb2xvZ2ljYWwKZGF0YSBvZiBpbnRlcmVzdC4gV2UgZGV0ZXJtaW5lIHRoZSBudW1iZXIgb2YgZ2VuZXMgZGV0ZWN0ZWQgaW4gZWFjaApzZWdtZW50IGFjcm9zcyB0aGUgZGF0YXNldC4KCmBgYHtyIGZpbHRlcmluZ30KTE9RX01hdCA8LSBjKCkKZm9yKG1vZHVsZSBpbiBtb2R1bGVzKSB7CiAgaW5kIDwtIGZEYXRhKHRhcmdldF9EYXRhKSRNb2R1bGUgPT0gbW9kdWxlCiAgTWF0X2kgPC0gdChlc0FwcGx5KHRhcmdldF9EYXRhW2luZCwgXSwgTUFSR0lOID0gMSwKICAgICAgICAgICAgICAgICAgICAgRlVOID0gZnVuY3Rpb24oeCkgewogICAgICAgICAgICAgICAgICAgICAgIHggPiBMT1FbLCBtb2R1bGVdCiAgICAgICAgICAgICAgICAgICAgIH0pKQogIExPUV9NYXQgPC0gcmJpbmQoTE9RX01hdCwgTWF0X2kpCn0KCiMgZW5zdXJlIG9yZGVyaW5nIHNpbmNlIHRoaXMgaXMgc3RvcmVkIG91dHNpZGUgb2YgdGhlIGdlb214U2V0CkxPUV9NYXQgPC0gTE9RX01hdFtmRGF0YSh0YXJnZXRfRGF0YSkkVGFyZ2V0TmFtZSwgXQpgYGAKCiMjIyA0LjMuMyBTZWdtZW50IEdlbmUgRGV0ZWN0aW9uIHsudGFic2V0IC50YWJzZXQtcGlsbHN9CgpXZSBmaXJzdCBmaWx0ZXIgb3V0IHNlZ21lbnRzIHdpdGggZXhjZXB0aW9uYWxseSBsb3cgc2lnbmFsLiBUaGVzZQpzZWdtZW50cyB3aWxsIGhhdmUgYSBzbWFsbCBmcmFjdGlvbiBvZiBwYW5lbCBnZW5lcyBkZXRlY3RlZCBhYm92ZSB0aGUKTE9RIHJlbGF0aXZlIHRvIHRoZSBvdGhlciBzZWdtZW50cyBpbiB0aGUgc3R1ZHkuIExldCdzIHZpc3VhbGl6ZSB0aGUKZGlzdHJpYnV0aW9uIG9mIHNlZ21lbnRzIHdpdGggcmVzcGVjdCB0byB0aGVpciAlIGdlbmVzIGRldGVjdGVkOgoKU2F2ZSBkZXRlY3Rpb24gcmF0ZSBpbmZvcm1hdGlvbiB0byBwaGVubyBkYXRhCgpgYGB7ciBzYXZlX2RldGVjdGlvbl9yYXRlfQpwRGF0YSh0YXJnZXRfRGF0YSkkR2VuZXNEZXRlY3RlZCA8LSBjb2xTdW1zKExPUV9NYXQsIG5hLnJtID0gVFJVRSkKcERhdGEodGFyZ2V0X0RhdGEpJEdlbmVEZXRlY3Rpb25SYXRlIDwtIHBEYXRhKHRhcmdldF9EYXRhKSRHZW5lc0RldGVjdGVkIC8gbnJvdyh0YXJnZXRfRGF0YSkKYGBgCgpEZXRlcm1pbmUgZGV0ZWN0aW9uIHRocmVzaG9sZHM6IDElLCA1JSwgMTAlLCAxNSUsIFw+MTUlCgpgYGB7ciBkZXRlcm1pbmUrdGhyZXNob2xkc30KcERhdGEodGFyZ2V0X0RhdGEpJERldGVjdGlvblRocmVzaG9sZCA8LSBjdXQocERhdGEodGFyZ2V0X0RhdGEpJEdlbmVEZXRlY3Rpb25SYXRlLAogICAgICAgICAgICAgICAgICAgICAgICAgICAgICAgICAgICAgICAgICAgICBicmVha3MgPSBjKDAsIDAuMDEsIDAuMDUsIDAuMSwgMC4xNSwgMC4yLCAxKSwKICAgICAgICAgICAgICAgICAgICAgICAgICAgICAgICAgICAgICAgICAgICAgbGFiZWxzID0gYygnPDElJywgJzEtNSUnLCAnNS0xMCUnLCAnMTAtMTUlJywgJzE1LTIwJScsICc+MjAlJykpCmBgYAoKYGBge3J9CiMgY29sdW1uIHRvIGZpbGwgcGxvdHMgYnkKY29sX2J5IDwtICdzZWdtZW50JwoKIyBnZW5lIGRldGVjdGlvbiByYXRlIHBsb3R0aW5nIGZ1bmN0aW9uCmdlbmVfZGV0X3JhdGVfYmFycGxvdCA8LSBmdW5jdGlvbihhc3NheV9kYXRhID0gTlVMTCwKICAgICAgICAgICAgICAgICAgICAgICAgIGFubm90YXRpb24gPSBOVUxMLAogICAgICAgICAgICAgICAgICAgICAgICAgZmlsbF9ieSA9IE5VTEwpIHsKICBwbHQgPC0gZ2dwbG90KGFzc2F5X2RhdGEsCiAgICAgICAgICAgICAgICBhZXMoeCA9IERldGVjdGlvblRocmVzaG9sZCkpICsKICBnZW9tX2JhcihhZXNfc3RyaW5nKGZpbGwgPSBwYXN0ZTAoJ3VubGlzdChgJywgZmlsbF9ieSwgJ2ApJykpKSArCiAgZ2VvbV90ZXh0KHN0YXQgPSAnY291bnQnLCBhZXMobGFiZWwgPSAuLmNvdW50Li4pLCB2anVzdCA9IC0wLjUpICsKICB0aGVtZV9idygpICsKICBzY2FsZV95X2NvbnRpbnVvdXMoZXhwYW5kID0gZXhwYW5zaW9uKG11bHQgPSBjKDAsIDAuMSkpKSArCiAgc2NhbGVfZmlsbF9tYW51YWwodmFsdWVzID0gYW5uX2NvbG9yc1tbZmlsbF9ieV1dKSArCiAgbGFicyh4ID0gJ0dlbmUgRGV0ZWN0aW9uIFJhdGUnLAogICAgICAgeSA9ICdzZWdtZW50cywgIycsCiAgICAgICBmaWxsID0gYW5ub3RhdGlvbikKICAKICByZXR1cm4ocGx0KQp9CmBgYAoKCiMjIyMgQnkgU2VnbWVudCBUeXBlCgpgYGB7cn0KIyBzdGFja2VkIGJhciBwbG90IG9mIGRpZmZlcmVudCBjdXQgcG9pbnRzICgxJSwgNSUsIDEwJSwgMTUlKQpnZW5lX2RldF9yYXRlX2JhcnBsb3QocERhdGEodGFyZ2V0X0RhdGEpLCBhbm5vdGF0aW9uID0gJ1NlZ21lbnQnLCBmaWxsX2J5ID0gJ3NlZ21lbnQnKQoKa2FibGUodGFibGUocERhdGEodGFyZ2V0X0RhdGEpJERldGVjdGlvblRocmVzaG9sZCwKICAgICAgICAgICAgcERhdGEodGFyZ2V0X0RhdGEpJHNlZ21lbnQpKQpgYGAKCiMjIyMgQnkgUmVnaW9uCgpgYGB7cn0KIyBzdGFja2VkIGJhciBwbG90IG9mIGRpZmZlcmVudCBjdXQgcG9pbnRzICgxJSwgNSUsIDEwJSwgMTUlKQpnZW5lX2RldF9yYXRlX2JhcnBsb3QocERhdGEodGFyZ2V0X0RhdGEpLCBhbm5vdGF0aW9uID0gJ1Rpc3N1ZSByZWdpb24nLCBmaWxsX2J5ID0gJ3Rpc3N1ZVJlZ2lvbicpCgprYWJsZSh0YWJsZShwRGF0YSh0YXJnZXRfRGF0YSkkRGV0ZWN0aW9uVGhyZXNob2xkLAogICAgICAgICAgICBwRGF0YSh0YXJnZXRfRGF0YSkkdGlzc3VlUmVnaW9uKSkKYGBgCgojIyMjIEJ5IFBhdGllbnQKCmBgYHtyfQojIHN0YWNrZWQgYmFyIHBsb3Qgb2YgZGlmZmVyZW50IGN1dCBwb2ludHMgKDElLCA1JSwgMTAlLCAxNSUpCmdlbmVfZGV0X3JhdGVfYmFycGxvdChwRGF0YSh0YXJnZXRfRGF0YSksIGFubm90YXRpb24gPSAnUGF0aWVudCBJRCcsIGZpbGxfYnkgPSAnUGF0aWVudF9JRCcpCgprYWJsZSh0YWJsZShwRGF0YSh0YXJnZXRfRGF0YSkkRGV0ZWN0aW9uVGhyZXNob2xkLAogICAgICAgICAgICBwRGF0YSh0YXJnZXRfRGF0YSkkUGF0aWVudF9JRCkpCmBgYAoKIyMjIyBCeSBTbGlkZQoKYGBge3J9CiMgc3RhY2tlZCBiYXIgcGxvdCBvZiBkaWZmZXJlbnQgY3V0IHBvaW50cyAoMSUsIDUlLCAxMCUsIDE1JSkKZ2VuZV9kZXRfcmF0ZV9iYXJwbG90KHBEYXRhKHRhcmdldF9EYXRhKSwgYW5ub3RhdGlvbiA9ICdTbGlkZScsIGZpbGxfYnkgPSAnc2xpZGUgbmFtZScpCgprYWJsZSh0YWJsZShwRGF0YSh0YXJnZXRfRGF0YSkkRGV0ZWN0aW9uVGhyZXNob2xkLAogICAgICAgICAgICBwRGF0YSh0YXJnZXRfRGF0YSkkYHNsaWRlIG5hbWVgKSkKYGBgCgojIyMgR2VuZSBkZXRlY3Rpb24gcmF0ZSB2aXN1YWxpemVkCgpgYGB7ciwgZmlnLndpZHRoPTEwLCBmaWcuaGVpZ2h0PTUsIHdhcm5pbmc9RkFMU0V9CiMgc2V0IHRocmVzaG9sZCBmb3IgZGV0ZWN0aW9ubGV2ZWwKIyBkZWZhdWx0IDAuMQpkZWZhdWx0X2dlbmVfZGV0X3RocmVzaG9sZCA8LSAwLjEKZ2VuZV9kZXRfdGhyZXNob2xkIDwtIDAuMDUKCiMgY2FsYyBtZWRpYW4gZ2VuZSBkZXRlY3Rpb24gcmF0ZQptZWRpYW5fZ2VuZV9kZXRlY3Rpb25fcmF0ZSA8LSBtZWRpYW4ocERhdGEodGFyZ2V0X0RhdGEpJEdlbmVEZXRlY3Rpb25SYXRlKQoKIyB2aXN1YWxpemUgZ2VuZSBkZXRlY3Rpb24gcmF0ZSBhcyBkZW5zaXR5IGhpc3RvZ3JhbSAKZ2dwbG90KHBEYXRhKHRhcmdldF9EYXRhKSwKICAgICAgIGFlcygKICAgICAgICAgeCA9IEdlbmVEZXRlY3Rpb25SYXRlCiAgICAgICApKSArCiAgZ2VvbV9oaXN0b2dyYW0oCiAgICBhZXMoCiAgICAgIHkgPSAuLmRlbnNpdHkuLgogICAgICApLAogICAgYmlucyA9IDUwLAogICAgY29sb3IgPSAnYmxhY2snLAogICAgZmlsbCA9ICd3aGl0ZScKICAgICkgKwogIGdlb21fZGVuc2l0eSgKICAgIGNvbG9yID0gJ3N0ZWVsYmx1ZScsCiAgICBmaWxsID0gJ3N0ZWVsYmx1ZScsCiAgICBhbHBoYSA9IDAuMwogICAgKSArCiAgZ2VvbV92bGluZSgKICAgIGFlcygKICAgICAgeGludGVyY2VwdCA9IG1lZGlhbl9nZW5lX2RldGVjdGlvbl9yYXRlCiAgICApLAogICAgY29sb3IgPSAnIzZDN0I4QicsCiAgICBsd2QgPSAyCiAgKSArCiAgZ2VvbV92bGluZSgKICAgIGFlcygKICAgICAgeGludGVyY2VwdCA9IGRlZmF1bHRfZ2VuZV9kZXRfdGhyZXNob2xkCiAgICApLAogICAgY29sb3IgPSAnIzFDODZFRScsCiAgICBsd2QgPSAyCiAgKSArCiAgZ2VvbV92bGluZSgKICAgIGFlcygKICAgICAgeGludGVyY2VwdCA9IGdlbmVfZGV0X3RocmVzaG9sZAogICAgKSwKICAgIGNvbG9yID0gJyMxMDRFOEInLAogICAgbHdkID0gMgogICkgKwogIHNjYWxlX3hfY29udGludW91cygKICAgIGxpbWl0cyA9IGMoLTAuMSwxKSwKICAgIGJyZWFrcyA9IHJvdW5kKGMoc2VxKDAsIDEsIGJ5ID0gMC4yNSksIG1lZGlhbl9nZW5lX2RldGVjdGlvbl9yYXRlKSwgMikKICApCmBgYAoKR3JleSB0aGljayB2ZXJ0aWNhbCBsaW5lIGRlbm90ZXMgdGhlIG1lZGlhbiBnZW5lIGRldGVjdGlvbiByYXRlIGFjcm9zcwphbGwgc2VnbWVudHMvUk9Jcy48YnI+IExpZ2h0Ymx1ZSB0aGljayB2ZXJ0aWNhbCBsaW5lIGRlbm90ZXMgdGhlIGRlZmF1bHQKZ2VuZSBkZXRlY3Rpb24gdGhyZXNob2xkLiBEYXJrYmx1ZSB0aGljayB2ZXJ0aWNhbCBsaW5lIGRlbm90ZXMgdGhlCmNob3NlbiBnZW5lIGRldGVjdGlvbiB0aHJlc2hvbGQgYWZ0ZXIgZXhhbWluaW5nIHRoZSBkYXRhLgoKIyMjIEZpbHRlciBvdXQgc2VnbWVudHMgd2l0aCBhYm5vcm1hbGx5IGxvdyBzaWduYWwgey51bm51bWJlcmVkfQoKYGBge3IgY3V0X3RvX3BlcmNlbnR9CiMgZmlsdGVyIG91dCBzZWdtZW50cyB3aXRoIGFibm9ybWFsbHkgbG93IHNpZ25hbAp0YXJnZXRfRGF0YSA8LSB0YXJnZXRfRGF0YVssIHBEYXRhKHRhcmdldF9EYXRhKSRHZW5lRGV0ZWN0aW9uUmF0ZSA+PSBnZW5lX2RldF90aHJlc2hvbGRdCgpkaW0odGFyZ2V0X0RhdGEpCgojIGNhbGN1bGF0ZSBob3cgbWFueSBzZWdtZW50cyBhcmUgbG9zdCBkdWUgdG8gZ2VuZSBkZXRlY3Rpb24gdGhyZXNob2xkCm5TYW1wbGVzX3ByZWZpbHRlcl9MT1EgPC0gdW5uYW1lKG5jb2woRGF0YSkpCm5TYW1wbGVfcG9zdGZpbHRlcl9MT1EgPC0gdW5uYW1lKG5jb2wodGFyZ2V0X0RhdGEpKQpuU2FtcGxlc19sb3N0X0xPUSA8LSBuU2FtcGxlc19wcmVmaWx0ZXJfTE9RIC0gblNhbXBsZV9wb3N0ZmlsdGVyX0xPUQpgYGAKCkxPUSBmaWx0ZXJpbmcgdGhyZXNob2xkcyB3ZXJlOjxicj4KMS4gbWluaW11bSBMT1Egb2YgYHIgbWluTE9RYCAoZGVmYXVsdDogMikuPGJyPgoyLiBtaW5pbXVtIE5lZ0dlb1NEIF4gYHIgTE9RU0RfY3V0b2ZmYCAoZGVmYXVsdCAkTmVnR2VvU0ReMiQpIHN0YW5kYXJkIGRldmlhdGlvbnMgcGVyIHNlZ21lbnQuPGJyPgozLiBtaW5pbXVtIGdlbmUgZGV0ZWN0aW9uIHJhdGUgb2YgYHIgZ2VuZV9kZXRfdGhyZXNob2xkYCAoZGVmYXVsdDogMC4xKS48YnI+PGJyPgpBZnRlciBmaWx0ZXJpbmcgdGhlIHNlZ21lbnRzIGJ5IGdlbmUgZGV0ZWN0aW9uIHJhdGUgYmFzZWQgb24gTE9RLCB0aGVyZSB3ZXJlIGByIG5TYW1wbGVzX2xvc3RfTE9RYCBzYW1wbGVzIGxvc3QgKHByZWZpbHRlcjogYHIgblNhbXBsZXNfcHJlZmlsdGVyX0xPUWAsIHBvc3RmaWx0ZXI6IGByIG5TYW1wbGVfcG9zdGZpbHRlcl9MT1FgKS4gCgojIyA0LjQgY29sbGVjdCBhbm5vdGF0aW9ucwoKYGBge3Igc2VsZWN0X2Fubm90YXRpb25zMn0KIyAqKlNlbGVjdCB0aGUgYW5ub3RhdGlvbnMgd2Ugd2FudCB0byBzaG93LCB1c2UgYGAgdG8gc3Vycm91bmQgY29sdW1uIG5hbWVzIHdpdGggc3BhY2VzIG9yIHNwZWNpYWwgc3ltYm9scyoqCmNvdW50X21hdCA8LSBkcGx5cjo6Y291bnQocERhdGEoRGF0YSksIHNlZ21lbnQsIHNsaWRlTG9jYXRpb24gLCB0aXNzdWVSZWdpb24sIFBhdGllbnRfSUQsIGBzbGlkZSBuYW1lYCkKCiMgZ2F0aGVyIHRoZSBkYXRhIGFuZCBwbG90IGluIG9yZGVyOiBjbGFzcywgc2xpZGUgbmFtZSwgcmVnaW9uLCBzZWdtZW50CnRlc3RfZ3IgPC0gZ2F0aGVyX3NldF9kYXRhKGNvdW50X21hdCwgMToyKQp0ZXN0X2dyJHggPC0gZmFjdG9yKHRlc3RfZ3IkeCwKICAgICAgICAgICAgICAgICAgICBsZXZlbHMgPSBjKCdzbGlkZSBuYW1lJywgJ3NlZ21lbnQnKSkKCmFvaWxpc3QgPC1uYW1lcyhhcy5kYXRhLmZyYW1lKGFzc2F5RGF0YUVsZW1lbnQodGFyZ2V0X0RhdGEsIGVsdCA9ICdleHBycycpKSkKCnNlZ21lbnQgPC1hcy5kYXRhLmZyYW1lKHBEYXRhKHRhcmdldF9EYXRhKSRzZWdtZW50LCB1bmlxdWUoY291bnRfbWF0JHNlZ21lbnQpKQpjb2xuYW1lcyhzZWdtZW50KSA8LSAnc2VnbWVudCcKcm93Lm5hbWVzKHNlZ21lbnQpIDwtIGFvaWxpc3QKCnNsaWRlTG9jYXRpb24gPC0gYXMuZGF0YS5mcmFtZShwRGF0YSh0YXJnZXRfRGF0YSkkc2xpZGVMb2NhdGlvbiwgdW5pcXVlKGNvdW50X21hdCRzbGlkZUxvY2F0aW9uKSkKY29sbmFtZXMoc2xpZGVMb2NhdGlvbikgPC0gJ0xvY2F0aW9uJwpyb3cubmFtZXMoc2xpZGVMb2NhdGlvbikgPC0gYW9pbGlzdAoKdGlzc3VlUmVnaW9uIDwtIGFzLmRhdGEuZnJhbWUocERhdGEodGFyZ2V0X0RhdGEpJHRpc3N1ZVJlZ2lvbiwgdW5pcXVlKGNvdW50X21hdCR0aXNzdWVSZWdpb24pKQpjb2xuYW1lcyh0aXNzdWVSZWdpb24pIDwtICdTdHJ1Y3R1cmUnCnJvdy5uYW1lcyh0aXNzdWVSZWdpb24pIDwtIGFvaWxpc3QKClBhdGllbnRfSUQgPC0gYXMuZGF0YS5mcmFtZShwRGF0YSh0YXJnZXRfRGF0YSkkUGF0aWVudF9JRCwgdW5pcXVlKGNvdW50X21hdCRQYXRpZW50X0lEKSkKY29sbmFtZXMoUGF0aWVudF9JRCkgPC0gJ2lkJwpyb3cubmFtZXMoUGF0aWVudF9JRCkgPC0gYW9pbGlzdAoKU04gPC0gYXMuZGF0YS5mcmFtZShwRGF0YSh0YXJnZXRfRGF0YSkkYHNsaWRlIG5hbWVgLCB1bmlxdWUoY291bnRfbWF0JGBzbGlkZSBuYW1lYCkpCmNvbG5hbWVzKFNOKSA8LSAnc2xpZGUgbmFtZScKcm93Lm5hbWVzKFNOKSA8LSBhb2lsaXN0Cgphbm4gPC0gY2JpbmQoc2VnbWVudCwgc2xpZGVMb2NhdGlvbiwgdGlzc3VlUmVnaW9uLCBQYXRpZW50X0lELCBTTikKYGBgCgojIyA0LjUgTWFudWFsIHJlbW92YWwgb2Ygc2FtcGxlcy9jbGFzc2VzCgpgYGB7ciByZW1vdmVfc2FtcGxlc30KIyBhY3RpdmVfYW9pcyA8LSByb3duYW1lcyhhbm4pW2FubiRzdGF0dXMhID0gJ0luZmxhbWVkJ10KCiMgdGFyZ2V0X0RhdGEgPC0gIHRhcmdldF9EYXRhWywgYWN0aXZlX2FvaXNdCgojIGRpbSh0YXJnZXRfRGF0YSkKCiMgY291bnRfbWF0IDwtIGNvdW50X21hdFtjb3VudF9tYXQkc2VnbWVudCAhPSAnSW5mbGFtZWQnLF0KYGBgCgpyZS1Db2xsZWN0IGFubm90YXRpb25zCgpgYGB7ciBjb2xsZWN0X2Fubm90YXRpb25zfQojIGdhdGhlciB0aGUgZGF0YSBhbmQgcGxvdCBpbiBvcmRlcjogc2xpZGUgbmFtZSwgc2VnbWVudCwgc2xpZGVMb2NhdGlvbiB0aXNzdWVSZWdpb24sIFBhdGllbnRfSUQKY291bnRfbWF0X2ZpbHRlcmVkIDwtIGRwbHlyOjpjb3VudChwRGF0YSh0YXJnZXRfRGF0YSksIGBzbGlkZSBuYW1lYCwgc2VnbWVudCwgc2xpZGVMb2NhdGlvbiwgdGlzc3VlUmVnaW9uLCBQYXRpZW50X0lEKQp0ZXN0X2dyX2ZpbHRlcmVkIDwtIGdhdGhlcl9zZXRfZGF0YShjb3VudF9tYXRfZmlsdGVyZWQsIDE6NSkKdGVzdF9ncl9maWx0ZXJlZCR4IDwtIGZhY3Rvcih0ZXN0X2dyX2ZpbHRlcmVkJHgsCiAgICAgICAgICAgICAgICAgICAgICAgICAgICAgbGFiZWxzID0gYygnc2xpZGUgbmFtZScsICdQYXRpZW50X0lEJywgJ3NsaWRlTG9jYXRpb24nLCAnc2VnbWVudCcsICd0aXNzdWVSZWdpb24nKSkKYGBgCgpyZS1QbG90IFNhbmtleQoKYGBge3IgcGxvdF9zYW5rZXksIGZpZy53aWR0aD0yMCxmaWcuaGVpZ2h0PTExfQp0ZXN0X2dyX2ZpbHRlcmVkICU+JQogIGdncGxvdCguLCBoZWlnaHQgPSAxMCwgd2lkdGggPSAxMCwgYWVzKHgsIGlkID0gaWQsIHNwbGl0ID0geSwgdmFsdWUgPSBuKSkgKwogIGdlb21fcGFyYWxsZWxfc2V0cyhhZXMoZmlsbCA9IHNlZ21lbnQpLCBhbHBoYSA9IDAuNSwgYXhpcy53aWR0aCA9IDAuNDUpICsKICBnZW9tX3BhcmFsbGVsX3NldHNfYXhlcyhhZXMoZmlsbCA9IGlmX2Vsc2Uoc2VnbWVudCA9PSB5LCBzZWdtZW50LCAnZ3JleScpKSwKICAgICAgICAgICAgICAgICAgICAgICAgICBheGlzLndpZHRoID0gMC40NSwgY29sb3IgPSAnYmxhY2snLCBmaWxsID0gJ2dyZXknKSArCiAgZ2VvbV9wYXJhbGxlbF9zZXRzX2xhYmVscyhjb2xvciA9ICdibGFjaycsIHNpemUgPSA1LCBhbmdsZSA9IDApICsKICBzY2FsZV95X2NvbnRpbnVvdXMoZXhwYW5kID0gZXhwYW5zaW9uKDApKSArCiAgc2NhbGVfeF9kaXNjcmV0ZShleHBhbmQgPSBleHBhbnNpb24oMCksIGxhYmVscyA9IGFubl9zZWxlY3Rpb24kYW5ub3RhdGlvbl9sYWJlbHMsIHBvc2l0aW9uID0gJ3RvcCcpICsKICBsYWJzKHggPSAnJywgeSA9ICcnKSArCiAgc2NhbGVfZmlsbF9tYW51YWwodmFsdWVzID0gYW5uX2NvbG9ycyRzZWdtZW50KSArCiAgYW5ub3RhdGUoZ2VvbSA9ICdzZWdtZW50JywgeCA9IDQuNCwgeGVuZCA9IDQuNCwKICAgICAgICAgICB5ID0gMTAsIHllbmQgPSAxMTAsIGx3ZCA9IDIpICsKICBhbm5vdGF0ZShnZW9tID0gJ3RleHQnLCB4ID0gNC4zNCwgeSA9IDUwLCBhbmdsZSA9IDkwLCBzaXplID0gNSwKICAgICAgICAgICBoanVzdCA9IDAuNSwgbGFiZWwgPSAnMTAwIHNlZ21lbnRzJykgKwogIHRoZW1lX2NsYXNzaWMoYmFzZV9zaXplID0gMTIpICsKICB0aGVtZShsZWdlbmQucG9zaXRpb24gPSAnbm9uZScsCiAgICAgICAgbGVnZW5kLnRpdGxlID0gZWxlbWVudF9ibGFuaygpLAogICAgICAgIGxlZ2VuZC50ZXh0ID0gZWxlbWVudF90ZXh0KHNpemUgPSAxNSksCiAgICAgICAgcGFuZWwuYmFja2dyb3VuZCA9IGVsZW1lbnRfcmVjdChmaWxsID0gJ3doaXRlJyksCiAgICAgICAgYXhpcy50aWNrcy54ID0gZWxlbWVudF9ibGFuaygpLAogICAgICAgIGF4aXMudGlja3MueSA9IGVsZW1lbnRfYmxhbmsoKSwKICAgICAgICBheGlzLmxpbmUgPSBlbGVtZW50X2JsYW5rKCksCiAgICAgICAgIyBheGlzLnRleHQueCA9IGVsZW1lbnRfYmxhbmsoKSwKICAgICAgICBheGlzLnRleHQueCA9IGVsZW1lbnRfdGV4dChzaXplID0gMTYsIGZhY2UgPSAnYm9sZCcpLAogICAgICAgIGF4aXMudGV4dC55ID0gZWxlbWVudF9ibGFuaygpLAogICAgICAgIHBsb3QubWFyZ2luID0gbWFyZ2luKDEsMSwxLDEsIHVuaXQgPSAnY20nKSkKYGBgCgojIyA0LjYgR2VuZSBMZXZlbCBRQwoKIyMjIENhbGN1bGF0ZSBnZW5lIGRldGVjdGlvbiByYXRlCgpgYGB7ciBjYWxjX2RldGVjdGlvbl9yYXRlLCBtZXNzYWdlPUZBTFNFfQpMT1FfTWF0IDwtIExPUV9NYXRbLCBjb2xuYW1lcyh0YXJnZXRfRGF0YSldCgpmRGF0YSh0YXJnZXRfRGF0YSkkRGV0ZWN0ZWRTZWdtZW50cyA8LSByb3dTdW1zKExPUV9NYXQsIG5hLnJtID0gVFJVRSkKZkRhdGEodGFyZ2V0X0RhdGEpJERldGVjdGlvblJhdGUgPC0gZkRhdGEodGFyZ2V0X0RhdGEpJERldGVjdGVkU2VnbWVudHMgLyBucm93KHBEYXRhKHRhcmdldF9EYXRhKSkKYGBgCgpDaGVjayB3aGljaCBnZW5lcyBhcmUgZmFpbGluZyB0byBiZSBkZXRlY3RlZCBhY3Jvc3MgZGlmZmVyZW50IGFubm90YXRpb24gZmVhdHVyZXMKCmBgYHtyLCBpbmNsdWRlPUZBTFNFLCBldmFsPUZBTFNFfQojIHJlYWQgUEtDIGZpbGUgaW50byBSIHRvIGdldCB0aGUgdGFyZ2V0IGdlbmUgbmFtZXMKd3RhX2hzX2pzb24gPC0gZnJvbUpTT04oZmlsZSA9IHBhc3RlMChiYXNlX2RpciwgJy9wcm9jZXNzZWQvbmFub3N0cmluZy8nLCBpbnB1dF9leHBlcmltZW50LCAnL3BrY3MvJywgcGtjcykpCgp3dGFfaHNfZGYgPC0gdGliYmxlKGRhdGEgPSB3dGFfaHNfanNvbiRUYXJnZXRzKQp3dGFfaHNfZGYgPC0gdW5uZXN0X3dpZGVyKHd0YV9oc19kZiwgY29sID0gZGF0YSkKd3RhX2hzX2RmIDwtIHNlbGVjdCh3dGFfaHNfZGYsIERpc3BsYXlOYW1lKSAlPiUgcmVuYW1lKERpc3BsYXlOYW1lID0gJ1RhcmdldE5hbWUnKQoKcGRhdGFfTE9RIDwtIHBEYXRhKHRhcmdldF9EYXRhKSAlPiUKICByb3duYW1lc190b19jb2x1bW4odmFyID0gJ1NhbXBsZV9JRCcpICU+JQogIGdyb3VwX2J5KHNlZ21lbnQpICU+JQogIG11dGF0ZShuU2VnbWVudCA9IG4oKSkgJT4lCiAgZ3JvdXBfYnkoYHNsaWRlIG5hbWVgKSAlPiUKICBtdXRhdGUoblNsaWRlID0gbigpKSAlPiUKICBncm91cF9ieSh0aXNzdWVSZWdpb24pICU+JQogIG11dGF0ZShuUmVnaW9uID0gbigpKSAlPiUKICBncm91cF9ieShQYXRpZW50X0lEKSAlPiUKICBtdXRhdGUoblBhdGllbnQgPSBuKCkpICU+JQogIHVuZ3JvdXAoKQoKZmFpbGVkX0xPUSA8LSBhcy5kYXRhLmZyYW1lKHQoTE9RX01hdCkpICU+JQogIHJvd25hbWVzX3RvX2NvbHVtbih2YXIgPSAnU2FtcGxlX0lEJykgJT4lCiAgcGl2b3RfbG9uZ2VyKDI6bGFzdF9jb2woKSwgbmFtZXNfdG8gPSAnVGFyZ2V0cycsIHZhbHVlc190byA9ICdQYXNzX0xPUScpICU+JQogIGZpbHRlcighUGFzc19MT1EpICU+JQogIGlubmVyX2pvaW4ocGRhdGFfTE9RLCBieSA9ICdTYW1wbGVfSUQnKQoKZmFpbGVkX0xPUV9zbGlkZSA8LSBmYWlsZWRfTE9RICU+JQogIGdyb3VwX2J5KFRhcmdldHMsIGBzbGlkZSBuYW1lYCwgblNsaWRlKSAlPiUKICBzdW1tYXJpc2UobiA9IG4oKSwgLmdyb3VwcyA9ICdkcm9wJykgJT4lCiAgbXV0YXRlKHBlcmNfRkFJTCA9IG4gLyBuU2xpZGUgKiAxMDApICU+JQogIHNlbGVjdChUYXJnZXRzLCBgc2xpZGUgbmFtZWAsIHBlcmNfRkFJTCkgJT4lCiAgcGl2b3Rfd2lkZXIobmFtZXNfZnJvbSA9IGMoJ3NsaWRlIG5hbWUnKSwgdmFsdWVzX2Zyb20gPSBjKCdwZXJjX0ZBSUwnKSwgbmFtZXNfcHJlZml4ID0gJ3BlcmNfRkFJTF8nKSAlPiUKICBtdXRhdGUoYWNyb3NzKHdoZXJlKGlzLm51bWVyaWMpLCB+aWZfZWxzZShpcy5uYSgueCksIDAsIC54KSkpICU+JQogIHNlbWlfam9pbih3dGFfaHNfZGYsIGJ5ID0gYygnVGFyZ2V0cycgPSAnVGFyZ2V0TmFtZScpKQoKZmFpbGVkX0xPUV9wYXRpZW50IDwtIGZhaWxlZF9MT1EgJT4lCiAgZ3JvdXBfYnkoVGFyZ2V0cywgUGF0aWVudF9JRCwgblBhdGllbnQpICU+JQogIHN1bW1hcmlzZShuID0gbigpLCAuZ3JvdXBzID0gJ2Ryb3AnKSAlPiUKICBtdXRhdGUocGVyY19GQUlMID0gbiAvIG5QYXRpZW50ICogMTAwKSAlPiUKICBzZWxlY3QoVGFyZ2V0cywgUGF0aWVudF9JRCwgcGVyY19GQUlMKSAlPiUKICBwaXZvdF93aWRlcihuYW1lc19mcm9tID0gYygnUGF0aWVudF9JRCcpLCB2YWx1ZXNfZnJvbSA9IGMoJ3BlcmNfRkFJTCcpLCBuYW1lc19wcmVmaXggPSAncGVyY19GQUlMXycpICU+JQogIG11dGF0ZShhY3Jvc3Mod2hlcmUoaXMubnVtZXJpYyksIH5pZl9lbHNlKGlzLm5hKC54KSwgMCwgLngpKSkgJT4lCiAgc2VtaV9qb2luKHd0YV9oc19kZiwgYnkgPSBjKCdUYXJnZXRzJyA9ICdUYXJnZXROYW1lJykpCgpmYWlsZWRfTE9RX3NlZ21lbnQgPC0gZmFpbGVkX0xPUSAlPiUKICBncm91cF9ieShUYXJnZXRzLCBzZWdtZW50LCBuU2VnbWVudCkgJT4lCiAgc3VtbWFyaXNlKG4gPSBuKCksIC5ncm91cHMgPSAnZHJvcCcpICU+JQogIG11dGF0ZShwZXJjX0ZBSUwgPSBuIC8gblNlZ21lbnQgKiAxMDApICU+JQogIHNlbGVjdChUYXJnZXRzLCBzZWdtZW50LCBwZXJjX0ZBSUwpICU+JQogIHBpdm90X3dpZGVyKG5hbWVzX2Zyb20gPSBjKCdzZWdtZW50JyksIHZhbHVlc19mcm9tID0gYygncGVyY19GQUlMJyksIG5hbWVzX3ByZWZpeCA9ICdwZXJjX0ZBSUxfJykgJT4lCiAgbXV0YXRlKGFjcm9zcyh3aGVyZShpcy5udW1lcmljKSwgfmlmX2Vsc2UoaXMubmEoLngpLCAwLCAueCkpKSAlPiUKICBzZW1pX2pvaW4od3RhX2hzX2RmLCBieSA9IGMoJ1RhcmdldHMnID0gJ1RhcmdldE5hbWUnKSkKCmZhaWxlZF9MT1FfcmVnaW9uIDwtIGZhaWxlZF9MT1EgJT4lCiAgZ3JvdXBfYnkoVGFyZ2V0cywgdGlzc3VlUmVnaW9uLCBuUmVnaW9uKSAlPiUKICBzdW1tYXJpc2UobiA9IG4oKSwgLmdyb3VwcyA9ICdkcm9wJykgJT4lCiAgbXV0YXRlKHBlcmNfRkFJTCA9IG4gLyBuUmVnaW9uICogMTAwKSAlPiUKICBzZWxlY3QoVGFyZ2V0cywgdGlzc3VlUmVnaW9uLCBwZXJjX0ZBSUwpICU+JQogIHBpdm90X3dpZGVyKG5hbWVzX2Zyb20gPSBjKCd0aXNzdWVSZWdpb24nKSwgdmFsdWVzX2Zyb20gPSBjKCdwZXJjX0ZBSUwnKSwgbmFtZXNfcHJlZml4ID0gJ3BlcmNfRkFJTF8nKSAlPiUKICBtdXRhdGUoYWNyb3NzKHdoZXJlKGlzLm51bWVyaWMpLCB+aWZfZWxzZShpcy5uYSgueCksIDAsIC54KSkpICU+JQogIHNlbWlfam9pbih3dGFfaHNfZGYsIGJ5ID0gYygnVGFyZ2V0cycgPSAnVGFyZ2V0TmFtZScpKQoKcm0oZmFpbGVkX0xPUSkKIyBnb29nbGVzaGVldHM0OjpnczRfY3JlYXRlKAojICAgbmFtZSA9ICdmYWlsZWRfTE9RX3RhcmdldHNfbjAnLAojICAgc2hlZXRzID0gYygndGFyZ2V0cycsICdwZXJjX3Blcl9zbGlkZScsICdwZXJjX3Blcl9wYXRpZW50JywgJ3BlcmNfcGVyX3JlZ2lvbicsICdwZXJjX3Blcl9zZWdtZW50JykKIyApCiMgCiMgZ29vZ2xlc2hlZXRzNDo6d3JpdGVfc2hlZXQoCiMgICBkYXRhID0gd3RhX2hzX2RmLAojICAgIyBzcyA9ICdodHRwczovL2RvY3MuZ29vZ2xlLmNvbS9zcHJlYWRzaGVldHMvZC8xbWtDcjlZMFlLWlR3VkxJZHlOQW5vSDZOYUN5UzhuTVhnX2dEWXpXRkptby9lZGl0P3VzcD1zaGFyaW5nJywgIyBuID0gMgojICAgIyBzcyA9ICdodHRwczovL2RvY3MuZ29vZ2xlLmNvbS9zcHJlYWRzaGVldHMvZC8xSHlGS2ljTmQwMzg0azRtWlRZRkVCenkwbjlyT044UmJpTUVGb3JldlU5VS9lZGl0P3VzcD1zaGFyaW5nJywgIyBuPSAxLjUKIyAgICMgc3MgPSAnaHR0cHM6Ly9kb2NzLmdvb2dsZS5jb20vc3ByZWFkc2hlZXRzL2QvMWQxVFFFVFVxaVZVTUJfR2RnNng0cE84VmN2LWpRb0ZfQlY2M2VDaTBGc0EvZWRpdD91c3A9c2hhcmluZycsICMgbiA9IDEKIyAgIHNzID0gJ2h0dHBzOi8vZG9jcy5nb29nbGUuY29tL3NwcmVhZHNoZWV0cy9kLzFuLXo5M3pjZVdDdEROUWNCNVFjZGZQN2hQRkpKb2szVHBSVUFzd2NPY3o4L2VkaXQ/dXNwPXNoYXJpbmcnLCAjIG4gPSAwLjUKIyAgICMgc3MgPSAnaHR0cHM6Ly9kb2NzLmdvb2dsZS5jb20vc3ByZWFkc2hlZXRzL2QvMWp6SEZQSUJSM1U5dFh1ck96Y2VXMGhzVERwSkJWME43UnVaNkNpV3pveEEvZWRpdD91c3A9c2hhcmluZycsICMgbiA9IDAKIyAgIHNoZWV0ID0gJ3RhcmdldHMnCiMgKQojIGdvb2dsZXNoZWV0czQ6OndyaXRlX3NoZWV0KAojICAgZGF0YSA9IGZhaWxlZF9MT1Ffc2xpZGUsCiMgICAjIHNzID0gJ2h0dHBzOi8vZG9jcy5nb29nbGUuY29tL3NwcmVhZHNoZWV0cy9kLzFta0NyOVkwWUtaVHdWTElkeU5Bbm9INk5hQ3lTOG5NWGdfZ0RZeldGSm1vL2VkaXQ/dXNwPXNoYXJpbmcnLCAjIG4gPSAyCiMgICAjIHNzID0gJ2h0dHBzOi8vZG9jcy5nb29nbGUuY29tL3NwcmVhZHNoZWV0cy9kLzFIeUZLaWNOZDAzODRrNG1aVFlGRUJ6eTBuOXJPTjhSYmlNRUZvcmV2VTlVL2VkaXQ/dXNwPXNoYXJpbmcnLCAjIG49IDEuNQojICAgIyBzcyA9ICdodHRwczovL2RvY3MuZ29vZ2xlLmNvbS9zcHJlYWRzaGVldHMvZC8xZDFUUUVUVXFpVlVNQl9HZGc2eDRwTzhWY3YtalFvRl9CVjYzZUNpMEZzQS9lZGl0P3VzcD1zaGFyaW5nJywgIyBuID0gMQojICAgc3MgPSAnaHR0cHM6Ly9kb2NzLmdvb2dsZS5jb20vc3ByZWFkc2hlZXRzL2QvMW4tejkzemNlV0N0RE5RY0I1UWNkZlA3aFBGSkpvazNUcFJVQXN3Y09jejgvZWRpdD91c3A9c2hhcmluZycsICMgbiA9IDAuNQojICAgIyBzcyA9ICdodHRwczovL2RvY3MuZ29vZ2xlLmNvbS9zcHJlYWRzaGVldHMvZC8xanpIRlBJQlIzVTl0WHVyT3pjZVcwaHNURHBKQlYwTjdSdVo2Q2lXem94QS9lZGl0P3VzcD1zaGFyaW5nJywgIyBuID0gMAojICAgc2hlZXQgPSAncGVyY19wZXJfc2xpZGUnCiMgKQojIGdvb2dsZXNoZWV0czQ6OndyaXRlX3NoZWV0KAojICAgZGF0YSA9IGZhaWxlZF9MT1FfcGF0aWVudCwKIyAgICMgc3MgPSAnaHR0cHM6Ly9kb2NzLmdvb2dsZS5jb20vc3ByZWFkc2hlZXRzL2QvMW1rQ3I5WTBZS1pUd1ZMSWR5TkFub0g2TmFDeVM4bk1YZ19nRFl6V0ZKbW8vZWRpdD91c3A9c2hhcmluZycsICMgbiA9IDIKIyAgICMgc3MgPSAnaHR0cHM6Ly9kb2NzLmdvb2dsZS5jb20vc3ByZWFkc2hlZXRzL2QvMUh5RktpY05kMDM4NGs0bVpUWUZFQnp5MG45ck9OOFJiaU1FRm9yZXZVOVUvZWRpdD91c3A9c2hhcmluZycsICMgbj0gMS41CiMgICAjIHNzID0gJ2h0dHBzOi8vZG9jcy5nb29nbGUuY29tL3NwcmVhZHNoZWV0cy9kLzFkMVRRRVRVcWlWVU1CX0dkZzZ4NHBPOFZjdi1qUW9GX0JWNjNlQ2kwRnNBL2VkaXQ/dXNwPXNoYXJpbmcnLCAjIG4gPSAxCiMgICBzcyA9ICdodHRwczovL2RvY3MuZ29vZ2xlLmNvbS9zcHJlYWRzaGVldHMvZC8xbi16OTN6Y2VXQ3RETlFjQjVRY2RmUDdoUEZKSm9rM1RwUlVBc3djT2N6OC9lZGl0P3VzcD1zaGFyaW5nJywgIyBuID0gMC41CiMgICAjIHNzID0gJ2h0dHBzOi8vZG9jcy5nb29nbGUuY29tL3NwcmVhZHNoZWV0cy9kLzFqekhGUElCUjNVOXRYdXJPemNlVzBoc1REcEpCVjBON1J1WjZDaVd6b3hBL2VkaXQ/dXNwPXNoYXJpbmcnLCAjIG4gPSAwCiMgICBzaGVldCA9ICdwZXJjX3Blcl9wYXRpZW50JwojICkKIyBnb29nbGVzaGVldHM0Ojp3cml0ZV9zaGVldCgKIyAgIGRhdGEgPSBmYWlsZWRfTE9RX3JlZ2lvbiwKIyAgICMgc3MgPSAnaHR0cHM6Ly9kb2NzLmdvb2dsZS5jb20vc3ByZWFkc2hlZXRzL2QvMW1rQ3I5WTBZS1pUd1ZMSWR5TkFub0g2TmFDeVM4bk1YZ19nRFl6V0ZKbW8vZWRpdD91c3A9c2hhcmluZycsICMgbiA9IDIKIyAgICMgc3MgPSAnaHR0cHM6Ly9kb2NzLmdvb2dsZS5jb20vc3ByZWFkc2hlZXRzL2QvMUh5RktpY05kMDM4NGs0bVpUWUZFQnp5MG45ck9OOFJiaU1FRm9yZXZVOVUvZWRpdD91c3A9c2hhcmluZycsICMgbj0gMS41CiMgICAjIHNzID0gJ2h0dHBzOi8vZG9jcy5nb29nbGUuY29tL3NwcmVhZHNoZWV0cy9kLzFkMVRRRVRVcWlWVU1CX0dkZzZ4NHBPOFZjdi1qUW9GX0JWNjNlQ2kwRnNBL2VkaXQ/dXNwPXNoYXJpbmcnLCAjIG4gPSAxCiMgICBzcyA9ICdodHRwczovL2RvY3MuZ29vZ2xlLmNvbS9zcHJlYWRzaGVldHMvZC8xbi16OTN6Y2VXQ3RETlFjQjVRY2RmUDdoUEZKSm9rM1RwUlVBc3djT2N6OC9lZGl0P3VzcD1zaGFyaW5nJywgIyBuID0gMC41CiMgICAjIHNzID0gJ2h0dHBzOi8vZG9jcy5nb29nbGUuY29tL3NwcmVhZHNoZWV0cy9kLzFqekhGUElCUjNVOXRYdXJPemNlVzBoc1REcEpCVjBON1J1WjZDaVd6b3hBL2VkaXQ/dXNwPXNoYXJpbmcnLCAjIG4gPSAwCiMgICBzaGVldCA9ICdwZXJjX3Blcl9yZWdpb24nCiMgKQojIGdvb2dsZXNoZWV0czQ6OndyaXRlX3NoZWV0KAojICAgZGF0YSA9IGZhaWxlZF9MT1Ffc2VnbWVudCwKIyAgICMgc3MgPSAnaHR0cHM6Ly9kb2NzLmdvb2dsZS5jb20vc3ByZWFkc2hlZXRzL2QvMW1rQ3I5WTBZS1pUd1ZMSWR5TkFub0g2TmFDeVM4bk1YZ19nRFl6V0ZKbW8vZWRpdD91c3A9c2hhcmluZycsICMgbiA9IDIKIyAgICMgc3MgPSAnaHR0cHM6Ly9kb2NzLmdvb2dsZS5jb20vc3ByZWFkc2hlZXRzL2QvMUh5RktpY05kMDM4NGs0bVpUWUZFQnp5MG45ck9OOFJiaU1FRm9yZXZVOVUvZWRpdD91c3A9c2hhcmluZycsICMgbj0gMS41CiMgICAjIHNzID0gJ2h0dHBzOi8vZG9jcy5nb29nbGUuY29tL3NwcmVhZHNoZWV0cy9kLzFkMVRRRVRVcWlWVU1CX0dkZzZ4NHBPOFZjdi1qUW9GX0JWNjNlQ2kwRnNBL2VkaXQ/dXNwPXNoYXJpbmcnLCAjIG4gPSAxCiMgICBzcyA9ICdodHRwczovL2RvY3MuZ29vZ2xlLmNvbS9zcHJlYWRzaGVldHMvZC8xbi16OTN6Y2VXQ3RETlFjQjVRY2RmUDdoUEZKSm9rM1RwUlVBc3djT2N6OC9lZGl0P3VzcD1zaGFyaW5nJywgIyBuID0gMC41CiMgICAjIHNzID0gJ2h0dHBzOi8vZG9jcy5nb29nbGUuY29tL3NwcmVhZHNoZWV0cy9kLzFqekhGUElCUjNVOXRYdXJPemNlVzBoc1REcEpCVjBON1J1WjZDaVd6b3hBL2VkaXQ/dXNwPXNoYXJpbmcnLCAjIG4gPSAwCiMgICBzaGVldCA9ICdwZXJjX3Blcl9zZWdtZW50JwojICkKYGBgCgoKR2VuZSBvZiBpbnRlcmVzdCBkZXRlY3Rpb24gdGFibGU6CgpgYGB7ciBnZW5lX29mX2ludGVyZXN0X3RhYmxlfQojIEdlbmUgb2YgaW50ZXJlc3QgZGV0ZWN0aW9uIHRhYmxlCmdvaSA8LSBjKCdBQ1RBMicsICdQREdGUkEnLCAnUERHRlJCJywgJ0lMNicsICdGQVAnLCAnVE5GJywnQ1hDTDE0JywgJ1NGUlAxJywgJ1JTUE8zJywgJ0dSRU0xJywgJ0VNUDEnLCAnTEdSNScsCiAgICAgICAgICAgICAgICAgICAnU0ZSUDInLCAnU0ZSUDQnLCAnVEdGQjEnLCAnVEdGQjMnLCAnR0xJMScsICdCTVA0JywgJ01NUDcnLCAnTU1QMScsICdNTVAzJywgJ1JTUE8xJywgJ1JTUE8yJywgJ1JTUE80JykKZ29pX2RmIDwtIGRhdGEuZnJhbWUoCiAgICBHZW5lID0gZ29pLAogICAgTnVtYmVyID0gZkRhdGEodGFyZ2V0X0RhdGEpW2dvaSwgIkRldGVjdGVkU2VnbWVudHMiXSwKICAgIERldGVjdGlvblJhdGUgPSBwZXJjZW50KGZEYXRhKHRhcmdldF9EYXRhKVtnb2ksICJEZXRlY3Rpb25SYXRlIl0pKSAlPiUKICBhcnJhbmdlKEdlbmUpCgojIHdyaXRlIHRvIGRpc2sKaWYgKHNhdmVfdGFibGVzKSB7CiAgd3JpdGVfdHN2KAogICAgZ29pX2RmLAogICAgZmlsZSA9IHBhc3RlMChvdXRwdXRfZGlyLCAnLycsIGlucHV0X2V4cGVyaW1lbnQsICcvb3V0cHV0L3FjX3Jlc3VsdHMvJywgY3VycmVudF9kYXRlLCAnL2dvaV9kZXRlY3Rpb25fcmF0ZS50c3YnKQogICkKfQpgYGAKCiMjIyBHZW5lIEZpbHRlcmluZwoKV2Ugd2lsbCBncmFwaCB0aGUgdG90YWwgbnVtYmVyIG9mIGdlbmVzIGRldGVjdGVkIGluIGRpZmZlcmVudApwZXJjZW50YWdlcyBvZiBzZWdtZW50cy4gQmFzZWQgb24gdGhlIHZpc3VhbGl6YXRpb24gYmVsb3csIHdlIGNhbiBiZXR0ZXIKdW5kZXJzdGFuZCBnbG9iYWwgZ2VuZSBkZXRlY3Rpb24gaW4gb3VyIHN0dWR5IGFuZCBzZWxlY3QgaG93IG1hbnkgbG93CmRldGVjdGVkIGdlbmVzIHRvIGZpbHRlciBvdXQgb2YgdGhlIGRhdGFzZXQuIEdlbmUgZmlsdGVyaW5nIGluY3JlYXNlcwpwZXJmb3JtYW5jZSBvZiBkb3duc3RyZWFtIHN0YXRpc3RpY2FsIHRlc3RzIGFuZCBpbXByb3ZlcyBpbnRlcnByZXRhdGlvbgpvZiB0cnVlIGJpb2xvZ2ljYWwgc2lnbmFsLjxicj48YnI+CgpQbG90IGRldGVjdGlvbiByYXRlCgpgYGB7ciBwbG90X2RldF9yYXRlfQpwbG90X2RldGVjdCA8LSBkYXRhLmZyYW1lKEZyZXEgPSBjKDAsMSwgMiwgNSwgMTAsIDIwLCAzMCwgNTAsIDc1LCA5MCwgMTAwKSkKcGxvdF9kZXRlY3QkTnVtYmVyIDwtIHVubGlzdChsYXBwbHkoYygwLDAuMDEsIDAuMDIsIDAuMDUsIDAuMSwgMC4yLCAwLjMsIDAuNSwgMC43NSwgMC45LCAxKSwKICAgICAgICAgICAgICAgICAgICAgICAgICAgICAgICAgICAgZnVuY3Rpb24oeCkgewogICAgICAgICAgICAgICAgICAgICAgICAgICAgICAgICAgICAgIHN1bShmRGF0YSh0YXJnZXRfRGF0YSkkRGV0ZWN0aW9uUmF0ZSA+PSB4KQogICAgICAgICAgICAgICAgICAgICAgICAgICAgICAgICAgICAgIH0pKQpwbG90X2RldGVjdCRSYXRlIDwtIHBsb3RfZGV0ZWN0JE51bWJlciAvIG5yb3coZkRhdGEodGFyZ2V0X0RhdGEpKQpyb3duYW1lcyhwbG90X2RldGVjdCkgPC0gcGxvdF9kZXRlY3QkRnJlcQoKZ2dwbG90KHBsb3RfZGV0ZWN0LAogICAgICAgYWVzKAogICAgICAgICB4ID0gYXMuZmFjdG9yKEZyZXEpLAogICAgICAgICB5ID0gUmF0ZSwKICAgICAgICAgZmlsbCA9IFJhdGUpKSArCiAgZ2VvbV9iYXIoc3RhdCA9ICdpZGVudGl0eScpICsKICBnZW9tX3RleHQoCiAgICBhZXMobGFiZWwgPSBmb3JtYXRDKE51bWJlciwgZm9ybWF0ID0gJ2QnLCBiaWcubWFyayA9ICcsJykpLAogICAgdmp1c3QgPSAxLjYsCiAgICBjb2xvciA9ICdibGFjaycsCiAgICBzaXplID0gNCkgKwogIHNjYWxlX2ZpbGxfZ3JhZGllbnQyKAogICAgbG93ID0gJ29yYW5nZTInLAogICAgbWlkID0gJ2xpZ2h0Ymx1ZScsCiAgICBoaWdoID0gJ2RvZGdlcmJsdWUzJywKICAgIG1pZHBvaW50ID0gMC42NSwKICAgIGxpbWl0cyA9IGMoMCwxKSwKICAgIGxhYmVscyA9IHNjYWxlczo6cGVyY2VudCkgKwogIHRoZW1lX2J3KCkgKwogIHNjYWxlX3lfY29udGludW91cygKICAgIGxhYmVscyA9IHNjYWxlczo6cGVyY2VudCwKICAgIGxpbWl0cyA9IGMoMCwxKSwKICAgIGV4cGFuZCA9IGV4cGFuc2lvbihtdWx0ID0gYygwLCAwKSkpICsKICBsYWJzKHggPSAnJSBvZiBzZWdtZW50cycsCiAgICAgICB5ID0gJ0dlbmVzIERldGVjdGVkLCAlIG9mIFBhbmVsID4gTE9RJykKYGBgCgpTdWJzZXQgdG8gdGFyZ2V0IGdlbmVzIGRldGVjdGVkIGluIGF0IGxlYXN0CmByIHJvdW5kKGdlbmVfZGV0X3RocmVzaG9sZCAqIDEwMCwgMClgJSBvZiB0aGUgc2FtcGxlcyAoZGVmYXVsdDogPj0xMCUgb2Ygc2VnbWVudHMpLiBBbHNvIG1hbnVhbGx5CmluY2x1ZGUgdGhlIG5lZ2F0aXZlIGNvbnRyb2wgcHJvYmUsIGZvciBkb3duc3RyZWFtIHVzZS4KCmBgYHtyIHN1YnNldF90b19wX2RldGVjdGVkX2dlbmVzfQojIGNhbGN1bGF0ZSBob3cgbWFueSBzZWdtZW50cyBhcmUgbG9zdCBkdWUgdG8gZ2VuZSBkZXRlY3Rpb24gdGhyZXNob2xkCm5HZW5lc19wcmVmaWx0ZXJfTE9RIDwtIHVubmFtZShucm93KHRhcmdldF9EYXRhKSkKCiMgZmlsdGVyIG91dCBnZW5lcyB0aGF0IGFyZSBmb3VuZCBpbiBsZXNzIHRoYW4gTiAlIG9mIHNlZ21lbnRzIGR1ZSB0byBiZWxvdy1MT1EgZXhwcmVzc2lvbgojIGRlZmF1bHQgPSAwLjEgKD49MTAlIG9mIHNlZ21lbnRzKQpuZWdhdGl2ZVByb2JlZkRhdGEgPC0gc3Vic2V0KGZEYXRhKHRhcmdldF9EYXRhKSwgQ29kZUNsYXNzID09ICdOZWdhdGl2ZScpCm5lZ19wcm9iZXMgPC0gdW5pcXVlKG5lZ2F0aXZlUHJvYmVmRGF0YSRUYXJnZXROYW1lKQp0YXJnZXRfRGF0YSA8LSB0YXJnZXRfRGF0YVtmRGF0YSh0YXJnZXRfRGF0YSkkRGV0ZWN0aW9uUmF0ZSA+PSBnZW5lX2RldF90aHJlc2hvbGQgfCBmRGF0YSh0YXJnZXRfRGF0YSkkVGFyZ2V0TmFtZSAlaW4lIG5lZ19wcm9iZXMsIF0KCiMgY2FsY3VsYXRlIGhvdyBtYW55IHNlZ21lbnRzIGFyZSBsb3N0IGR1ZSB0byBnZW5lIGRldGVjdGlvbiB0aHJlc2hvbGQKbkdlbmVzX3Bvc3RmaWx0ZXJfTE9RIDwtIHVubmFtZShucm93KHRhcmdldF9EYXRhKSkKbkdlbmVzX2xvc3RfTE9RIDwtIG5HZW5lc19wcmVmaWx0ZXJfTE9RIC0gbkdlbmVzX3Bvc3RmaWx0ZXJfTE9RCgpkaW0odGFyZ2V0X0RhdGEpCgojIHJldGFpbiBvbmx5IGRldGVjdGVkIGdlbmVzIG9mIGludGVyZXN0CiMgZ29pIDwtIGdvaVtnb2kgJWluJSByb3duYW1lcyh0YXJnZXRfRGF0YSldCmBgYAoKQWZ0ZXIgZmlsdGVyaW5nIHRoZSBnZW5lcyBieSBnZW5lIGRldGVjdGlvbiByYXRlIGJhc2VkIG9uIExPUSwgdGhlcmUgd2VyZSBgciBuR2VuZXNfbG9zdF9MT1FgIGdlbmVzIGxvc3QgKHByZWZpbHRlcjogYHIgbkdlbmVzX3ByZWZpbHRlcl9MT1FgLCBwb3N0ZmlsdGVyOiBgciBuR2VuZXNfcG9zdGZpbHRlcl9MT1FgKS4KCmBgYHtyIHNhdmVfdGFyZ2V0X0RhdGF9CiMgc2F2ZSBmaWx0ZXJlZCBvYmplY3QKc2F2ZVJEUygKICBvYmplY3QgPSB0YXJnZXRfRGF0YSwKICBmaWxlID0gcGFzdGUwKG91dHB1dF9kaXIsICcvJywgaW5wdXRfZXhwZXJpbWVudCwgJy9vdXRwdXQvcWNfcmVzdWx0cy8nLCBjdXJyZW50X2RhdGUsICcvdGFyZ2V0X0RhdGEuUkRTJykKKQoKYGBgCgo=
